# Evolutionary dynamics on sequential temporal networks

**DOI:** 10.1371/journal.pcbi.1011333

**Published:** 2023-08-07

**Authors:** Anzhi Sheng, Aming Li, Long Wang

**Affiliations:** 1 Center for Systems and Control, College of Engineering, Peking University, Beijing, China; 2 Department of Biology, University of Pennsylvania, Philadelphia, United States of America; 3 Center for Multi-Agent Research, Institute for Artificial Intelligence, Peking University, Beijing, China; University of Zaragoza: Universidad de Zaragoza, SPAIN

## Abstract

Population structure is a well-known catalyst for the evolution of cooperation and has traditionally been considered to be static in the course of evolution. Conversely, real-world populations, such as microbiome communities and online social networks, frequently show a progression from tiny, active groups to huge, stable communities, which is insufficient to be captured by constant structures. Here, we propose sequential temporal networks to characterize growing networked populations, and we extend the theory of evolutionary games to these temporal networks with arbitrary structures and growth rules. We derive analytical rules under which a sequential temporal network has a higher fixation probability for cooperation than its static counterpart. Under neutral drift, the rule is simply a function of the increment of nodes and edges in each time step. But if the selection is weak, the rule is related to coalescence times on networks. In this case, we propose a mean-field approximation to calculate fixation probabilities and critical benefit-to-cost ratios with lower calculation complexity. Numerical simulations in empirical datasets also prove the cooperation-promoting effect of population growth. Our research stresses the significance of population growth in the real world and provides a high-accuracy approximation approach for analyzing the evolution in real-life systems.

## Author summary

The temporality of real-world populations often arises from the growth in the number of individuals and links. Such dynamical systems cannot be adequately represented by a single static network. Here, we use sequential temporal networks to characterize time-varying interactions in growing populations and propose a method for analyzing evolutionary dynamics over these networks with arbitrary structures and growth rules. We find that cooperation can be favored in sequential temporal networks under neutral drift when cooperators form clusters or become hub nodes before new intruders (defectors) enter the populations. These conditions ensure the smooth dissemination of cooperation among individuals. We also derive the corresponding condition under weak selection, which is related to coalescence times on networks. At the same time, we provide a mean-field approximation approach for measuring the cooperation-promoting effect of large-scale sequential temporal networks. Through numerical simulations in empirical datasets from different realistic contexts, we confirm that population growth is key to promoting cooperation.

## Introduction

Altruistic behaviors such as cooperation are ubiquitous at all system levels, ranging from microbial communities to human societies [1, 2], and they are crucial for the development and stability of real-life systems [3, 4]. Therefore, there has long been an acknowledgment of the importance of comprehending when individuals are willing to incur personal costs for group interests. The literature has uncovered several mechanisms to promote the evolution of cooperation, with population structures being one of the most significant ones [5]. Individuals and their mutual links are represented by nodes and edges, respectively, in networks.

An elementary assumption of most previous studies is that evolutionary dynamics take place in fully evolved populations [6–25], which leads to static spatial structures. For example, Allen et al [16] use coalescent theory to theoretically evaluate the propensity of arbitrary static networks to favor cooperation. Besides, Su et al [23] find out two simple network motifs to promote cooperation under asymmetric (but static) interactions. Numerous realistic instances, though, support that the dynamics in populations often coincide with the dynamics of population structures. One of the most common dynamics is population growth [26–31]. Specifically, current residents interact with their neighbors, while newcomers join the population and connect to the current residents to create a new networked population. The influence of network/population growth has been extensively studied theoretically and experimentally. For example, the Barabási-Albert model [26] uncovers the scale-freeness of real-world population structures with a simple growth rule, and some biological studies on infants demonstrate that the assembly of microbiome communities is advantageous for infant health [32, 33]. The prevalence of population growth and its massive impact in the real world leaves a question: How does population growth affect the evolution of cooperation?

Less research has been done on the impact of network growth in evolutionary dynamics than in network science [26–31], and just a few specific cases has been studied with numerical simulations. We mention two notable studies that investigate the evolution of cooperation in growing populations based on the framework of coevolutionary dynamics [34, 35]. They propose a modified attachment rule called an evolutionary preferential attachment, in which a newly added node has a higher probability of linking to existing nodes with greater payoffs. Simulations in these studies show that this mechanism can promote cooperation. Although numerical simulations are useful for rapid analysis in specific cases, a general growth rule, under which cooperation is favored by selection with population growth, is still missing.

Here we propose sequential temporal networks to model growing networked populations with the perspective of temporal networks. In our framework, populations can grow under arbitrary rules and structures. Contrary to coevolutionary dynamics [36–39], the growth process here is endogenous, which means that the growth is unrelated to the strategy, payoff, or other kinds of the state of individuals. We provide a mathematical analysis to judge whether a sequential temporal network promotes cooperation over its corresponding static network under both neutral drift and weak selection. We apply this analysis to four synthetic and four empirical sequential temporal networks and find that the evolution of cooperation can be successfully promoted in these networks. We also propose a theoretical approximation to quantify the cooperation-promoting effect of large sequential temporal networks under weak selection with less calculating complexity.

## Models

We consider a growing population of which the structure changing is captured by *L* ordered undirected snapshots. Each snapshot is represented by a network, where each node is occupied by an individual, and each edge represents a pairwise interaction. Since the dynamics of the population structure is independent of the dynamics on the population, such an ordered series of snapshots is known as a temporal network T [40], which provides a lossless representation of a time-varying population structure. In particular, we call the temporal networks in this study sequential temporal networks since the population size is monotonically increasing snapshot by snapshot. The final snapshot is called the static counterpart *S* for T, and the size of S is denoted as *N* (also the size of the population when reaching the steady state).

For ease of presentation, we symbolize the structure of sequential temporal networks as follows. A sequential temporal network $T=\{G^{\left(1\right)},...,G^{\left(L\right)}\}$ is determined by a static network S of size *N* and a set of activation vectors $A=\{a^{\left(1\right)},...,a^{\left(L\right)}\}$, where $G^{\left(l\right)}$ represents the *l*th snapshot for T, S shows the spatial structure when population growth finishes, and **a**^(*l*)^ records the activity of nodes in the *l*th snapshot. The element **a**^(*l*)^ is a vector of length *N*, where $a_{i}^{\left(l\right)}=1$ if node *i* is active in the *l*th snapshot, $a_{i}^{\left(l\right)}=0$ otherwise. Edge (*i*, *j*) exists in $G^{\left(l\right)}$ when (*i*, *j*) exists in S and individuals *i* and *j* are active in $G^{\left(l\right)}$.

Furthermore, we define a partial ordering ≼ on $R^{N}$ to illustrate the meaning of ‘sequential’. The relation $x={\left(x_{i}\right)}_{i=1}^{N}≼y={\left(y_{i}\right)}_{i=1}^{N}$ holds when *x*_*i*_ ≤ *y*_*i*_ for all 1 ≤ *i* ≤ *N*. Following the definition of sequential temporal networks, the relation $a^{\left(l_{1}\right)}≼a^{\left(l_{2}\right)}$ holds for all 1 ≤ *l*_1_ ≤ *l*_2_ ≤ *L* and **a**^(*L*)^ = (1, …, 1)^T^ (i.e. $G^{\left(L\right)}=S$).

In each snapshot, every individual chooses to cooperate or defect with all his neighbors. A cooperator pays cost *c* > 0 for each neighbor to receive benefit *b*, while a defector pays no cost and provides no benefit. This donation game is widely used when studying pairwise interactions [16, 21]. Traditionally, benefit *b* is set to be larger than 0, which means cooperation is prosocial behavior. But in some cases, selection favors cooperation only when *b* < 0 [16, 18]. Therefore, we set *b* ∈ (−∞, +∞) for a complete study of the evolution of cooperation.

The state of individual *i* is denoted by *x*_*i*_, where *x*_*i*_ = 1 (*x*_*i*_ = 0) suggests cooperation (defection). In each generation, individual *i* receives an edge-weighted average payoff, given by $u_{i}=-cx_{i}+b\sum_{j=1}^{N}p_{ij}^{\left(1\right)}x_{j}$, where $p_{ij}^{\left(1\right)}=w_{ij}/\sum_{k=1}^{N}w_{ik}$ represents the probability of a one-step random walk from *i* to *j*, and *w*_*ij*_ is the weight of edge (*i*, *j*), satisfying *w*_*ii*_ = 0 for any individual *i*, *w*_*ij*_ = 1 if a link exists between *i* and *j*, otherwise *w*_*ij*_ = 0, and *w*_*ij*_ = *w*_*ji*_ for any edge (*i*, *j*). The fitness of individual *i* positively depends on his payoff, measured by *F*_*i*_ = 1 + *δu*_*i*_. The parameter *δ* ≥ 0 describes the intensity of natural selection [9]. The regimes *δ* = 0 (*δ* ≪ 1) corresponds to neutral drift (weak selection) [41]. Each variable under neutral drift and weak selection is labeled with a superscript ^∘^ and *, respectively.

At the end of each generation, a random individual *i* is selected to update his strategy by imitating the strategy of one of *i*’s neighbors *j* with a probability proportional to *j*’s fitness *F*_*j*_ and the edge weight between *i* and *j*, given by

$$\begin{array}{c} e_{ji}=\frac{1}{N}\frac{F_{j}w_{ij}}{\sum_{k=1}^{N}F_{k}w_{ik}}. \end{array}$$ (1)

This update rule is well-known as death-birth updating, which is widely used in the field of evolution. Besides, we also analyze other update rules—pairwise-comparison updating and imitation updating in the S1 Text. Since there is no mutation in our model, after sufficient evolution, the state of populations will reach an all-cooperator state **C** or an all-defector state **D**, called an absorbing (equilibrium) state.

**Fig 1:**
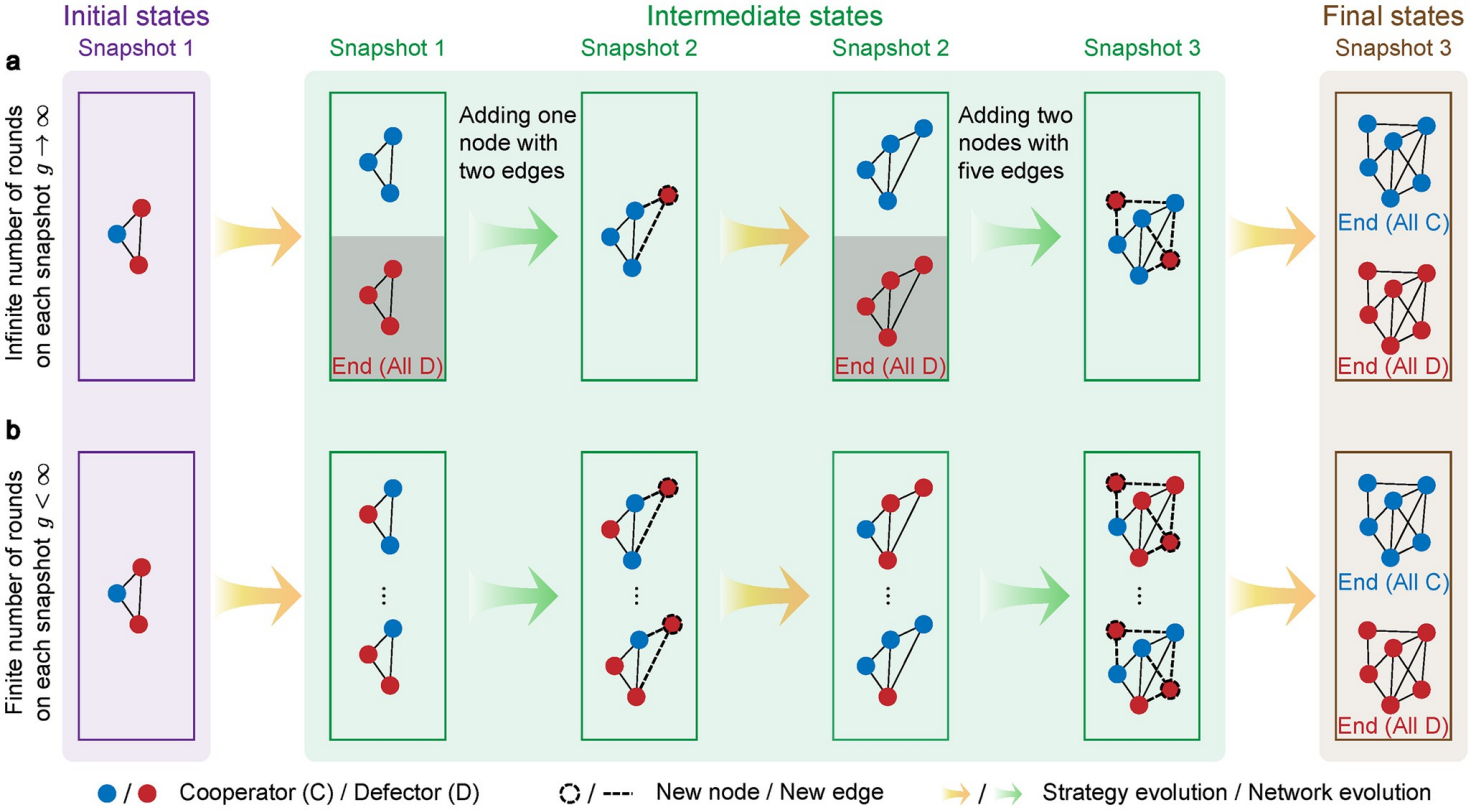
Illustration of two evolutionary processes on sequential temporal networks. We consider a sequential temporal network with three snapshots as an example. First, a random individual is selected to be a cooperator (C, blue) in the population full of defectors (D, red). **a**, In each snapshot, the evolutionary dynamics is sufficient so that the population reaches one of two homogeneous (absorbing) states: all-cooperator and all-defector state. Then, new defectors with links enter the population and continue the evolution. **b**, In each intermediate snapshot, the evolution proceeds finite rounds *g* before switching to the next. In this case, the state of the population may not be absorbed when the population structure changes. When *g* rises to infinity, this process is identical to the first process. In both processes, the evolution ends when the growth stops and the state is homogeneous.

We consider two classes of evolutionary processes on sequential temporal networks (Fig 1). In the first process, the state of populations reaches one of the absorbing states before new defector(s) enter the population (Fig 1a), which means that the evolutionary dynamics in each snapshot is sufficient and much faster than the network evolution. In the second process, the timescale between the evolutionary dynamics and the network evolution is controlled by a parameter *g* < ∞, which captures the number of generations in each snapshot (Fig 1b).

## Results

### General rule for the promotion of cooperation

We use the probability of reaching state **C** in a network, namely the fixation probability for cooperation [16, 18, 19, 21], to quantify the cooperation-promoting effect of the network. For a sequential temporal network, the fixation probability is then defined as the possibility of being **C** in the final snapshot. The value of the fixation probability is related to the initialization of the population state. In this study, we will use two types of initialization, uniform initialization *μ* (i.e. a random individual is chosen to be a cooperator in a population full of defectors) and the initialization of a specific configuration ***ξ*** = (*ξ*_1_, …, *ξ*_*N*_)^T^, where *ξ*_*i*_ = 1 if individual *i* is a cooperator, otherwise *ξ*_*i*_ = 0. We use the uniform initialization for both sequential temporal networks and their corresponding static networks, which means an individual is randomly chosen to be a cooperator in the first snapshot of sequential temporal networks and in the static networks, respectively. The respective fixation probabilities are denoted as $\rho_{T}^{\mu }$ and $\rho_{S}^{\mu }$.

We claim that a sequential temporal network T promotes the evolution of cooperation relative to its static counterpart S if:

$$\begin{array}{c} \rho_{T}^{\mu }>\rho_{S}^{\mu }. \end{array}$$ (2)

Eq (2) indicates that the probability of a single cooperator eventually taking over the population on T is higher than that on S. In this study, we focus on weak selection where $\rho_{T}^{\mu }$ becomes

$$\begin{array}{c} {\left(\rho_{T}^{\mu }\right)}^{*}={\left(\rho_{T}^{\mu }\right)}^{\circ }+\delta \frac{\partial \rho_{T}^{\mu }}{\partial \delta }{|}_{\delta =0}+O\left(\delta^{2}\right), \end{array}$$ (3)

and so does $\rho_{S}^{\mu }$.

We first analyze the first evolutionary process where the evolution is sufficient in each snapshot. In this case, the fixation for cooperation in T requires the fixation for cooperation in each snapshot of T, so the fixation probability for T is given by

$$\begin{array}{c} \rho_{T}^{\mu }=\rho_{G^{\left(1\right)}}^{\mu }\;\prod_{l=2}^{L}\;\rho_{G^{\left(l\right)}}^{a^{\left(l-1\right)}}, \end{array}$$ (4)

where $\rho_{G^{\left(l\right)}}^{a^{\left(l-1\right)}}$ represents the fixation probability for snapshot $G^{\left(l\right)}$ when the initialization is **a**^(*l*−1)^.

By Eq (3), we find that if ${\left(\rho_{T}^{\mu }\right)}^{\circ }>{\left(\rho_{S}^{\mu }\right)}^{\circ }$ holds, ${\left(\rho_{T}^{\mu }\right)}^{*}>{\left(\rho_{S}^{\mu }\right)}^{*}$ also holds. So we begin with analyzing Eq (2) under neutral drift. We first consider a simple case of unweighted sequential temporal networks of length *L* = 2. Besides, we assume that there are no interconnected edges among new nodes in the second snapshot. In this case, we have ${\left(\rho_{T}^{\mu }\right)}^{\circ }={\left(\rho_{G^{\left(1\right)}}^{\mu }\right)}^{\circ }{\left(\rho_{G^{\left(2\right)}}^{a^{\left(1\right)}}\right)}^{\circ }$. We assume the number of nodes and the average degree of $G^{\left(1\right)}$ are *m* and *k*_1_, and the increment of nodes and edges for $G^{\left(2\right)}$ is Δ*m* and Δ*K*. Using the technique proposed by McAvoy & Allen [19], we have

$$\begin{array}{c} {\left(\rho_{T}^{\mu }\right)}^{\circ }>{\left(\rho_{S}^{\mu }\right)}^{\circ }\Leftrightarrow \frac{1}{m}\frac{mk_{1}+\Delta K}{mk_{1}+2\Delta K}>\frac{1}{m+\Delta m}, \end{array}$$ (5)

which is equivalent to the following condition:

$$\begin{array}{c} \Delta m\geq m, \end{array}$$ (6a)

or

$$\begin{array}{c} \Delta m<m\;\text{and}\;\frac{\Delta K}{\Delta m}<\frac{mk_{1}}{m-\Delta m}. \end{array}$$ (6b)

We refer to S1 Text Section 3.1 for the detailed derivations of Eqs (5) and (6). In particular, when Δ*m* ≪ *m*, Eq (6b) degenerates to a simpler form,

$$\begin{array}{c} \Delta m<m\;\text{and}\;\frac{\Delta K}{\Delta m}<k_{1}. \end{array}$$ (7)

**Fig 2:**
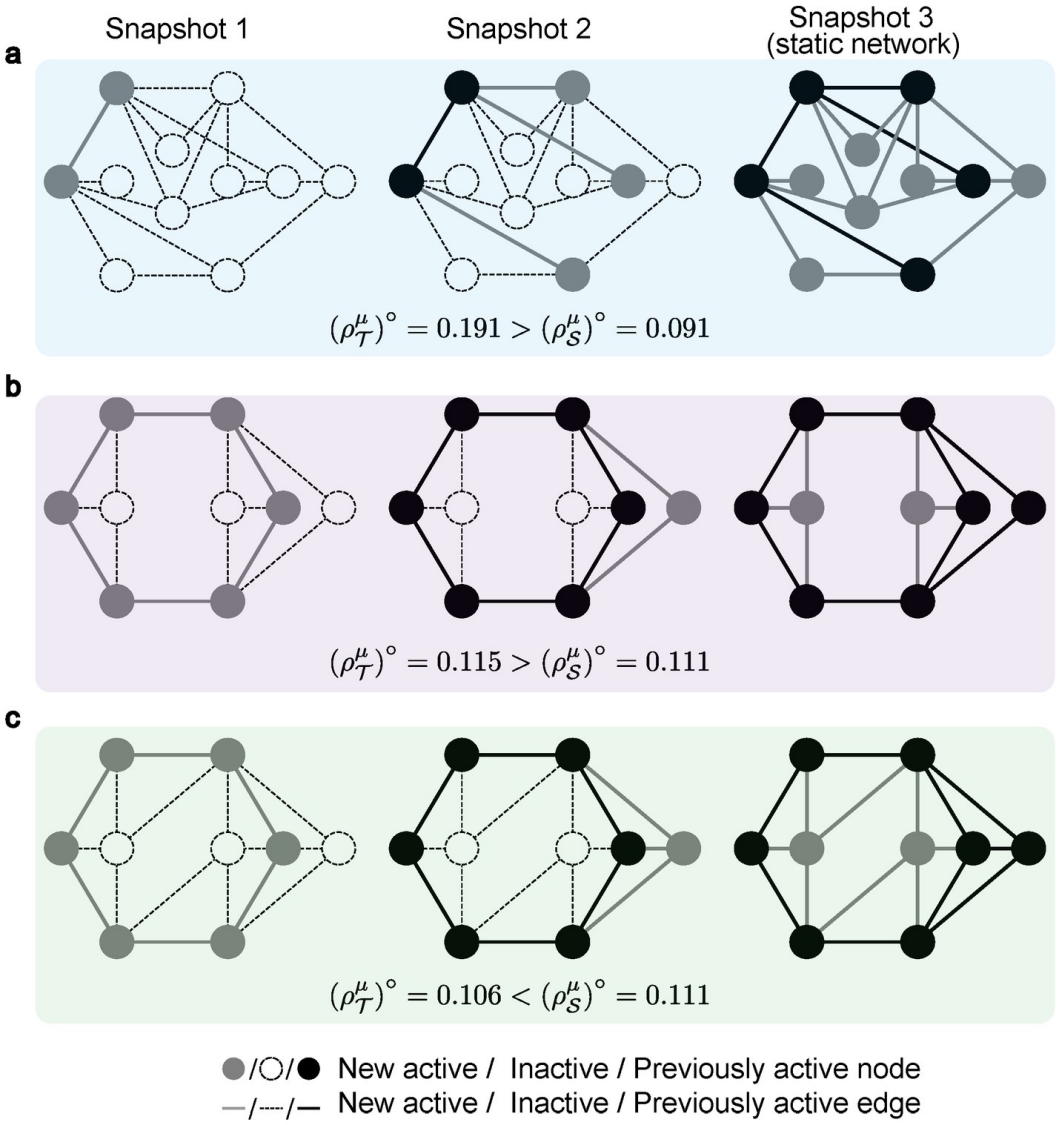
Fixation probabilities of sequential temporal networks and static networks. We present three examples for illustrating the analytical result in Eq (6). The number of nodes in the first and second snapshots is denoted as *m*_1_ and *m*_2_, and the increment of nodes (edges) in the second and third snapshots is denoted as Δ*m*_1_ (Δ*K*_1_) and Δ*m*_2_ (Δ*K*_2_). **a**, The number of nodes grows exponentially, fulfilling Eq (6a) (i.e. Δ*m*_1_ = 3 > *m*_1_ = 2 and Δ*m*_2_ = 6 > *m*_2_ = 5). Then the fixation probability of sequential temporal network, $\rho_{T}^{\circ }=0.191$, is greater than that of its static counterpart, $\rho_{S}^{\circ }=0.091$. **b**, The increase in the number of nodes and edges fulfills Eq (6b) (i.e. Δ*m*_1_ = 1 < *m*_1_ = 6, Δ*K*_1_ = 2 < 2.4 and Δ*m*_2_ = 2 < *m*_2_ = 7, Δ*K*_2_ = 6 < 6.4). As a result, the fixation probability is still higher in the sequential temporal network ($\rho_{T}^{\circ }=0.115>\rho_{S}^{\circ }=0.111$). **c**, When each pair of adjacent snapshots does not satisfy Eqs (6a) and (6b) (i.e. Δ*m*_1_ = 1 < *m*_1_ = 6, Δ*K*_1_ = 3 > 2.4 and Δ*m*_2_ = 2 < *m*_2_ = 7, Δ*K*_2_ = 8 > 7.2), the fixation probability becomes greater in the static network ($\rho_{T}^{\circ }=0.106<\rho_{S}^{\circ }=0.111$).

When *L* is greater than 2, it is straightforward to derive a sufficient condition for Eq (2) under neutral drift that each pair of adjacent snapshots satisfies Eqs (6a) or (6b). Fig 2 presents three examples to demonstrate our conclusion. In Fig 2a (Fig 2b), all pairs of adjacent snapshots satisfy Eq (6a) (Eq (6b)), so the sequential temporal network is more favorable for cooperation. However, in Fig 2c, each pair of adjacent snapshots violates Eqs (6a) and (6b), which leads to a lower possibility for cooperation in the sequential temporal network. Similar to Eq (5), we can also derive a necessary and sufficient condition for *L* ≥ 3 (see Methods and S1 Text. Section 3.1).

We provide an intuition of why population growth can promote cooperation under Eq (6). The main idea is to split the evolution into two (or more) stages to favor the fixation of cooperation. One way is to allow cooperation to first evolve in a relatively small population size (lower than *N*/2, Eq (6a)) before entering the subsequent snapshot. On one hand, cooperation spreads easily among a small population. On the other hand, once cooperators take over the population in the first snapshot, there is more than one cooperator at the beginning of the second snapshot. The combination of these two factors leads to an increased probability of cooperation relative to the static counterpart. However, when the initial population size is larger than *N*/2 (Eqs (6b) and (7)), it is less likely for cooperation to fixate in the first snapshot. To gain more advantages on fixation, we find that newly added nodes (i.e. defectors) can not be hubs in the second snapshot. In other words, the number of new edges should be upper-bounded, such that the average influence of cooperators is more significant than that of defectors.

When $\rho_{T}^{\mu }$ and $\rho_{S}^{\mu }$ are identical under neutral drift, we turn to compare the first-order term of them, that is,

$$\begin{array}{c} {\left(\rho_{T}^{\mu }\right)}^{*}>{\left(\rho_{S}^{\mu }\right)}^{*}\Leftrightarrow \frac{\partial \rho_{T}^{\mu }}{\partial \delta }{|}_{\delta =0}>\frac{\partial \rho_{S}^{\mu }}{\partial \delta }{|}_{\delta =0}. \end{array}$$ (8)

In S1 Text Section 3.2, we derive an analytical condition for Eq (8), and the complexity of verifying the condition is upper bounded by solving a system of linear equations of size *O*(*LN*^2^), which means the calculation cost is huge when *N* or *L* are large. To reduce the computational complexity, we develop a mean-field approximation method [42] for efficiently comparing the first-order term of $\rho_{T}^{\mu }$ and $\rho_{S}^{\mu }$ by avoiding solving the linear equations. For *L* = 2, the analytical condition under the mean-field approximation is given by

$$\begin{array}{c} {\left(\rho_{G^{\left(1\right)}}^{\mu }\right)}^{\circ }(-cC_{G^{\left(2\right)}}^{a^{\left(1\right)}}+bB_{G^{\left(2\right)}}^{a^{\left(1\right)}})+{\left(\rho_{G^{\left(2\right)}}^{a^{\left(1\right)}}\right)}^{\circ }(-cC_{G^{\left(1\right)}}^{\mu }+bB_{G^{\left(1\right)}}^{\mu })>(-cC_{G^{\left(2\right)}}^{\mu }+bB_{G^{\left(2\right)}}^{\mu }), \end{array}$$ (9)

where the quantity $-cC_{G^{\left(i\right)}}^{a^{\left(j\right)}}+bB_{G^{\left(i\right)}}^{a^{\left(j\right)}}$ shows the effect of the game model on cooperation spreading in snapshot $G^{\left(i\right)}$ under initialization **a**^(*j*)^ (see Methods for the explicit expression).

Eq (9) is a function of the benefit-to-cost ratio *b*/*c*, so the comparison between the cooperation-promoting effect of T and S not only depends on the population structure but also on the game. To avoid this situation, we introduce a useful index called the critical benefit-to-cost ratio (*b*/*c*)* in donation games [9, 16, 18, 21], which is only related to population structures. A lower positive critical ratio is interpreted as better for the emergence of cooperation. Meanwhile, a negative critical ratio is considered to favor spiteful behaviors. So a sequential temporal network T is more favourable for cooperation than the corresponding static network S, if ${\left(b/c\right)}_{S}^{*}>{\left(b/c\right)}_{T}^{*}>0$ or ${\left(b/c\right)}_{T}^{*}>0>{\left(b/c\right)}_{S}^{*}$. The first relation illustrates that the sequential temporal network decreases the required benefit-to-cost ratio to promote cooperation, and the second relation illustrates that the sequential temporal network can rescue cooperation when the corresponding static network favors spite [18, 43]. We also provide the exact and the mean-field expressions for ${\left(b/c\right)}_{T}^{*}$ and ${\left(b/c\right)}_{S}^{*}$ in Methods.

**Fig 3:**
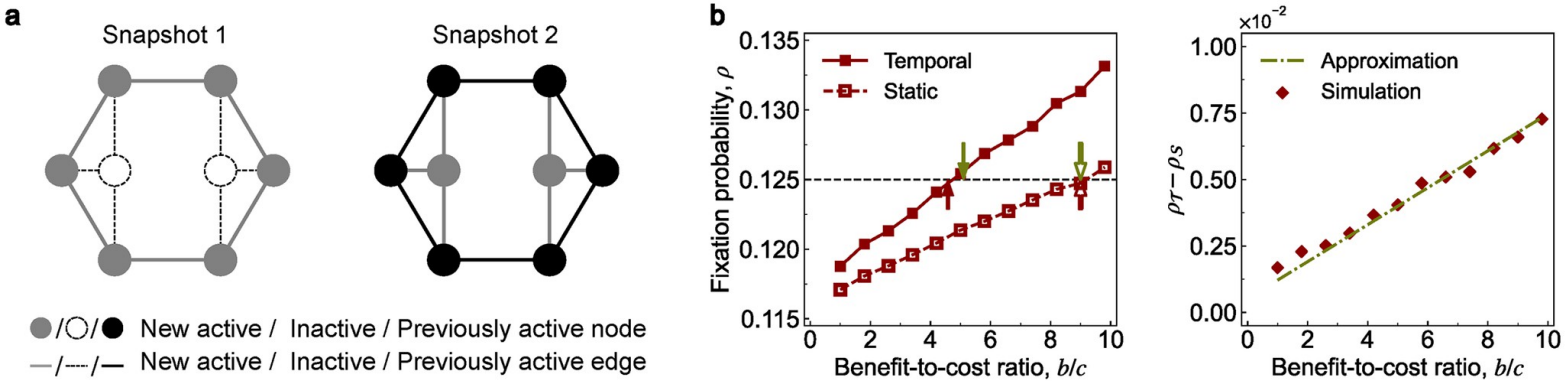
Fixation probabilities by the mean-field approximation. **a**, We consider a sequential temporal network with two snapshots. The fixation probabilities $\rho_{T}$ and $\rho_{S}$ are identical under neutral drift, i.e., $\rho_{T}^{\circ }=\rho_{S}^{\circ }=0.125$ (the black dashed line in **b**). **b**, We compare these two probabilities $\rho_{T}$ and $\rho_{S}$ under weak selection. We provide numerical simulations for the sequential temporal network (red solid line) and the static network (red dashed line) in the left panel. The probability $\rho_{T}$ is higher than the probability $\rho_{S}$ when the benefit-to-cost ratio *b*/*c* is larger than 1. We also calculate the difference between the two probabilities by our mean-field approximation (green dashed line), and the results can well predict the simulations (red diamonds). Furthermore, we calculate the critical benefit-to-cost ratio (*b*/*c*)* of the sequential temporal network (solid arrows) and the static network (dashed arrows) by the theoretical formula (red arrows) and approximation (green arrows). The sequential temporal network has a lower positive critical ratio, which shows a better potential to promote cooperation. Parameter values are *c* = 1, *δ* = 0.025.

Fig 3a provides an example to demonstrate the reliability of our method. The fixation probability of the sequential temporal network $\rho_{T}$ is the same as that of the corresponding static network $\rho_{S}$ under neutral drift but is greater under weak selection (Fig 3b). The difference between $\rho_{T}$ and $\rho_{S}$ can be fairly estimated by our mean-field approximation. In addition, the critical ratio ${\left(b/c\right)}_{T}^{*}=4.6$ is lower than the critical ratio ${\left(b/c\right)}_{S}^{*}=9.0$, which also reflects the advantage of the sequential temporal network in promoting cooperation. The mean-field approximation of ${\left(b/c\right)}_{T}^{*}$ and ${\left(b/c\right)}_{S}^{*}$ are 5.1 and 9.0 (green arrows in Fig 3b), respectively.

### Synthetic and empirical temporal networks

The sequential temporal networks discussed above are relatively short and small, but they nonetheless present a striking effect on the evolution of cooperation. We further study the evolutionary dynamics on more complex networks of size *N* = 100 and length *T* ≥ 95. We select four classes of static networks—square lattices with periodic boundaries [6], random regular graphs [44], Barabási-Albert scale-free networks [26], and scale-free networks with initial attractiveness [28]. The first two networks are homogeneous but have very different local structures, such as the clustering coefficient, and the last two are heterogeneous with different scaling laws. We use simple growth rules to form sequential temporal networks based on these static networks (see S1 Text Section 7.1, S1 and S2 Figs).

**Fig 4:**
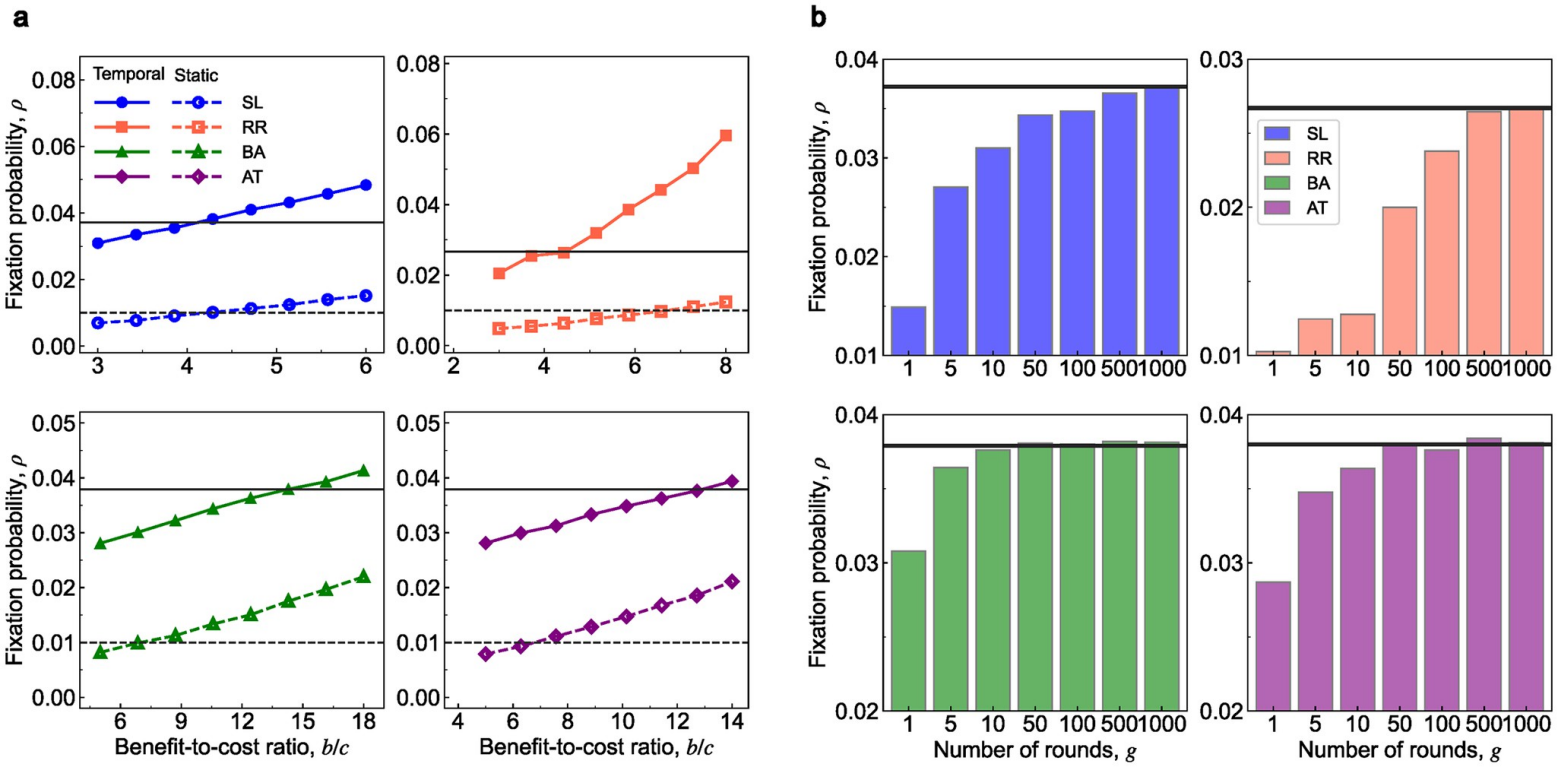
Evolution of cooperation on synthetic networks. We analyze four classes of underlying topologies of size *N* = 100: square lattices with periodic boundaries (abbreviated as SL), random regular graphs with average connectivity *k* = 6 (abbreviated as RR), Barabási-Albert scale-free networks with the linking number *m* = 3 (abbreviated as BA), and scale-free networks with initial attractiveness *a* = 50 and linking number *m* = 3 (abbreviated as AT). For each network, we obtain the numerical simulation of fixation probabilities by averaging over 10^6^ independent Monte Carlo simulations. **a**, For the first evolutionary process, the fixation probabilities of these sequential temporal networks (horizontal solid lines) are greater than those of their corresponding static counterparts (horizontal dashed lines) under neutral drift, and, therefore, are also larger under weak selection for a wide range of benefit-to-cost ratios *b*/*c*. **b**, We investigate the relationship between fixation probabilities and the number of rounds *g* under the second evolutionary process. For these sequential temporal networks, the fixation probabilities $\rho_{T}$ are monotonically increasing with respect to *g* under neutral drift. When *g* goes to infinite, the fixation probabilities converge to the fixation probabilities under the first evolutionary process (black solid lines). Parameter values are *c* = 1 and *δ* = 0.025.

We first analyze the networks under the first evolutionary process. Fig 4a shows the fixation probabilities of the static networks and their respective sequential temporal networks under neutral drift and weak selection. All these sequential temporal networks have higher fixation probabilities than static networks under neutral drift, which means that the sequential temporal networks promote the evolution of cooperation. Such promotion is robust for a wide scope of disturbances from games under weak selection. We can also calculate the fixation probabilities and the critical ratio of these networks with the mean-field approximation (see S3 and S4 Figs). The results show that the approximation is still reliable for these larger networks.

We turn to analyze the influence of the number of rounds *g* under the second evolutionary process. Specifically, we study the relationship between the fixation probability and the parameter *g* under neutral drift. Fig 4b shows the trend of the fixation probabilities of the sequential temporal networks when the parameter *g* rises. The fixation probabilities are monotonically increasing with respect to *g* and converge to the probabilities under the first evolutionary process as *g* rises to infinity. This conclusion also holds for a wide range of benefit-to-cost ratios *b*/*c* under weak selection (see S5 Fig). These results show that sequential temporal networks become more favorable for cooperation when the evolution on each snapshot is more sufficient. A straightforward explanation is that cooperators are more likely to aggregate into clusters in a smaller population, and long-term evolution fosters the emergence of such clusters. As a result, cooperators can occupy the whole small population by sufficient evolution, and then it is difficult for new defectors to invade the population.

In the Supporting Information, we demonstrate that the monotonicity with *g* is determined by the growth rule of sequential temporal networks. We provide a theoretical explanation under regular networks that the above monotonicity holds if the average degree of the front snapshot is larger than that of the back snapshot for any pair of adjacent snapshots. This condition is similar to Eqs (6b) and (7), and hence, it is highly possible for them to be satisfied at the same time.

Furthermore, we notice that the fixation probabilities with the parameter *g* = 1 of these sequential temporal networks (1.49 × 10^−2^, 1.03 × 10^−2^, 3.09 × 10^−2^, and 2.87 × 10^−2^) are all higher than the fixation probability of static networks (1 × 10^−2^). In the sense of Eq (2), it is not necessary to fully evolve on every snapshot to promote cooperation. Intuitively, the parameter *g* affects the expected time of reaching the all-cooperator state **C** (the conditional absorbing time of the state **C**). Increasing the duration time *g* improves the fixation probability but consumes a longer time to reach the ideal state **C**. This raises the question of whether there is a value of *g* to balance the cooperation-promoting effect and the conditional absorbing time. We find an expected tradeoff for these sequential temporal networks. When the duration time *g* equals 10, the fixation probability is higher, and the conditional absorbing time is lower than the static networks (see S6 Fig).

**Fig 5:**
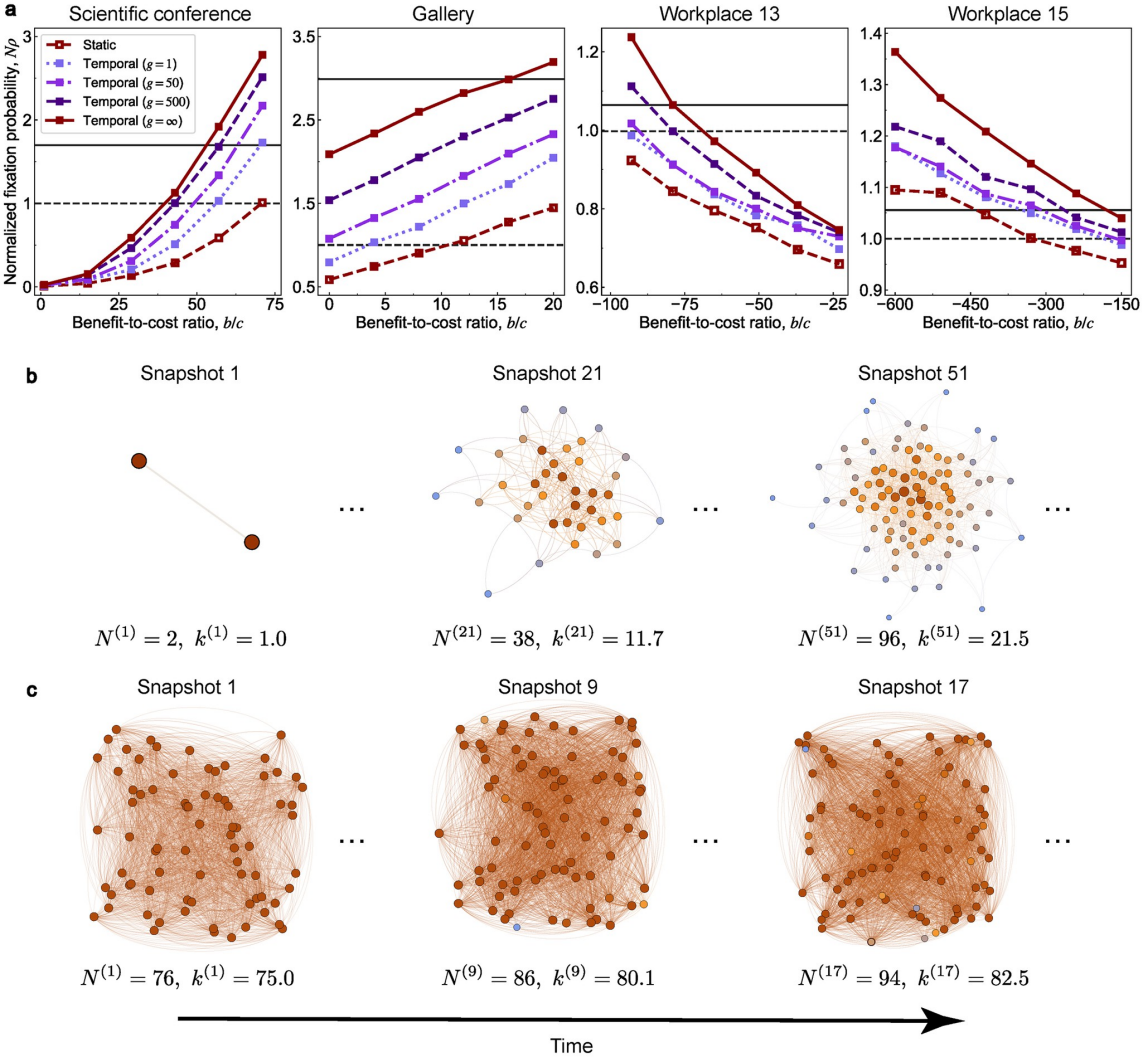
Evolution of cooperation in four empirical datasets. We analyze four empirical datasets from different social contexts: a scientific conference in Nice, France [45], the Science Gallery in Dublin, Ireland [46], a workplace in two different years in France [47]. **a**, the sequential temporal networks promote the evolution of cooperation for any number of rounds *g* in these datasets. The corresponding fixation probabilities are monotonically increasing with respect to the parameter *g*. Parameter values are *δ* = 0.025 for the first dataset, *δ* = 0.01 for the remaining datasets, and *c* = 1 for all datasets. **b**-**c**, We present the structure of the sequential temporal networks for the first (**b**) and third datasets (**c**). The number of nodes and the average degree at time *t* are denoted as *N*^(*t*)^ and *k*^(*t*)^, respectively.

We also investigate the evolutionary dynamics over four empirical datasets from SocioPatterns (http://www.sociopatterns.org). These datasets come from three social contexts: a scientific conference in France [45], a gallery in Ireland [46], and an office building in two years (2013 and 2015) in Frances [47]. Each dataset is formed by a set of triplets (*t*, *i*, *j*), which represent that individual *i* contacts with *j* at time *t*. We construct four empirical static networks and corresponding sequential temporal networks based on these triplets (see S1 Text section 7.2 for details). Some structural information about these networks is listed in S2 Table. Fig 5 shows the fixation probabilities of these networks under weak selection. All these sequential temporal networks facilitate the evolution of cooperation since they have higher fixation probabilities under neutral drift. In addition, even if the evolution is not sufficient (*g* < ∞), the promotion remains across a wide scale of benefit-to-cost ratios *b*/*c*. The monotonicity of fixation probabilities with respect to the parameter *g* also exists in these empirical datasets.

The fixation probabilities of the first two datasets have an opposite trend to the last two datasets. This trend is determined by the sign of the critical benefit-to-cost ratio (*b*/*c*)*. A positive (negative) critical ratio means the fixation probability is monotonically increasing (decreasing) with respect to the ratio *b*/*c*. We use the mean-field approximation to estimate the critical benefit-to-cost ratio of these networks (see S2 Table). Remarkably, this approximation can handle both positive and negative critical ratios with high accuracy. In general, the critical ratios of static networks and corresponding sequential temporal networks have an identical sign (see Figs 4 and 5).

## Discussion

Time-varying interactions are ubiquitous in real-world populations, and population growth is a key factor in this phenomenon. A large body of studies from different research fields pay attention to the cause and influence of growth, such as information spreading [48, 49], citation networks [50], and epidemic dynamics [51]. In this work, we study the evolutionary dynamics in growing populations modeled by sequential temporal networks. We derive a general growth rule under which sequential temporal networks promote the evolution of cooperation, and we find that many synthetic and empirical sequential temporal networks satisfy the rule. When the evolutionary process is divided into several stages (snapshots), cooperators can spread over an entire population step by step from a small-sized population to a large-sized population. At the same time, cooperators can gather as a cluster to effectively defend against new defectors. Both of these effects contribute to the emergence of cooperation.

Our theoretical analysis and numerical simulations have shown that the cooperation-promoting effect of sequential temporal networks originates from the entire sequence of temporal snapshots (i.e., the complete growth process), rather than how simple the initial population structure is. On the one hand, for the simplest initial population that consists of two nodes and a link, different growth processes can lead to completely different evolutionary outcomes (S7 Fig). On the other hand, even if the initial population is complicated and notoriously bad for cooperation, the corresponding temporal network can still promote cooperation (Fig 5c). In fact, one of the contributions of this study is to show how sequential temporal interactions get packaged together to determine overall evolutionary dynamics.

We have mentioned that the sequential temporal networks in this work should be contrasted with dynamical networks and coevolutionary dynamics, in which the temporality of population structures is caused by exogenous factors, such as individuals’ payoffs [34, 35] and strategies [36–39]. In other words, in coevolutionary dynamics, the dynamics of the network is established by the evolution of cooperation. However, just like the empirical datasets in Fig 5, topology switching is often independent of the dynamics in populations. This emphasizes the significance of studying the independent influence of temporality in the evolution of cooperation [52, 53]. In this work, we concentrate on the temporality caused by population growth. The rule we have proposed is only related to the increment of nodes and edges and can theoretically explain the simulations in the above two studies. The well-known evolutionary preferential attachment [34, 35] can be viewed as a typical case of our rule.

We describe the timescale of evolutionary dynamics and network evolution by the duration time of interactions (the number of rounds) *g* and have discussed two typical cases: sufficient evolution (*g* = ∞) and infinite-time evolution (*g* < ∞). We find that both cases have similar mechanisms to promote cooperation and fuller evolution is more favorable to promoting cooperation. However, evolution is inherently costly, as individual interactions and strategy updates may consume resources. Therefore, individuals prefer striking a balance between the potential of fostering cooperation and the consumption of evolutionary time. Intriguingly, we find an ideal duration time where cooperation is significantly promoted and the time is acceptable (S6 Fig). This explains why a finite communication period is more common in the real world.

Our analysis is based on neutral drift and weak selection. We only apply the rule under weak selection if the rule under neutral drift fails. In fact, the rule under neutral drift is available for most sequential temporal networks (S8 Fig), especially for complicated sequential temporal networks. This is meaningful because the computational complexity under neutral drift is much lower than under weak selection.

We also showed the generality of our rules by investigating a broad range of random graphs. Even for the simplest sequential temporal networks (length *L* = 2), more than 80% of them can promote cooperation (S8 Fig). Furthermore, the proportion increases monotonically to nearly 100% as the length of sequential temporal networks becomes longer. Meanwhile, we find that, in general, a longer sequential temporal network has a better cooperation-promoting effect (S9 Fig). Investigating a suitable length is a worthwhile direction for real-world applications.

We have focused on pairwise interactions, where individuals engage in two-player games. In addition, cooperation can also unfold in groups, such as in public goods games [11, 13, 14]. A natural extension is to consider higher-order interactions [54, 55] or group interactions [18, 56] in populations. In this case, several new individuals enter populations as a whole with internal interactions and may simultaneously play two-player games and multi-player games with different opponents. Therefore, while we uncover many striking properties of sequential temporal networks, a further understanding of population growth remains an important topic for various research fields.

## Methods

### Variants of the model

We show three variants of the model and the corresponding equivalent condition of Eq (2) under neutral drift. We refer to S1 Text for detailed mathematical derivations.

We first consider weighted sequential temporal networks of length *L* = 2. A weighted link means that more than (or less than) one interaction occurs per unit of time. In this case, we generalize the concept of average degree *k* to average node strength *w*. We assume the number of nodes and the average node strength of $G^{\left(1\right)}$ are *m* and *w*_1_, and the increment of nodes and edges for $G^{\left(2\right)}$ is Δ*m* and Δ*K*, the equivalent condition of Eq (2) is given by

$$\begin{array}{c} \Delta m\geq m, \end{array}$$ (10a)

or

$$\begin{array}{c} \Delta m<m\;\text{and}\;\frac{\Delta K}{\Delta m}<\frac{mw_{1}}{m-\Delta m}. \end{array}$$ (10b)

We then consider an unweighted sequential temporal network of length *L* = 2 with internal links among newly added nodes in the second snapshot. We assume that there are *g*_1_ internal edges nodes and *g*_2_ external edges in Δ*m* new nodes. A similar derivation leads to

$$\begin{array}{c} g_{1}\geq \frac{\Delta mk_{1}}{2},\;\Delta m>m,\;g_{2}>\frac{2mg_{1}-m\Delta mk_{1}}{\Delta m-m} \end{array}$$ (11a)

$$\begin{array}{c} g_{1}<\frac{\Delta mk_{1}}{2},\;\Delta m\geq m, \end{array}$$ (11b)

$$\begin{array}{c} g_{1}<\frac{\Delta mk_{1}}{2},\;\Delta m<m,\;g_{2}<\frac{m\Delta mk_{1}-2mg_{1}}{m-\Delta m}. \end{array}$$ (11c)

Finally, we provide the equivalent condition of Eq (2) for unweighted sequential temporal networks of arbitrary length *L*, given by

$$\begin{array}{c} \frac{1}{m_{1}}\prod_{l=2}^{L}\frac{m_{l-1}k_{l-1}+m_{l}k_{l}}{2m_{l}k_{l}}>\frac{1}{m_{L}k_{L}}, \end{array}$$ (12)

where *m*_*l*_ and *k*_*l*_ are the number of nodes and the average connectivity for the *l*th snapshot $G^{\left(l\right)}$, respectively.

### Mean-field approximation

We briefly summarize the mean-field approximation of the fixation probability and the critical benefit-to-cost ratio under DB updating. Detailed derivations and the results for other update rules can be found in S1 Text.

A static network S of size *N* is specified by a matrix ${\left(w_{ij}\right)}_{i,j=1}^{N}$, where *w*_*ij*_ is the weight of edge (*i*, *j*), satisfying *w*_*ij*_ = *w*_*ji*_, and *w*_*ij*_ > 0 if an edge exists between individuals *i* and *j*, *w*_*ij*_ = 0 otherwise. The node strength of node *i* is $w_{i}=\sum_{j=1}^{N}w_{ij}$, and the probability of a *n*-step random walk from node *i* to node *j* is denoted as $p_{ij}^{\left(n\right)}$. The reproductive value of node *i* is $\pi_{i}=w_{i}/\sum_{k=1}^{N}w_{k}$, which is the invariant distribution of random walks on S. For any vector **y** = (*y*_1_, …, *y*_*N*_)^T^ on S, we define the RV-weighted value $\hat{y}≔\sum_{i=1}^{N}\pi_{i}y_{i}$.

For an initial configuration ***ξ*** = (*ξ*_1_, …, *ξ*_*N*_)^T^, we let $t_{ij}=N\left(\hat{\xi }-\xi_{i}\xi_{j}\right)/2$ and $B_{0}=\sum_{i,j=1}^{N}\pi_{i}\pi_{j}t_{ij}$. The mean-field approximation of the fixation probabilities ${\left(\rho_{S}^{\xi }\right)}^{*}$ and ${\left(\rho_{S}^{\mu }\right)}^{*}$ is given by

$$\begin{array}{cc} {\left(\rho_{S}^{\xi }\right)}^{*} & \approx \hat{\xi }+\frac{\delta }{N}(-c\cdot C_{S}^{\xi }+b\cdot B_{S}^{\xi })+O\left(\delta^{2}\right)\\ & =\hat{\xi }+\frac{\delta }{N}(-c\gamma_{\left(2\right)}^{\xi }+b\left(\gamma_{\left(3\right)}^{\xi }-\gamma_{\left(1\right)}^{\xi }\right))+O\left(\delta^{2}\right)\\ & =\hat{\xi }+\frac{\delta }{N}(-c(\frac{B_{0}N\mu_{1}^{2}}{\mu_{2}}-\frac{1}{N\mu_{1}}\left(C_{0}+C_{1}\right))\\ & +\;b(\frac{B_{0}\Lambda \mu_{1}}{\mu_{2}}-\frac{1}{N\mu_{1}}\left(C_{1}+C_{2}\right)))+O\left(\delta^{2}\right),\\ {\left(\rho_{S}^{\mu }\right)}^{*} & \approx \frac{1}{N}+\frac{\delta }{N}(-c\cdot C_{S}^{\mu }+b\cdot B_{S}^{\mu })+O\left(\delta^{2}\right)\\ & =\frac{1}{N}+\frac{\delta }{N}(-c\gamma_{\left(2\right)}^{\mu }+b\left(\gamma_{\left(3\right)}^{\mu }-\gamma_{\left(1\right)}^{\mu }\right))+O\left(\delta^{2}\right)\\ & =\frac{1}{N}+\frac{\delta }{N}(-c(\frac{N\mu_{1}^{2}}{2\mu_{2}}-1)+b(\frac{\Lambda \mu_{1}}{2\mu_{2}}-1))+O\left(\delta^{2}\right), \end{array}$$ (13)

where

$$\begin{array}{cc} \gamma_{\left(1\right)}^{\xi } & =\frac{B_{0}N\mu_{1}^{2}}{\mu_{2}}-\frac{1}{N\mu_{1}}\sum_{i=1}^{N}w_{i}t_{ii},\\ \gamma_{\left(2\right)}^{\xi } & =\frac{B_{0}N\mu_{1}^{2}}{\mu_{2}}-\frac{1}{N\mu_{1}}(\sum_{i,j=1}^{N}w_{i}\left(p_{ij}^{\left(0\right)}+p_{ij}^{\left(1\right)}\right)t_{ij}),\\ \gamma_{\left(3\right)}^{\xi } & =\frac{B_{0}N\mu_{1}^{2}}{\mu_{2}}+\frac{B_{0}\mu_{1}\sum_{i=1}^{N}w_{i}p_{ii}^{\left(2\right)}}{\mu_{2}}-\frac{1}{N\mu_{1}}(\sum_{i,j=1}^{N}w_{i}\left(p_{ij}^{\left(0\right)}+p_{ij}^{\left(1\right)}+p_{ij}^{\left(2\right)}\right)t_{ij}),\\ \gamma_{\left(1\right)}^{\mu } & =\frac{N\mu_{1}^{2}}{2\mu_{2}}-\frac{1}{2},\\ \gamma_{\left(2\right)}^{\mu } & =\frac{N\mu_{1}^{2}}{2\mu_{2}}-1,\\ \gamma_{\left(3\right)}^{\mu } & =\frac{N\mu_{1}^{2}}{2\mu_{2}}+\frac{\sum_{i=1}^{N}w_{i}p_{ii}^{\left(2\right)}\mu_{1}}{2\mu_{2}}-\frac{3}{2}. \end{array}$$ (14)

The quantities $\mu_{1}=\sum_{i=1}^{N}w_{i}/N$ and $\mu_{2}=\sum_{i=1}^{N}w_{i}^{2}/N$ are the first and second moments of the node strength distribution, $C_{k}=\sum_{i,j=1}^{N}w_{i}p_{ij}^{\left(k\right)}t_{ij}$ for *k* = 0, 1, 2, and $\Lambda =\sum_{i=1}^{N}w_{i}p_{ii}^{\left(2\right)}$. All these quantities can be calculated directly by structural information of the network, instead of solving linear systems. Applying Eqs (4) and (13), we can obtain the mean-field approximation of ${\left(\rho_{T}^{\mu }\right)}^{*}$ under the first evolutionary process.

The approximation of the critical benefit-to-cost ratio of S under initial configuration ***ξ*** is given by

$$\begin{array}{c} {\left(\frac{b}{c}\right)}_{\xi }^{*}\approx \frac{B_{0}N^{2}\mu_{1}^{2}-\mu_{2}\left(C_{0}+C_{1}\right)}{B_{0}N\Lambda \mu_{1}-\mu_{2}\left(C_{1}+C_{2}\right)}, \end{array}$$ (15)

and the approximation under uniform initialization is given by

$$\begin{array}{c} {\left(\frac{b}{c}\right)}_{\mu }^{*}\approx \frac{N\mu_{1}^{2}-2\mu_{2}}{\Lambda \mu_{1}-2\mu_{2}}. \end{array}$$ (16)

For a sequential temporal network $T=\left(S,A\right)=\left\{G^{\left(1\right)},...,G^{\left(L\right)}\right\}$, let $A_{1}={\left(\rho_{G^{\left(1\right)}}^{\mu }\right)}^{\circ }$, $A_{i}={\left(\rho_{G^{\left(i\right)}}^{a^{\left(i-1\right)}}\right)}^{\circ }$ (*i* = 2, …, *L*) and ${\tilde{A}}_{i}=\prod_{j\neq i}\;A_{j}$. The approximate critical value is

$$\begin{array}{c} {\left(\frac{b}{c}\right)}_{T}^{*}=\frac{{\tilde{A}}_{1}(\frac{\gamma_{\left(2\right)}^{\mu }}{N}){|}_{G^{\left(1\right)}}+(\sum_{i=2}^{L}{\tilde{A}}_{i}(\frac{\gamma_{\left(2\right)}^{a^{\left(i-1\right)}}}{N}){|}_{G^{\left(i\right)}})}{{\tilde{A}}_{1}(\frac{\gamma_{\left(3\right)}^{\mu }-\gamma_{\left(1\right)}^{\mu }}{N}){|}_{G^{\left(1\right)}}+(\sum_{i=2}^{L}{\tilde{A}}_{i}(\frac{\gamma_{\left(3\right)}^{a^{\left(i-1\right)}}-\gamma_{\left(1\right)}^{a^{\left(i-1\right)}}}{N}){|}_{G^{\left(i\right)}})}, \end{array}$$ (17)

where the notation ${\cdot |}_{G^{\left(i\right)}}$ indicates that the value is taken under the network $G^{\left(i\right)}$.

## Supporting information

S1 Text Theoretical deviations and construction algorithms. Detailed calculations of fixation probabilities and critical benefit-to-cost ratios under different updating rules. Derivations of the mean-field approximation under different updating rules. Algorithms for constructing sequential temporal networks.
(PDF)

S1 Fig Construction of the sequential temporal network on square lattices. A schematic illustration of constructing a sequential temporal network on a square lattice of size *N* = 16. First, four nodes in the middle of a square lattice are set to be active. Then, at each time step, we activate one node in turn clockwise along the circle. The construction finishes when all nodes are active.
(PDF)

S2 Fig Construction of the sequential temporal network on Barabási-Albert scale-free networks. A schematic illustration of constructing a sequential temporal network on a Barabási-Albert scale-free network of size *N* = 12 and linking number *m* = 2. First, we activate *m*_0_ nodes to form an initial snapshot. Due to the growth and preferential attachment, at each time step, a new node enters the network and connects to *m* old nodes. At this point, the new node is active. The length of sequential temporal networks is equal to the number of new nodes plus one.
(PDF)

S3 Fig Fixation probabilities by simulations and mean-field approximations. Comparisons between the fixation probabilities for cooperation obtained by numerical simulations (dashed lines) or the mean-field approximation (dots). The fixation probabilities are calculated under three setups: static networks with uniform initialization and a given initial configuration, and sequential temporal networks. Network structures and parameter values are the same as in Fig 4 in the main text.
(PDF)

S4 Fig Critical benefit-to-cost ratio by simulations and mean-field approximations. We consider the same network structure and the same initialization as S3 Fig. The results show that the mean-field approximation is also accurate for estimating the critical ratio of static networks and sequential temporal networks.
(PDF)

S5 Fig Fixation probability for finite *g* under weak selection. We consider the same network topologies as those in Fig 4 in the main text. The results show that the monotonicity of the fixation probability with respect to generation time *g* holds under weak selection for a wide range of benefit-to-cost ratio *b*/*c*. Parameter values are *c* = 1, *δ* = 0.015.
(PDF)

S6 Fig Absorbing times under a different number of rounds. The conditional absorbing time and unconditional absorbing time of static networks and sequential temporal networks. We first focus on the conditional absorbing time of reaching the all-cooperator state **C** (first row). We find that when the number of rounds *g* = 10, the sequential temporal networks can both promote the evolution of cooperation and have lower absorbing time than static networks (light solid line). We also present the result of the conditional absorbing time of reaching the all-defector state **D** (second row) and the unconditional absorbing time (third row). Network structures and parameter values are the same as in Fig 4 in the main text.
(PDF)

S7 Fig Growth process determines the cooperation-promoting effect of sequential temporal networks. We consider two sequential temporal networks with the same initial and final snapshots. a, The increment of nodes for each pair of successive snapshots is one. In this case, the fixation probability of the sequential temporal network under neutral drift is higher than that of the static counterpart. b, The length of the sequential temporal network is 2, which means that there is no intermediate snapshot between the initial and final snapshots. In this case, the fixation probability of the sequential temporal network under neutral drift is lower than that of the static counterpart.
(PDF)

S8 FigFixation probabilities for 30000 random graphs.We analyze three classes of random graphs as static networks: Erdös-Rényi networks, Watts-Strogatz small-world networks with rewiring probability 0.3, and Barabási-Albert scale-free networks. For each class, we sample 10^4^ graphs of size *N* and average degree *k*, where *N* is randomly selected from [20, 50] and *k* is randomly selected form [4, *N*/2]. For each static network, we apply Algorithm 1 in S1 Text to construct a sequential temporal network with length *L*_*tol*_. Then, we use the sequential temporal network to generate new sequential temporal networks with length *L* ≤ *L*_*tol*_, where the first and the final snapshots of the new networks are the same as the original one. The black lines show the fixation probabilities of static networks under neutral drift (${\left(\rho_{S}^{\mu }\right)}^{\circ }=1/N$). The proportion of sequential temporal networks with a higher fixation probability (${\left(\rho_{T}^{\mu }\right)}^{\circ }>{\left(\rho_{S}^{\mu }\right)}^{\circ }$) is monotonically increasing with *L*, rising from 83% when *L* = 2, to 87% when *L* = 5, and to 96% when *L* = 10, and to nearly 100% when *L* = *L*_*tol*_. The number of sequential temporal networks with the same fixation probability ($\rho_{T}^{\circ }=1/N$) is monotonically decreasing with *L*, from 662 when *L* = 2, to 1 when *L* = 5, and to 0 when *L* = 10 and *L* = *L*_*tol*_.(PDF)

S9 Fig Effect of network length. We analyze the same networks as in S8 Fig. We calculate the fixation probabilities for sequential temporal networks with different lengths. In general, the sequential temporal networks with longer lengths have higher fixation probabilities.
(PDF)

S1 TableStructural information of synthetic networks.We analyze the same networks as in Fig 4. *N* is the number of nodes in static networks, *L* is the length of sequential temporal networks, and *k* is the average degree of static networks. ${\left(b/c\right)}_{T}^{*}$ and ${\left(b/c\right)}_{S}^{*}$ are the simulation-based critical benefit-to-cost ratios of sequential temporal networks and static networks, respectively. Approx. ${\left(b/c\right)}_{T}^{*}$ and Approx. ${\left(b/c\right)}_{S}^{*}$ are the corresponding approximate values with the mean-field approximation.(PDF)

S2 Table Structural information of empirical networks. We analyze the same networks as in Fig 5. The implication of parameters is the same as S1 Table.
(PDF)

## References

[1] Hofbauer J, Sigmund K. Evolutionary Games and Population Dynamics. Cambridge University Press; 1998.

[2] Axelrod R, Hamilton WD. The evolution of cooperation. Science. 1981;211(4489):1390–1396. doi: 10.1126/science.7466396 7466396

[3] Keohane RO, Victor DG. Cooperation and discord in global climate policy. Nat Clim Change. 2016;6(6):570–575. doi: 10.1038/nclimate2937

[4] Block P, Hoffman M, Raabe IJ, Dowd JB, Rahal C, Kashyap R, et al. Social network-based distancing strategies to flatten the COVID-19 curve in a post-lockdown world. Nat Hum Behav. 2020;4(6):588–596. doi: 10.1038/s41562-020-0898-6 32499576

[5] Nowak MA. Five rules for the evolution of cooperation. Science. 2006;314(5805):1560–1563. doi: 10.1126/science.1133755 17158317 PMC3279745

[6] Nowak MA, May RM. Evolutionary games and spatial chaos. Nature. 1992;359(6398):826–829. doi: 10.1038/359826a0

[7] Szabó G, Tőke C. Evolutionary prisoner’s dilemma game on a square lattice. Phys Rev E. 1998;58(1):69. doi: 10.1103/PhysRevE.58.69

[8] Hauert C, Doebeli M. Spatial structure often inhibits the evolution of cooperation in the snowdrift game. Nature. 2004;428(6983):643–646. doi: 10.1038/nature02360 15074318

[9] Nowak MA, Sasaki A, Taylor C, Fudenberg D. Emergence of cooperation and evolutionary stability in finite populations. Nature. 2004;428(6983):646–650. doi: 10.1038/nature02414 15071593

[10] Santos FC, Pacheco JM. Scale-free networks provide a unifying framework for the emergence of cooperation. Phys Rev Lett. 2005;95(9):098104. doi: 10.1103/PhysRevLett.95.098104 16197256

[11] Santos FC, Santos MD, Pacheco JM. Social diversity promotes the emergence of cooperation in public goods games. Nature. 2008;454(7201):213–216. doi: 10.1038/nature06940 18615084

[12] Tarnita CE, Ohtsuki H, Antal T, Fu F, Nowak MA. Strategy selection in structured populations. J Theor Biol. 2009;259(3):570–581. doi: 10.1016/j.jtbi.2009.03.035 19358858 PMC2710410

[13] Li A, Wu B, Wang L. Cooperation with both synergistic and local interactions can be worse than each alone. Sci Rep. 2014;4(1):5536. doi: 10.1038/srep05536 24985887 PMC4078301

[14] Li A, Broom M, Du J, Wang L. Evolutionary dynamics of general group interactions in structured populations. Phys Rev E. 2016;93(2):022407. doi: 10.1103/PhysRevE.93.022407 26986362

[15] Allen B, Nowak MA. Games on graphs. EMS Surv Math Sci. 2014;1(1):113–151. doi: 10.4171/EMSS/3

[16] Allen B, Lippner G, Chen YT, Fotouhi B, Momeni N, Yau ST, et al. Evolutionary dynamics on any population structure. Nature. 2017;544(7649):227–230. doi: 10.1038/nature21723 28355181

[17] Allen B, Lippner G, Nowak MA. Evolutionary games on isothermal graphs. Nat Commun. 2019;10(1):5107. doi: 10.1038/s41467-019-13006-7 31704922 PMC6841731

[18] McAvoy A, Allen B, Nowak MA. Social goods dilemmas in heterogeneous societies. Nat Hum Behav. 2020;4(8):819–831. doi: 10.1038/s41562-020-0881-2 32451481

[19] McAvoy A, Allen B. Fixation probabilities in evolutionary dynamics under weak selection. J Math Biol. 2021;82(3):14. doi: 10.1007/s00285-021-01568-4 33534054

[20] Zhou L, Wu B, Du J, Wang L. Aspiration dynamics generate robust predictions in heterogeneous populations. Nat Commun. 2021;12(1):3250. doi: 10.1038/s41467-021-23548-4 34059670 PMC8166829

[21] Ohtsuki H, Hauert C, Lieberman E, Nowak MA. A simple rule for the evolution of cooperation on graphs and social networks. Nature. 2006;441(7092):502–505. doi: 10.1038/nature04605 16724065 PMC2430087

[22] Su Q, McAvoy A, Wang L, Nowak MA. Evolutionary dynamics with game transitions. Proc Natl Acad Sci U S A. 2019;116(51):25398–25404. doi: 10.1073/pnas.1908936116 31772008 PMC6926053

[23] Su Q, Allen B, Plotkin JB. Evolution of cooperation with asymmetric social interactions. Proc Natl Acad Sci U S A. 2022;119(1):e2113468118. doi: 10.1073/pnas.2113468118 34983850 PMC8740725

[24] Peña J, Wu B, Arranz J, Traulsen A. Evolutionary games of multiplayer cooperation on graphs. PLoS Comput Biol. 2016;12(8):e1005059. doi: 10.1371/journal.pcbi.1005059 27513946 PMC4981334

[25] McAvoy A, Hauert C. Asymmetric evolutionary games. PLoS Comput Biol. 2015;11(8):e1004349. doi: 10.1371/journal.pcbi.1004349 26308326 PMC4550251

[26] Barabási AL, Albert R. Emergence of scaling in random networks. Science. 1999;286(5439):509–512. doi: 10.1126/science.286.5439.509 10521342

[27] Albert R, Barabási AL. Topology of evolving networks: local events and universality. Phys Rev Lett. 2000;85(24):5234. doi: 10.1103/PhysRevLett.85.5234 11102229

[28] Dorogovtsev SN, Mendes JFF, Samukhin AN. Structure of growing networks with preferential linking. Phys Rev Lett. 2000;85(21):4633. doi: 10.1103/PhysRevLett.85.4633 11082614

[29] Goh KI, Kahng B, Kim D. Universal behavior of load distribution in scale-free networks. Phys Rev Lett. 2001;87(27):278701. doi: 10.1103/PhysRevLett.87.278701 11800921

[30] García-Pérez G, Boguñá M, Allard A, Serrano M. The hidden hyperbolic geometry of international trade: World Trade Atlas 1870–2013. Sci Rep. 2016;6(1):33441. doi: 10.1038/srep33441 27633649 PMC5025783

[31] Zheng M, García-Pérez G, Boguñá M, Serrano M. Scaling up real networks by geometric branching growth. Proc Natl Acad Sci U S A. 2021;118(21):e2018994118. doi: 10.1073/pnas.2018994118 34006638 PMC8166096

[32] Rao C, Coyte KZ, Bainter W, Geha RS, Martin CR, Rakoff-Nahoum S. Multi-kingdom ecological drivers of microbiota assembly in preterm infants. Nature. 2021;591(7851):633–638. doi: 10.1038/s41586-021-03241-8 33627867 PMC7990694

[33] Coyte KZ, Rao C, Rakoff-Nahoum S, Foster KR. Ecological rules for the assembly of microbiome communities. PLoS Biol. 2021;19(2):e3001116. doi: 10.1371/journal.pbio.3001116 33606675 PMC7946185

[34] Poncela J, Gómez-Gardenes J, Floría LM, Sánchez A, Moreno Y. Complex cooperative networks from evolutionary preferential attachment. PLoS ONE. 2008;3(6):e2449. doi: 10.1371/journal.pone.0002449 18560601 PMC2413409

[35] Poncela J, Gómez-Gardeñes J, Traulsen A, Moreno Y. Evolutionary game dynamics in a growing structured population. New J Phys. 2009;11(8):083031. doi: 10.1088/1367-2630/11/8/083031

[36] Perc M, Szolnoki A. Coevolutionary games—a mini review. BioSystems. 2010;99(2):109–125. doi: 10.1016/j.biosystems.2009.10.003 19837129

[37] Akçay E. Collapse and rescue of cooperation in evolving dynamic networks. Nat Commun. 2018;9(1):2692. doi: 10.1038/s41467-018-05130-7 30002374 PMC6043585

[38] Fu F, Hauert C, Nowak MA, Wang L. Reputation-based partner choice promotes cooperation in social networks. Phys Rev E. 2008;78(2):026117. doi: 10.1103/PhysRevE.78.026117 18850907 PMC2699261

[39] Wu B, Zhou D, Fu F, Luo Q, Wang L, Traulsen A. Evolution of cooperation on stochastic dynamical networks. PLoS ONE. 2010;5(6):e11187. doi: 10.1371/journal.pone.0011187 20614025 PMC2894855

[40] Holme P, Saramäki J. Temporal networks. Phys Rep. 2012;519(3):97–125. doi: 10.1016/j.physrep.2012.03.001

[41] Wu B, Altrock PM, Wang L, Traulsen A. Universality of weak selection. Phys Rev E. 2010;82(4):046106. doi: 10.1103/PhysRevE.82.046106 21230344

[42] Fotouhi B, Momeni N, Allen B, Nowak MA. Evolution of cooperation on large networks with community structure. J R Soc Interface. 2019;16(152):20180677. doi: 10.1098/rsif.2018.0677 30862280 PMC6451403

[43] Forber P, Smead R. The evolution of fairness through spite. Proc R Soc B. 2014;281(1780):20132439. doi: 10.1098/rspb.2013.2439 24523265 PMC4027385

[44] Steger A, Wormald NC. Generating random regular graphs quickly. Comb Probab Comput. 1999;8(4):377–396. doi: 10.1017/S0963548399003867

[45] Génois M, Barrat A. Can co-location be used as a proxy for face-to-face contacts? EPJ Data Sci. 2018;7(1):1–18.

[46] Isella L, Stehlé J, Barrat A, Cattuto C, Pinton JF, Van den Broeck W. What’s in a crowd? Analysis of face-to-face behavioral networks. J Theor Bio. 2011;271(1):166–180. doi: 10.1016/j.jtbi.2010.11.033 21130777

[47] Génois M, Vestergaard CL, Fournet J, Panisson A, Bonmarin I, Barrat A. Data on face-to-face contacts in an office building suggest a low-cost vaccination strategy based on community linkers. Network Sci. 2015;3(3):326–347. doi: 10.1017/nws.2015.10

[48] Vazquez A, Racz B, Lukacs A, Barabasi AL. Impact of non-Poissonian activity patterns on spreading processes. Phys Rev Lett. 2007;98(15):158702. doi: 10.1103/PhysRevLett.98.158702 17501392

[49] Iribarren JL, Moro E. Impact of human activity patterns on the dynamics of information diffusion. Phys Rev Lett. 2009;103(3):038702. doi: 10.1103/PhysRevLett.103.038702 19659326

[50] Redner S. How popular is your paper? An empirical study of the citation distribution. Eur Phys J B. 1998;4(2):131–134. doi: 10.1007/s100510050359

[51] Pastor-Satorras R, Vespignani A. Epidemic spreading in scale-free networks. Phys Rev Lett. 2001;86(14):3200. doi: 10.1103/PhysRevLett.86.3200 11290142

[52] Li A, Zhou L, Su Q, Cornelius SP, Liu YY, Wang L, et al. Evolution of cooperation on temporal networks. Nat Commun. 2020;11(1):2259. doi: 10.1038/s41467-020-16088-w 32385279 PMC7210286

[53] Cardillo A, Petri G, Nicosia V, Sinatra R, Gómez-Gardenes J, Latora V. Evolutionary dynamics of time-resolved social interactions. Phys Rev E. 2014;90(5):052825. doi: 10.1103/PhysRevE.90.052825 25493851

[54] Alvarez-Rodriguez U, Battiston F, de Arruda GF, Moreno Y, Perc M, Latora V. Evolutionary dynamics of higher-order interactions in social networks. Nat Hum Behav. 2021;5(5):586–595. doi: 10.1038/s41562-020-01024-1 33398148

[55] Battiston F, Amico E, Barrat A, Bianconi G, Ferraz de Arruda G, Franceschiello B, et al. The physics of higher-order interactions in complex systems. Nat Phys. 2021;17(10):1093–1098. doi: 10.1038/s41567-021-01371-4

[56] Perc M, Gómez-Gardenes J, Szolnoki A, Floría LM, Moreno Y. Evolutionary dynamics of group interactions on structured populations: a review. J R Soc Interface. 2013;10(80):20120997. doi: 10.1098/rsif.2012.0997 23303223 PMC3565747

