# Supplementary material for: Evolutionary dynamics on sequential temporal networks
Source: PLoS Comput Biol. 2023 Aug 7;19(8):e1011333. doi: 10.1371/journal.pcbi.1011333 (PMC10434888; doi:10.1371/journal.pcbi.1011333)
Supplement: S1 Text — Detailed calculations of fixation probabilities and critical benefit-to-cost ratios under different updating rules. Derivations of the mean-field approximation under different updating rules. Algorithms for constructing sequential temporal networks. (PDF) [file pcbi.1011333.s001.pdf]

Supporting Information

Evolutionary dynamics on sequential temporal networks

Anzhi Sheng, Aming Li and Long Wang

1 Evolutionary dynamics

In the main text, we consider two kinds of evolutionary processes on sequential temporal networks. We begin with the first one that the evolution on each snapshot is sufficient. In this case, the fixation of cooperation on a sequential temporal network can be seen as the fixation on each snapshot. Therefore, we separately analyze each snapshot to predict the dynamics on the sequential temporal network.

We consider a population of size N. The set of individuals is denoted as $\mathcal{V} = \{1, ..., N\}$. The state of the population is denoted as $\mathbf{x} = (x_1, ..., x_N)^{\mathrm{T}} \in \{0, 1\}^N$, where $x_i = 1$ or $x_i = 0$ indicates that the strategy of individual i is cooperation (C) or defection (D). Since the evolutionary process is mutation-free, there are two absorbing states, that is, the population is occupied by cooperators (denoted as $\mathbf{C} = (1, ..., 1)^{\mathrm{T}}$) or defectors (denoted as $\mathbf{D} = (0, ..., 0)^{\mathrm{T}}$).

We follow the workflow proposed by McAvoy & Allen [1] for analyzing the mutation-free evolutionary dynamics under weak selection ($\delta \ll 1$). Following the notation in the main text, each variable under neutral drift and weak selection is labeled with a superscript $^\circ$ and *, respectively. At each time step, a set of individuals $R \subset \mathcal{V}$ is chosen to be replaced. The replacement is defined by an offspring-to-parent map, $\alpha : R \to \mathcal{V}$, and $p_{(R,\alpha)}(\mathbf{x})$ denotes the probability of event (R, α) in state $\mathbf{x}$. Another useful mapping is the extended version of α, denoted as $\tilde{\alpha} : \mathcal{V} \to \mathcal{V}$, defined by $\tilde{\alpha}(j) = \alpha(j)$ if $j \in R$ and $\tilde{\alpha}(j) = j$ if $j \notin R$. We denote by $e_{ij}(\mathbf{x})$ the probability of i transmitting its strategy to j in state $\mathbf{x}$, given by

$$e_{ij}(\mathbf{x}) := \sum_{\substack{(R,\alpha) \\ \alpha(j)=i}} p_{(R,\alpha)}(\mathbf{x}),$$

and its derivative with respect to δ at $\delta = 0$ is given by

$$\left.\frac{\mathrm{d}}{\mathrm{d}\delta}\right|_{\delta=0} e_{ij}(\mathbf{x}) := \sum_{I \subset \mathcal{V}} c_I^{ij} \prod_{i \in I} x_i.$$

D_{ij} is defined as the degree of the higher-order nonzero term of c_I^{ij}, and D is defined as the maximum of D_{ij} for any i, j.

Let π_i denote the probability of fixation for cooperation when there is initially only a cooperator in site i, which is well-known as the reproductive value (RV) of i. These probabilities

are the unique solution to the following system of equations

$$\sum_{j=1}^{N} e^{\circ}_{ij}\pi_j = \sum_{j=1}^{N} e^{\circ}_{ji},$$
$$\sum_{j=1}^{N} \pi_i = 1.$$

Then for a given initial configuration $\boldsymbol{\xi} = (\xi_1, ..., \xi_N)^{\mathrm{T}} \in \{0,1\}^N$ (except **C** and **D**), the fixation probability for this initialization under neutral drift is given by $\widehat{\xi} = \sum_{i=1}^{N} \pi_i \xi_i$, and the corresponding fixation probability under weak selection is given by

$$\left(\rho^{\boldsymbol{\xi}}\right)^* = \widehat{\xi} + \delta \left(\sum_{i=1}^{N} \pi_i \sum_{j=1}^{N} \sum_{\substack{I \subset \mathcal{V} \\ 0 \le |I| \le D_{ji}}} c_I^{ji} \left(\eta^{\boldsymbol{\xi}}_{\{i\} \cup I} - \eta^{\boldsymbol{\xi}}_{\{j\} \cup I} \right) \right) + O\left(\delta^2\right) \tag{S1}$$

where $\eta^{\boldsymbol{\xi}}$ is the unique solution to the equations

$$\eta^{\boldsymbol{\xi}}_I = \widehat{\xi} - \boldsymbol{\xi}_I + \sum_{(R,\alpha)} p^{\circ}_{(R,\alpha)} \eta^{\boldsymbol{\xi}}_{\widetilde{\alpha}(I)} \quad (1 \le |I| \le D+1); \tag{S2a}$$

$$\sum_{i=1}^{N} \pi_i \eta^{\boldsymbol{\xi}}_{\{i\}} = 0. \tag{S2b}$$

For uniform initialization μ, the fixation probability under weak selection is given by

$$\left(\rho^{\mu}\right)^* = \frac{1}{N} + \delta \left(\sum_{i=1}^{N} \pi_i \sum_{j=1}^{N} \sum_{\substack{I \subset \mathcal{V} \\ 0 \le |I| \le D_{ji}}} c_I^{ji} \left(\eta^{\mu}_{\{i\} \cup I} - \eta^{\mu}_{\{j\} \cup I} \right) \right) + O\left(\delta^2\right) \tag{S3}$$

where η^{μ} is the unique solution to the equations

$$\eta^{\mu}_I = \begin{cases} \dfrac{1}{N} + \displaystyle\sum_{(R,\alpha)} p^{\circ}_{(R,\alpha)} \eta^{\mu}_{\widetilde{\alpha}(I)} & 2 \le |I| \le D+1, \\ 0 & |I| = 1. \end{cases} \tag{S4}$$

Let $\mathcal{T} = (\mathcal{S}, \mathcal{A}) = \{\mathcal{G}^{(1)}, ..., \mathcal{G}^{(L)}\}$ denote a sequential temporal network, where $\mathcal{S}$ is the topology when population growth finishes (i.e., the static counterpart), $\mathcal{A} = \{\mathbf{a}^{(1)}, ..., \mathbf{a}^{(L)}\}$ is a set of vectors recording the activity of node in each snapshot, L is the length of the sequential temporal network, and $\mathcal{G}^{(i)}$ is the ith snapshot. We consider the evolution on sequential temporal networks under uniform initialization. In this case, the success for the fixation of cooperation in sequential temporal networks requires the fixation of cooperation in each snapshot. Therefore, the fixation probability of sequential temporal networks is given

by

$$\rho_{\mathcal{T}}^{\mu} = \rho_{\mathcal{G}^{(1)}}^{\mu} \prod_{l=2}^{L} \rho_{\mathcal{G}^{(l)}}^{\mathbf{a}^{(l-1)}}, \tag{S5}$$

2 Specific update rules

A static network $\mathcal{S}$ is represented by a matrix $(w_{ij})_{i,j=1}^{N}$, where w_{ij} is the weight of edge (i,j), satisfying $w_{ij} = w_{ji}$, and $w_{ij} > 0$ if an edge exists between individuals i and j, $w_{ij} = 0$ otherwise. The node strength of node i is $w_i = \sum_{j=1}^{N} w_{ij}$, and the probability of a one-step random walk from i to j is given by $p_{ij}^{(1)} = w_{ij}/w_i$. The probability of i taking n steps to j is denoted as $p_{ij}^{(n)}$, and $p_{ij}^{(n+m)} = \sum_{k=1}^{N} p_{ik}^{(n)} p_{kj}^{(m)}$. We note that $p_{ij}^{(0)} = 1$ if $i = j$, otherwise $p_{ij}^{(0)} = 0$. The edge-weighted average payoff of node i, depending on the state $\mathbf{x}$, is $u_i(\mathbf{x}) = -cx_i + b\sum_{j=1}^{N} p_{ij}^{(1)} x_j$. The fitness of node i is given by $F_i(\mathbf{x}) = 1 + \delta u_i(\mathbf{x})$.

We consider three update rules:

1. Death-Birth (DB) updating [2]: At each time step, an individual i is selected with a uniform probability to die. A random neighbor j is selected with probability proportional to $w_{ji}F_j$ to compete for the empty site.

2. Pairwise-comparison (PC) updating [3]: At each time step, an individual i is selected with uniform probability and compares payoffs with a neighbor j selected with probability proportional to $p_{ij}^{(1)}$. j reproduces an offspring to replace i with probability $\frac{F_j}{F_i+F_j}$.

3. Imitation (IM) updating [2]: At each time step, an individual i is selected with uniform probability to evaluate its strategy. A random individual j including i and its neighbors reproduces an offspring to replace i with probability proportional to $w_{ji}F_j$ (here we set $w_{ii} = 1$).

For these update rules, only one individual is replaced at each time step, so the replacement event (R, α) is simplified as $(\{i\}, i \to j)$, which means that individual j reproduces an offspring to replace individual i. We note that i is allowed to equal j.

2.1 DB updating

Under DB updating, the probability of replacement event $(\{j\}, j \to i)$ in the state $\mathbf{x}$ is given by

$$p_{(\{j\},j\to i)}(\mathbf{x}) = \frac{1}{N}\frac{w_{ji}F_i(\mathbf{x})}{\sum_{l=1}^{N} w_{jl}F_l(\mathbf{x})}.$$

Under neutral drift,

$$p^{\circ}_{(\{j\},j\to i)}(\mathbf{x}) = \frac{p^{(1)}_{ji}}{N}. \tag{S6}$$

The derivative of $p^{\circ}_{(\{j\},j\to i)}(\mathbf{x})$ with respect to δ at $\delta = 0$ is

$$\left.\frac{\mathrm{d}}{\mathrm{d}\delta}\right|_{\delta=0} p^{\circ}_{(\{j\},j\to i)}(\mathbf{x}) = \frac{p^{(1)}_{ji}}{N}\left(u_i(\mathbf{x}) - \sum_{l=1}^{N} p^{(1)}_{jl} u_l(\mathbf{x})\right) = \sum_{k=1}^{N} c^{ij}_k x_k,$$

where

$$c^{ij}_k = \frac{p^{(1)}_{ji}}{N}\left(-c\left(p^{(0)}_{ik} - p^{(1)}_{jk}\right) + b\left(p^{(1)}_{ik} - p^{(2)}_{jk}\right)\right). \tag{S7}$$

In this case, the reproductive value of i is $\pi_i = w_i / \sum_{k=1}^{N} w_k$. Furthermore, we have the equation

$$\pi_i p^{(1)}_{ij} = \pi_j p^{(1)}_{ji}. \tag{S8}$$

Substituting Equation S7 into Equation S1 yields

$$\left(\rho^{\boldsymbol{\xi}}_{\mathcal{S}}\right)^{*} = \widehat{\xi} + \delta\left(\sum_{i,j,k=1}^{N} \pi_i \frac{p^{(1)}_{ij}}{N}\left(-c\left(p^{(0)}_{jk} - p^{(1)}_{ik}\right) + b\left(p^{(1)}_{jk} - p^{(2)}_{ik}\right)\right)\left(\eta^{\boldsymbol{\xi}}_{ik} - \eta^{\boldsymbol{\xi}}_{jk}\right)\right) + O\left(\delta^2\right).$$

To simplify notations, let

$$\eta^{\boldsymbol{\xi}}_{(k)} = \sum_{i,j=1}^{N} \pi_i p^{(k)}_{ij} \eta^{\boldsymbol{\xi}}_{ij}.$$

Using Equation S8, we have

$$\begin{aligned}
&\sum_{i,j,k=1}^{N} \pi_i p^{(1)}_{ij} p^{(0)}_{jk} \eta^{\boldsymbol{\xi}}_{ik} = \sum_{i,k=1}^{N} \pi_i p^{(1)}_{ik} \eta^{\boldsymbol{\xi}}_{ik} = \eta^{\boldsymbol{\xi}}_{(1)}, &\quad &\sum_{i,j,k=1}^{N} \pi_i p^{(1)}_{ij} p^{(0)}_{ik} \eta^{\boldsymbol{\xi}}_{jk} = \sum_{j} \pi_j \eta^{\boldsymbol{\xi}}_{jj} = 0,\\
&\sum_{i,j,k=1}^{N} \pi_i p^{(1)}_{ij} p^{(1)}_{ik} \eta^{\boldsymbol{\xi}}_{ik} = \sum_{i,k=1}^{N} \pi_i p^{(1)}_{ik} \eta^{\boldsymbol{\xi}}_{ik} = \eta^{\boldsymbol{\xi}}_{(1)}, &\quad &\sum_{i,j,k=1}^{N} \pi_i p^{(1)}_{ij} p^{(1)}_{ik} \eta^{\boldsymbol{\xi}}_{jk} = \sum_{j,k=1}^{N} \pi_j p^{(2)}_{jk} \eta^{\boldsymbol{\xi}}_{jk} = \eta^{\boldsymbol{\xi}}_{(2)},\\
&\sum_{i,j,k=1}^{N} \pi_i p^{(1)}_{ij} p^{(1)}_{jk} \eta^{\boldsymbol{\xi}}_{ik} = \sum_{i,k=1}^{N} \pi_i p^{(2)}_{ik} \eta^{\boldsymbol{\xi}}_{ik} = \eta^{\boldsymbol{\xi}}_{(2)}, &\quad &\sum_{i,j,k=1}^{N} \pi_i p^{(1)}_{ij} p^{(1)}_{jk} \eta^{\boldsymbol{\xi}}_{jk} = \sum_{j} \pi_j p^{(1)}_{jk} \eta^{\boldsymbol{\xi}}_{jk} = \eta^{\boldsymbol{\xi}}_{(1)},\\
&\sum_{i,j,k=1}^{N} \pi_i p^{(1)}_{ij} p^{(2)}_{ik} \eta^{\boldsymbol{\xi}}_{ik} = \sum_{i,k=1}^{N} \pi_i p^{(2)}_{ik} \eta^{\boldsymbol{\xi}}_{ik} = \eta^{\boldsymbol{\xi}}_{(2)}, &\quad &\sum_{i,j,k=1}^{N} \pi_i p^{(1)}_{ij} p^{(2)}_{ik} \eta^{\boldsymbol{\xi}}_{jk} = \sum_{j,k=1}^{N} \pi_j p^{(3)}_{jk} \eta^{\boldsymbol{\xi}}_{jk} = \eta^{\boldsymbol{\xi}}_{(3)}.
\end{aligned}$$

Therefore,

$$\left(\rho^{\boldsymbol{\xi}}_{\mathcal{S}}\right)^{*} = \widehat{\xi} + \frac{\delta}{N}\left(-c\eta^{\boldsymbol{\xi}}_{(2)} + b\left(\eta^{\boldsymbol{\xi}}_{(3)} - \eta^{\boldsymbol{\xi}}_{(1)}\right)\right) + O\left(\delta^2\right). \tag{S9}$$

We now turn to Equation S2a. For $i \neq j$,

$$
\begin{aligned}
\eta_{ij}^{\boldsymbol{\xi}} &= \widehat{\xi} - \xi_i \xi_j + \sum_{k,l=1}^{N} \frac{1}{N} p_{kl}^{(1)} \eta_{\widetilde{\alpha}(i)\widetilde{\alpha}(j)}^{\boldsymbol{\xi}} \\
&= \widehat{\xi} - \xi_i \xi_j + \frac{1}{N} \left(\sum_{k \notin \{i,j\}} \sum_{l=1}^{N} p_{kl}^{(1)} \eta_{ij}^{\boldsymbol{\xi}} + \sum_{l=1}^{N} p_{il}^{(1)} \eta_{lj}^{\boldsymbol{\xi}} + \sum_{l=1}^{N} p_{jl}^{(1)} \eta_{il}^{\boldsymbol{\xi}} \right) \\
&= \widehat{\xi} - \xi_i \xi_j + \frac{1}{N} \left((N-2) \eta_{ij}^{\boldsymbol{\xi}} + \sum_{l=1}^{N} p_{il}^{(1)} \eta_{lj}^{\boldsymbol{\xi}} + \sum_{l=1}^{N} p_{jl}^{(1)} \eta_{il}^{\boldsymbol{\xi}} \right)
\end{aligned}
$$

which gives

$$
\eta_{ij}^{\boldsymbol{\xi}} = \frac{N}{2} \left(\widehat{\xi} - \xi_i \xi_j \right) + \frac{1}{2} \sum_{l=1}^{N} \left(p_{il}^{(1)} \eta_{lj}^{\boldsymbol{\xi}} + p_{jl}^{(1)} \eta_{il}^{\boldsymbol{\xi}} \right). \tag{S10}
$$

For $i = j$,

$$
\begin{aligned}
\eta_{ii}^{\boldsymbol{\xi}} &= \widehat{\xi} - \xi_i + \sum_{k,l=1}^{N} \frac{1}{N} p_{kl}^{(1)} \eta_{\widetilde{\alpha}(i)\widetilde{\alpha}(i)}^{\boldsymbol{\xi}} \\
&= \widehat{\xi} - \xi_i + \frac{1}{N} \left(\sum_{k \neq i} \sum_{l=1}^{N} p_{kl}^{(1)} \eta_{ii}^{\boldsymbol{\xi}} + \sum_{l=1}^{N} p_{il}^{(1)} \eta_{ll}^{\boldsymbol{\xi}} \right) \\
&= \widehat{\xi} - \xi_i + \frac{1}{N} \left((N-1) \eta_{ii}^{\boldsymbol{\xi}} + \sum_{l=1}^{N} p_{il}^{(1)} \eta_{ll}^{\boldsymbol{\xi}} \right)
\end{aligned}
$$

which gives

$$
\eta_{ii}^{\boldsymbol{\xi}} = N \left(\widehat{\xi} - \xi_i \right) + \sum_{l=1}^{N} p_{il}^{(1)} \eta_{ll}^{\boldsymbol{\xi}}. \tag{S11}
$$

For uniform initialization, let

$$
\eta_{(k)}^{\mu} = \sum_{i,j=1}^{N} \pi_i p_{ij}^{(k)} \eta_{ij}^{\mu}.
$$

we have

$$
\left(\rho_{\mathcal{S}}^{\mu} \right)^{*} = \frac{1}{N} + \frac{\delta}{N} \left(-c \eta_{(2)}^{\mu} + b \left(\eta_{(3)}^{\mu} - \eta_{(1)}^{\mu} \right) \right) + O\left(\delta^2 \right), \tag{S12}
$$

and Equation S4 become

$$
\begin{aligned}
\eta_{ij}^{\mu} &= \frac{1}{2} + \frac{1}{2} \sum_{l=1}^{N} \left(p_{il}^{(1)} \eta_{lj}^{\mu} + p_{jl}^{(1)} \eta_{il}^{\mu} \right) \quad (i \neq j); \\
\eta_{ii}^{\mu} &= 0.
\end{aligned} \tag{S13}
$$

2.2 PC updating

Under PC updating, the probability of replacement event $(\{j\}, j \to i)$ and the marginal probability e_{ij} in state $\mathbf{x}$ is

$$p_{(\{j\},j\to i)}(\mathbf{x}) = e_{ij}(\mathbf{x}) = \begin{cases} \dfrac{1}{N} p_{ji}^{(1)} \dfrac{F_i(\mathbf{x})}{F_i(\mathbf{x}) + F_j(\mathbf{x})} & (i \neq j), \\ \dfrac{1}{N} \displaystyle\sum_{k=1}^{N} p_{jk}^{(1)} \dfrac{F_j(\mathbf{x})}{F_k(\mathbf{x}) + F_j(\mathbf{x})} & (i = j). \end{cases}$$

Under neutral drift,

$$p^{\circ}_{(\{j\},j\to i)}(\mathbf{x}) = \begin{cases} \dfrac{p_{ji}^{(1)}}{2N} & (i \neq j), \\ \dfrac{1}{2N} & (i = j). \end{cases}$$

The derivative of $p^{\circ}_{(\{j\},j\to i)}(\mathbf{x})$ with respect to δ at $\delta = 0$ is

$$\left.\frac{\mathrm{d}}{\mathrm{d}\delta}\right|_{\delta=0} p^{\circ}_{(\{j\},j\to i)}(\mathbf{x}) = \begin{cases} \dfrac{1}{N} p_{ji}^{(1)} \dfrac{u_i(\mathbf{x}) - u_j(\mathbf{x})}{4} & (i \neq j), \\ \dfrac{1}{n} \displaystyle\sum_{k=1}^{N} p_{jk}^{(1)} \dfrac{u_j(\mathbf{x}) - u_k(\mathbf{x})}{4} & (i = j). \end{cases} \tag{S14}$$

The marginal effect of k on i replacing j is

$$c_k^{ij} = \begin{cases} \dfrac{p_{ji}^{(1)}}{4N} \left(-c(p_{ik}^{(0)} - p_{jk}^{(0)})\right) + b\left(p_{ik}^{(1)} - p_{jk}^{(1)}\right) & (i \neq j), \\ \dfrac{1}{4N} \left(-c(p_{jk}^{(0)} - p_{jk}^{(1)})\right) + b\left(p_{jk}^{(1)} - p_{jk}^{(2)}\right) & (i = j). \end{cases} \tag{S15}$$

The reproductive value is again $\pi_i = w_i / \sum_{k=1}^{N} w_k$.

Substituting Equation S17 into Equation S1 yields

$$\left(\rho_{\mathcal{S}}^{\boldsymbol{\xi}}\right)^* = \hat{\xi} + \delta \left(\sum_{i,j,k=1}^{N} \pi_i \frac{p_{ij}^{(1)}}{4N} \left(-c\left(p_{jk}^{(0)} - p_{ik}^{(0)}\right) + b\left(p_{jk}^{(1)} - p_{ik}^{(1)}\right)\right)\left(\eta_{ik}^{\boldsymbol{\xi}} - \eta_{jk}^{\boldsymbol{\xi}}\right) \right) + O\left(\delta^2\right).$$

As before, we have

$$\left(\rho_{\mathcal{S}}^{\boldsymbol{\xi}}\right)^* = \hat{\xi} + \frac{\delta}{N}\left(-c\eta_{(1)}^{\boldsymbol{\xi}} + b\left(\eta_{(2)}^{\boldsymbol{\xi}} - \eta_{(1)}^{\boldsymbol{\xi}}\right)\right) + O\left(\delta^2\right). \tag{S16}$$

We then turn to Equation S2a. We see that for $i \neq j$

$$\eta_{ij}^{\boldsymbol{\xi}} = \frac{N}{2}\left(\hat{\xi} - \xi_i \xi_j\right) + \frac{1}{2}\sum_{l=1}^{N}\left(p_{il}^{(1)} \eta_{lj}^{\boldsymbol{\xi}} + p_{jl}^{(1)} \eta_{il}^{\boldsymbol{\xi}}\right). \tag{S17}$$

And for $i = j$,

$$\eta_{ii}^{\boldsymbol{\xi}} = N\left(\widehat{\xi} - \xi_i\right) + \sum_{l=1}^{N} p_{il}^{(1)} \eta_{ll}^{\boldsymbol{\xi}}. \tag{S18}$$

These recurrences are the same as that under DB updating.

For uniform initialization,

$$\left(\rho_{\mathcal{S}}^{\mu}\right)^{*} = \frac{1}{N} + \frac{\delta}{N}\left(-c\eta_{(1)}^{\mu} + b\left(\eta_{(2)}^{\mu} - \eta_{(1)}^{\mu}\right)\right) + O\left(\delta^2\right). \tag{S19}$$

and the corresponding recurrences are

$$\begin{aligned} \eta_{ij}^{\mu} &= \frac{1}{2} + \frac{1}{2}\sum_{l=1}^{N}\left(p_{il}^{(1)}\eta_{lj}^{\mu} + p_{jl}^{(1)}\eta_{il}^{\mu}\right) \quad (i \neq j); \\ \eta_{ii}^{\mu} &= 0. \end{aligned} \tag{S20}$$

2.3 IM updating

Under IM updating, the probability of replacement event $(\{j\}, j \to i)$ in the state $\mathbf{x}$ is

$$p_{(\{j\},j\to i)}(\mathbf{x}) = \begin{cases} \dfrac{1}{N}\dfrac{w_{ji}F_i(\mathbf{x})}{F_j(\mathbf{x}) + \sum_{k=1}^{N} w_{jk}F_k(\mathbf{x})} & (i \neq j), \\ \dfrac{1}{N}\dfrac{F_j(\mathbf{x})}{F_j(\mathbf{x}) + \sum_{k=1}^{N} w_{jk}F_k(\mathbf{x})} & (i = j). \end{cases}$$

To simplify notations, we define a new matrix $(\widetilde{w}_{ij})_{i,j=1}^{N}$, where $\widetilde{w}_{ij} = w_{ij}$ for all $i \neq j$ and $\widetilde{w}_{ii} = 1$ for all $i \in \mathcal{V}$. Let $\widetilde{p}_{ij} := \widetilde{w}_{ij}/\widetilde{w}_i$, under neutral drift,

$$p_{(\{j\},j\to i)}^{\circ}(\mathbf{x}) = \frac{\widetilde{p}_{ji}^{(1)}}{N}.$$

The derivative of $p_{(\{j\},j\to i)}^{\circ}(\mathbf{x})$ with respect to δ at $\delta = 0$ is

$$\left.\frac{\mathrm{d}}{\mathrm{d}\delta}\right|_{\delta=0} p_{(\{j\},j\to i)}^{\circ}(\mathbf{x}) = \frac{\widetilde{p}_{ji}^{(1)}}{N}\left(u_i(\mathbf{x}) - \sum_{l=1}^{N}\widetilde{p}_{jl}^{(1)}u_l(\mathbf{x})\right). \tag{S21}$$

The marginal effect of k on i replacing j is

$$c_k^{ij} = \frac{\widetilde{p}_{ji}^{(1)}}{N}\left(-c\left(p_{ik}^{(0)} - \widetilde{p}_{jk}^{(1)}\right) + b\left(p_{ik}^{(1)} - \sum_{m=1}^{N}\widetilde{p}_{jm}^{(1)}p_{mk}^{(1)}\right)\right). \tag{S22}$$

The reproductive value is $\pi_i = \widetilde{w}_i / \sum_{k=1}^{N} \widetilde{w}_k$. We also have the equation

$$\pi_i \widetilde{p}_{ij}^{(1)} = \pi_j \widetilde{p}_{ji}^{(1)}. \tag{S23}$$

Substituting Equation S22 into Equation S1 yields

$$\left(\rho_{\mathcal{S}}^{\boldsymbol{\xi}}\right)^{*} = \widehat{\xi} + \delta \left(\sum_{i,j,k=1}^{N} \pi_i \frac{\widetilde{p}_{ij}^{(1)}}{N} \left(-c \left(p_{jk}^{(0)} - \widetilde{p}_{ik}^{(1)} \right) + b \left(p_{jk}^{(1)} - \sum_{m=1}^{N} \widetilde{p}_{jm}^{(1)} p_{mk}^{(1)} \right) \right) \left(\eta_{ik}^{\boldsymbol{\xi}} - \eta_{jk}^{\boldsymbol{\xi}} \right) \right) + O\left(\delta^2\right).$$

Let

$$\widetilde{\eta}_{(m,n)}^{\boldsymbol{\xi}} = \sum_{i,j,k=1}^{N} \pi_i \widetilde{p}_{ik}^{(m)} p_{kj}^{(n)} \eta_{ij}^{\boldsymbol{\xi}}, \quad \widetilde{\eta}_{(m,n)}^{\mu} = \sum_{i,j,k=1}^{N} \pi_i \widetilde{p}_{ik}^{(m)} p_{kj}^{(n)} \eta_{ij}^{\mu}.$$

We obtain

$$\left(\rho_{\mathcal{S}}^{\boldsymbol{\xi}}\right)^{*} = \widehat{\xi} + \frac{\delta}{N} \left(-c \widetilde{\eta}_{(2,0)}^{\boldsymbol{\xi}} + b \left(\widetilde{\eta}_{(2,1)}^{\boldsymbol{\xi}} - \widetilde{\eta}_{(0,1)}^{\boldsymbol{\xi}} \right) \right) + O\left(\delta^2\right). \tag{S24}$$

Similarly, for $i \neq j$

$$\eta_{ij}^{\boldsymbol{\xi}} = \frac{N}{2} \left(\widehat{\xi} - \xi_i \xi_j \right) + \frac{1}{2} \sum_{l=1}^{N} \left(\widetilde{p}_{il}^{(1)} \eta_{lj}^{\boldsymbol{\xi}} + \widetilde{p}_{jl}^{(1)} \eta_{il}^{\boldsymbol{\xi}} \right). \tag{S25}$$

And for $i = j$,

$$\eta_{ii}^{\boldsymbol{\xi}} = N \left(\widehat{\xi} - \xi_i \right) + \sum_{l=1}^{N} \widetilde{p}_{il}^{(1)} \eta_{ll}^{\boldsymbol{\xi}}. \tag{S26}$$

For uniform initialization,

$$\left(\rho_{\mathcal{S}}^{\mu}\right)^{*} = \widehat{\xi} + \frac{\delta}{N} \left(-c \widetilde{\eta}_{(2,0)}^{\mu} + b \left(\widetilde{\eta}_{(2,1)}^{\mu} - \widetilde{\eta}_{(0,1)}^{\mu} \right) \right) + O\left(\delta^2\right). \tag{S27}$$

and the corresponding recurrences are

$$\begin{aligned} \eta_{ij}^{\mu} &= 1 + \frac{1}{2} \sum_{l=1}^{N} \left(\widetilde{p}_{il}^{(1)} \eta_{lj}^{\mu} + \widetilde{p}_{jl}^{(1)} \eta_{il}^{\mu} \right) \quad (i \neq j); \\ \eta_{ii}^{\mu} &= 0. \end{aligned} \tag{S28}$$

3 Condition for promotion on sequential temporal networks

In this section, we compare the fixation probability of sequential temporal networks and corresponding static networks under these three updating rules.

3.1 Neutral drift

We notice that under any update rule,

$$\left(\rho_{\mathcal{S}}^{\xi}\right)^{\circ}=\widehat{\xi}=\frac{\sum_{i=1}^{N} w_i\xi_i}{\sum_{i=1}^{N} w_i},\quad \left(\rho_{\mathcal{S}}^{\mu}\right)^{\circ}=\frac{1}{N}.$$

For a sequential temporal network $\mathcal{T}=(\mathcal{S},\mathcal{A})=\{\mathcal{G}^{(1)},...,\mathcal{G}^{(L)}\}$, the fixation probability under neutral drift is given by

$$\left(\rho_{\mathcal{T}}^{\mu}\right)^{\circ}=\frac{1}{\sum_{i=1}^{N} a_i^{(1)}}\prod_{l=2}^{L}\sum_{i=1}^{N}\pi_i|_{\mathcal{G}^{(l)}}a_i^{(l-1)},$$

where $\pi_i|_{\mathcal{G}^{(l)}}$ represents π_i in $\mathcal{G}^{(l)}$.

3.1.1 DB and PC updating

The reproductive value of i is the same under DB and PC updating, given by $\widehat{\xi}=\sum_{i=1}^{N} w_i\xi_i/\sum_{i=1}^{N} w_i$. So a node with a higher node strength has a greater reproductive value and therefore greater influence on the evolutionary dynamics.

We first focus on sequential temporal networks with $L=2$ (i.e., $\mathcal{T}=\{\mathcal{G}^{(1)},\mathcal{G}^{(2)}\}$ and $\mathcal{S}=\mathcal{G}^{(2)}$). We assume that $\mathcal{G}^{(1)}$ ($\mathcal{G}^{(2)}$) has m ($m+\Delta m$) nodes and the average degree of $\mathcal{G}^{(1)}$ ($\mathcal{G}^{(2)}$) is k_1 (k_2). Furthermore, we assume that there are no edges among the newly added nodes Δm in $\mathcal{G}^{(2)}$.

The equivalent condition of $\left(\rho_{\mathcal{T}}^{\mu}\right)^{\circ}>\left(\rho_{\mathcal{S}}^{\mu}\right)^{\circ}$ is

$$\frac{1}{m}\frac{mk_1+(m+\Delta m)k_2}{2(m+\Delta m)k_2}>\frac{1}{m+\Delta m}. \tag{S29}$$

Equation S29 is equivalent to

$$m(m+\Delta m)k_1-k_2(m+\Delta m)(m-\Delta m)>0. \tag{S30}$$

It is straightforward to obtain the first condition in the main text (Equation 6a),

$$\Delta m\geq m. \tag{S31}$$

For the second condition, we notice that

$$mk_1+2\Delta K=k_2(m+\Delta m),$$

where ΔK is the number of newly added edges. Combined with Equation S30, when $\Delta m<m$,

we obtain the second condition

$$\Delta K < \frac{1}{2}\left(\frac{m(m+\Delta m)k_1}{m-\Delta m} - mk_1\right) = \frac{m\Delta m k_1}{m-\Delta m}. \tag{S32}$$

When $m \ll \Delta m$, the condition is transformed into

$$\Delta K < \Delta m k_1.$$

Moreover, we study three variations of the model: the rule of increasing the fixation probability for static networks, the rule in weighted networks, and the rule when internal edges exist in newly added nodes.

Variation 1: From another perspective, for a given static network $\mathcal{S}$, we can make the fixation probability larger by constructing a sequential temporal network. We assume that $\mathcal{G}^{(1)}$ ($\mathcal{G}^{(2)}$) has $m - \Delta m$ (m) nodes and the average degree of $\mathcal{G}^{(1)}$ ($\mathcal{G}^{(2)}$) is k_1 (k_2). There are no edges between the deleted nodes Δm in $\mathcal{S}$. Similarly, we get the equivalent condition for promotion

$$\frac{1}{m-\Delta m}\frac{(m-\Delta m)k_1 + mk_2}{2mk_2} > \frac{1}{m}. \tag{S33}$$

We denote by ΔK the number of edges removed from $\mathcal{S}$, then Equation S33 is equivalent to

$$\Delta K < \Delta m k_2,$$

meaning that the number of deleted edges should be less than the number of edges ($\Delta m k_2$) formed by the number of deleted nodes under the average degree of $\mathcal{S}$.

Variation 2: We assume that the average weight of $\mathcal{G}^{(1)}$ ($\mathcal{G}^{(2)}$) is w_1 (w_2). The conditions for cooperation are

$$(i)\ \Delta m \geq m,$$
$$(ii)\ \Delta m < m,\ \Delta K < \frac{m\Delta m w_1}{m-\Delta m}.$$

Variation 3: We assume that there are g_1 internal edges nodes and g_2 external edges connecting to $\mathcal{G}^{(1)}$ in Δm newly added nodes. The upper bound of g_1 is $\Delta m(\Delta m - 1)/2$.

Similar to Equations S31 and S32, the condition for promotion is

$$\frac{1}{m}\frac{mk_1 + g_2}{mk_1 + 2(g_1+g_2)} > \frac{1}{m+\Delta m} \Leftrightarrow m\Delta m k_1 + (\Delta m - m)g_2 - 2mg_1 > 0. \tag{S34}$$

When $g_1 > \Delta m k_1/2$, meaning that new nodes are highly connected with each other, the condition becomes

$$\Delta m > m, \quad g_2 > \frac{2mg_1 - m\Delta m k_1}{\Delta m - m}.$$

When $g_1 < \Delta m k_1/2$, the condition becomes

$$\Delta m \geq m,$$

or

$$\Delta m < m, \quad g_2 < \frac{m\Delta m k_1 - 2mg_1}{m - \Delta m}.$$

Especially, when $g_1 = 0$, the condition degenerates to Equations S33 and S34.

When newly joined nodes have no internal edges with each other in each snapshot, the necessary and sufficient condition of $\left(\rho_{\mathcal{T}}^{\mu}\right)^{\circ} > \left(\rho_{\mathcal{S}}^{\mu}\right)^{\circ}$ for any $L \geq 3$ is

$$\frac{1}{m_1}\prod_{l=2}^{L}\frac{m_{l-1}k_{l-1} + m_l k_l}{2m_l k_l} > \frac{1}{m_L k_L},$$

where m_l and k_l are the number of nodes and average connectivity of $\mathcal{G}^{(l)}$, respectively. In particular, using mathematical induction, a sufficient condition of $\left(\rho_{\mathcal{T}}^{\mu}\right)^{\circ} > \left(\rho_{\mathcal{S}}^{\mu}\right)^{\circ}$ for any $L \geq 3$ is every pair of adjacent snapshots satisfies Equation S33 or Equation S34.

3.1.2 IM updating

Here, we turn to the IM updating. The fixation probability $\left(\rho_{\mathcal{S}}^{\xi}\right)^{\circ}$ is given by

$$\hat{\xi} = \frac{\sum_{i=1}^{N}\widetilde{w}_i\xi_i}{\sum_{i=1}^{N}\widetilde{w}_i} = \frac{\sum_{i=1}^{N}(w_i+1)\xi_i}{\sum_{i=1}^{N}w_i + N}.$$

We begin with $L = 2$. The notations of sequential temporal networks are the same as that in section 3.1.1. The equivalent condition of $\left(\rho_{\mathcal{T}}^{\mu}\right)^{\circ} > \left(\rho_{\mathcal{S}}^{\mu}\right)^{\circ}$ is

$$\frac{1}{m}\frac{m(k_1+1) + ((m+\Delta m)k_2 - mk_1)/2}{(m+\Delta m)(k_2+1)} > \frac{1}{m+\Delta m},$$

which gives

$$(m+\Delta m)\Delta K + m\Delta m k_1 > 2m\Delta K.$$

This condition is the same as that in section 3.1.1.

For $L \geq 3$, the necessary and sufficient condition of $\left(\rho_{\mathcal{T}}^{\mu}\right)^{\circ} > \left(\rho_{\mathcal{S}}^{\mu}\right)^{\circ}$ is

$$\frac{1}{m_1}\prod_{l=2}^{L}\frac{m_{l-1}k_{l-1} + m_l k_l + 2m_{l-1}}{2m_l(k_l+1)} > \frac{1}{m_L k_L}.$$

3.2 Weak selection

Using mathematical induction, we can demonstrate that when the relation $\left(\rho_{\mathcal{T}}^{\mu}\right)^{\circ} > \left(\rho_{\mathcal{S}}^{\mu}\right)^{\circ}$ holds, the relation $\left(\rho_{\mathcal{T}}^{\mu}\right)^{*} > \left(\rho_{\mathcal{S}}^{\mu}\right)^{*}$ also holds. Therefore, we consider the equivalent condition for promotion under weak selection only when $\left(\rho_{\mathcal{T}}^{\mu}\right)^{\circ} = \left(\rho_{\mathcal{S}}^{\mu}\right)^{\circ}$. In this case, we compare the first-order term of $\left(\rho_{\mathcal{T}}^{\mu}\right)^{*}$ and $\left(\rho_{\mathcal{S}}^{\mu}\right)^{*}$, and ignore the higher-order terms.

We start with $L = 2$. The first-order coefficient of two types of fixation probabilities on snapshot $\mathcal{G}^{(i)}$ is given by

$$g(\mathcal{G}^{(i)};\boldsymbol{\xi}) = \begin{cases} \left[\frac{1}{N}\left(-c\eta_{(2)}^{\boldsymbol{\xi}} + b(\eta_{(3)}^{\boldsymbol{\xi}} - \eta_{(1)}^{\boldsymbol{\xi}})\right)\right]\Big|_{\mathcal{G}^{(i)}} & \text{(DB updating)}, \\ \left[\frac{1}{N}\left(-c\eta_{(1)}^{\boldsymbol{\xi}} + b(\eta_{(2)}^{\boldsymbol{\xi}} - \eta_{(1)}^{\boldsymbol{\xi}})\right)\right]\Big|_{\mathcal{G}^{(i)}} & \text{(PC updating)}, \\ \left[\frac{1}{N}\left(-c\widetilde{\eta}_{(2,0)}^{\boldsymbol{\xi}} + b(\widetilde{\eta}_{(2,1)}^{\boldsymbol{\xi}} - \widetilde{\eta}_{(0,1)}^{\boldsymbol{\xi}})\right)\right]\Big|_{\mathcal{G}^{(i)}} & \text{(IM updating)}. \end{cases} \tag{S35}$$

$$g(\mathcal{G}^{(i)};\mu) = \begin{cases} \left[\frac{1}{N}\left(-c\eta_{(2)}^{\mu} + b(\eta_{(3)}^{\mu} - \eta_{(1)}^{\mu})\right)\right]\Big|_{\mathcal{S}^{(i)}} & \text{(DB updating)}, \\ \left[\frac{1}{N}\left(-c\eta_{(1)}^{\mu} + b(\eta_{(2)}^{\mu} - \eta_{(1)}^{\mu})\right)\right]\Big|_{\mathcal{S}^{(i)}} & \text{(PC updating)}, \\ \left[\frac{1}{N}\left(-c\widetilde{\eta}_{(2)}^{\mu} + b(\widetilde{\eta}_{(2,1)}^{\mu} - \widetilde{\eta}_{(0,1)}^{\mu})\right)\right]\Big|_{\mathcal{S}^{(i)}} & \text{(IM updating)}. \end{cases} \tag{S36}$$

and the condition of $\left(\rho_{\mathcal{T}}^{\mu}\right)^{*} > \left(\rho_{\mathcal{S}}^{\mu}\right)^{*}$ is

$$\frac{1}{N}\Big|_{\mathcal{G}^{(1)}} g(\mathcal{G}^{(2)};\mathbf{a}^{(1)}) + \widehat{\xi}\Big|_{\mathcal{G}^{(2)}} g(\mathcal{G}^{(1)};\mu) > g(\mathcal{G}^{(2)};\mu), \tag{S37}$$

where $\frac{1}{N}\Big|_{\mathcal{G}^{(i)}}$ means the size of snapshot $\mathcal{G}^{(i)}$, and $\widehat{\xi}\Big|_{\mathcal{G}^{(i)}}$ means the RV-weighted value of $\widehat{\xi}$ on snapshot $\mathcal{G}^{(i)}$.

For $L \geq 3$, let $A_1 = (1/N)\Big|_{\mathcal{G}^{(1)}}$ and $A_i = \mathbf{a}^{(i-1)}\Big|_{\mathcal{G}^{(i)}}$ $(i = 2, ..., L)$, the necessary and sufficient condition of $\left(\rho_{\mathcal{T}}^{\mu}\right)^{*} > \left(\rho_{\mathcal{S}}^{\mu}\right)^{*}$ is given by

$$\widetilde{A}_1 g(\mathcal{G}^{(1)};\mu) + \sum_{i=2}^{L} \widetilde{A}_i g(\mathcal{G}^{(i)};\mathbf{a}^{(i-1)}) > g(\mathcal{G}^{(L)};\mu), \tag{S38}$$

where $\widetilde{A}_i = \prod_{j\neq i} A_j$. Once again, when each pair of adjacent snapshots of $\mathcal{T}$ satisfies Equation S37, we also have $\left(\rho_{\mathcal{T}}^{\mu}\right)^{*} > \left(\rho_{\mathcal{S}}^{\mu}\right)^{*}$.

4 Critical benefit-to-cost ratio

We also use the critical benefit-to-cost ratio $(b/c)^*$ to compare the cooperation-promoting effect of sequential temporal networks and static networks. We say a sequential temporal network $\mathcal{T}$ promotes cooperation if

$$\left(\frac{b}{c}\right)^*_{\mathcal{S};\mu} > \left(\frac{b}{c}\right)^*_{\mathcal{T}} > 0, \tag{S39a}$$

$$\left(\frac{b}{c}\right)^*_{\mathcal{S};\mu} < 0 < \left(\frac{b}{c}\right)^*_{\mathcal{T}}. \tag{S39b}$$

For an arbitrary initial configuration $\boldsymbol{\xi}$, the critical benefit-to-cost ratio of $\mathcal{S}$ is given as

$$\left(\frac{b}{c}\right)^*_{\mathcal{S};\boldsymbol{\xi}} = \begin{cases} \dfrac{\eta^{\boldsymbol{\xi}}_{(2)}}{\eta^{\boldsymbol{\xi}}_{(3)} - \eta^{\boldsymbol{\xi}}_{(1)}} & \text{(DB updating)}, \\ \dfrac{\eta^{\boldsymbol{\xi}}_{(1)}}{\eta^{\boldsymbol{\xi}}_{(2)} - \eta^{\boldsymbol{\xi}}_{(1)}} & \text{(PC updating)}, \\ \dfrac{\widetilde{\eta}^{\boldsymbol{\xi}}_{(2,0)}}{\widetilde{\eta}^{\boldsymbol{\xi}}_{(2,1)} - \widetilde{\eta}^{\boldsymbol{\xi}}_{(0,1)}} & \text{(IM updating)}. \end{cases} \tag{S40}$$

For uniform initialization, the critical benefit-to-cost ratio is

$$\left(\frac{b}{c}\right)^*_{\mathcal{S};\mu} = \begin{cases} \dfrac{\eta^{\mu}_{(2)}}{\eta^{\mu}_{(3)} - \eta^{\mu}_{(1)}} & \text{(DB updating)}, \\ ,\dfrac{\eta^{\mu}_{(1)}}{\eta^{\mu}_{(2)} - \eta^{\mu}_{(1)}} & \text{(PC updating)}, \\ \dfrac{\widetilde{\eta}^{\mu}_{(2,0)}}{\widetilde{\eta}^{\mu}_{(2,1)} - \widetilde{\eta}^{\mu}_{(0,1)}} & \text{(IM updating)}. \end{cases} \tag{S41}$$

For a sequential temporal network $\mathcal{T}$ with $L \geq 2$, the critical benefit-to-cost ratio is

$$\left(\frac{b}{c}\right)^*_{\mathcal{T}} = \begin{cases} \dfrac{\widetilde{A}_1\left(\frac{\eta^{\mu}_{I(2)}}{N}\right)\Big|_{\mathcal{G}^{(1)}} + \left(\sum_{i=2}^{L}\widetilde{A}_i\left(\frac{\eta^{\mathbf{a}^{(i-1)}}_{I(2)}}{N}\right)\Big|_{\mathcal{G}^{(i)}}\right)}{\widetilde{A}_1\left(\frac{\eta^{\mu}_{I(3)}-\eta^{\mu}_{I(1)}}{N}\right)\Big|_{\mathcal{G}^{(1)}} + \left(\sum_{i=2}^{L}\widetilde{A}_i\left(\frac{\eta^{\mathbf{a}^{(i-1)}}_{I(3)}-\eta^{\mathbf{a}^{(i-1)}}_{I(1)}}{N}\right)\Big|_{\mathcal{G}^{(i)}}\right)} & \text{(DB updating)} \\[2em] \dfrac{\widetilde{A}_1\left(\frac{\eta^{\mu}_{I(1)}}{N}\right)\Big|_{\mathcal{G}^{(1)}} + \left(\sum_{i=2}^{L}\widetilde{A}_i\left(\frac{\eta^{\mathbf{a}^{(i-1)}}_{I(1)}}{N}\right)\Big|_{\mathcal{G}^{(i)}}\right)}{\widetilde{A}_1\left(\frac{\eta^{\mu}_{I(2)}-\eta^{\mu}_{I(1)}}{N}\right)\Big|_{\mathcal{G}^{(1)}} + \left(\sum_{i=2}^{L}\widetilde{A}_i\left(\frac{\eta^{\mathbf{a}^{(i-1)}}_{I(2)}-\eta^{\mathbf{a}^{(i-1)}}_{I(1)}}{N}\right)\Big|_{\mathcal{G}^{(i)}}\right)} & \text{(PC updating)} \\[2em] \dfrac{\widetilde{A}_1\left(\frac{\eta^{\mu}_{I(2,0)}}{N}\right)\Big|_{\mathcal{G}^{(1)}} + \left(\sum_{i=2}^{L}\widetilde{A}_i\left(\frac{\eta^{\mathbf{a}^{(i-1)}}_{I(2,0)}}{N}\right)\Big|_{\mathcal{G}^{(i)}}\right)}{\widetilde{A}_1\left(\frac{\eta^{\mu}_{I(2,1)}-\eta^{\mu}_{I(0,1)}}{N}\right)\Big|_{\mathcal{G}^{(1)}} + \left(\sum_{i=2}^{L}\widetilde{A}_i\left(\frac{\eta^{\mathbf{a}^{(i-1)}}_{I(2,1)}-\eta^{\mathbf{a}^{(i-1)}}_{I(0,1)}}{N}\right)\Big|_{\mathcal{G}^{(i)}}\right)} & \text{(IM updating)} \end{cases} \quad \text{(S42)}$$

where $\widetilde{A}_i$ is the same as that in Equation S38. Substituting Equations S40 and S42 into Equation S39 yields a general condition about the critical ratio.

5 Mean-field approximation

In this section, we develop the mean-field approximation proposed in Ref. [4] to calculate the fixation probability and the critical benefit-to-cost ratio under weak selection.

We begin with DB and PC updating. For any arbitrary initial configuration $\boldsymbol{\xi} = (\xi_1, ..., \xi_N)$ except $\mathbf{C}, \mathbf{D}$, let $t_{ij} = \frac{N}{2}(\widehat{\xi} - \xi_i \xi_j)$ $(i \neq j)$ and $t_{ii} = 1/2$ for all i. We have a recurrence relation

for $\eta^{\xi}_{(n)}$,

$$\begin{aligned}
\eta^{\xi}_{(n)} &= \sum_{i,j=1}^{N} \pi_i p^{(n)}_{ij} \eta^{\xi}_{ij} \\
&= \sum_{\substack{i,j \in \mathcal{S} \\ i \neq j}} \pi_i p^{(n)}_{ij} \left(t_{ij} + \frac{1}{2} \sum_{k=1}^{N} \left(p_{ik} \eta^{\xi}_{kj} + p_{jk} \eta^{\xi}_{ik} \right) \right) + \sum_{i=1}^{N} \pi_i p^{(n)}_{ii} \eta^{\xi}_{ii} \\
&= \sum_{i,j=1}^{N} \pi_i p^{(n)}_{ij} \left(t_{ij} + \frac{1}{2} \sum_{k=1}^{N} \left(p_{ik} \eta^{\xi}_{kj} + p_{jk} \eta^{\xi}_{ik} \right) \right) + \sum_{i=1}^{N} \pi_i p^{(n)}_{ii} \eta^{\xi}_{ii} \\
&- \sum_{i=1}^{N} \pi_i p^{(n)}_{ii} \left(t_{ii} + \frac{1}{2} \sum_{k=1}^{N} \left(p_{ik} \eta^{\xi}_{ki} + p_{ik} \eta^{\xi}_{ik} \right) \right) \\
&= \sum_{i,j=1}^{N} \pi_i p^{(n)}_{ij} t_{ij} + \frac{1}{2} \sum_{j,k=1}^{N} \pi_j p^{(n+1)}_{jk} \eta^{\xi}_{jk} + \frac{1}{2} \sum_{i,k=1}^{N} \pi_j p^{(n+1)}_{ik} \eta^{\xi}_{ik} + \sum_{i=1}^{N} \pi_i p^{(n)}_{ii} \eta_{ii} \\
&- \sum_{i=1}^{N} \pi_i p^{(n)}_{ii} t_{ii} - \sum_{i,k=1}^{N} \pi_i p^{(n)}_{ii} p_{ik} \eta^{\xi}_{ik} \\
&= \sum_{i,j=1}^{N} \pi_i p^{(n)}_{ij} t_{ij} + \eta^{\xi}_{(n+1)} + \sum_{i=1}^{N} \pi_i p^{(n)}_{ii} \eta^{\xi}_{ii} - \sum_{i=1}^{N} \pi_i p^{(n)}_{ii} t_{ii} - \sum_{i,k=1}^{N} \pi_i p^{(n)}_{ii} p_{ik} \eta^{\xi}_{ik}.
\end{aligned}$$

To further simplify notations, let $q_i = \sum_{k=1}^{N} (p_{ik} \eta^{\xi}_{ik} - \eta^{\xi}_{ii}) + t_{ii}$, the recurrence relation is simplified as

$$\eta^{\xi}_{(n+1)} = \eta^{\xi}_{(n)} + \sum_{i=1}^{N} \pi_i p^{(n)}_{ii} q_i - \sum_{i,j=1}^{N} \pi_i p^{(n)}_{ij} t_{ij}. \tag{S43}$$

In particular, we have

$$\begin{aligned}
\eta^{\xi}_{(0)} &= \sum_{i,j=1}^{N} \pi_i p^{(0)}_{ij} \eta_{ij} = \sum_{i=1}^{N} \pi_i \eta_{ii} = 0, \\
\eta^{\xi}_{(1)} &= \sum_{i=1}^{N} \pi_i q_i - \sum_{i,j=1}^{N} \pi_i p^{(0)}_{ij} t_{ij}, \\
\eta^{\xi}_{(2)} &= \sum_{i=1}^{N} \pi_i q_i (1 + p^{(1)}_{ii}) - \sum_{i,j=1}^{N} \pi_i t_{ij} (p^{(0)}_{ij} + p^{(1)}_{ij}), \\
\eta^{\xi}_{(3)} &= \sum_{i=1}^{N} \pi_i q_i (1 + p^{(1)}_{ii} + p^{(2)}_{ii}) - \sum_{i,j=1}^{N} \pi_i t_{ij} (p^{(0)}_{ij} + p^{(1)}_{ij} + p^{(2)}_{ij}).
\end{aligned} \tag{S44}$$

Similarly, let $\widetilde{q}_i = \sum_{k=1}^{N} (\widetilde{p}_{ik} \eta^{\xi}_{ik} - \eta^{\xi}_{ii}) + t_{ii}$, the recurrence relation under IM updating is

$$\eta^{\xi}_{(n+1,0)} = \eta^{\xi}_{(n,0)} + \sum_{i=1}^{N} \pi_i \widetilde{p}^{(n)}_{ii} \widetilde{q}_i - \sum_{i,j=1}^{N} \pi_i \widetilde{p}^{(n)}_{ij} t_{ij}, \tag{S45}$$

and

$$\begin{aligned}
\eta^{\xi}_{(0,0)} &= 0, \\
\eta^{\xi}_{(1,0)} &= \sum_{i=1}^{N} \pi_i \widetilde{q}_i - \sum_{i,j=1}^{N} \pi_i \widetilde{p}^{(0)}_{ij} t_{ij}, \\
\eta^{\xi}_{(2,0)} &= \sum_{i=1}^{N} \pi_i \widetilde{q}_i (1 + \widetilde{p}^{(1)}_{ii}) - \sum_{i,j=1}^{N} \pi_i t_{ij} (\widetilde{p}^{(0)}_{ij} + \widetilde{p}^{(1)}_{ij}), \\
\eta^{\xi}_{(3,0)} &= \sum_{i} \pi_i \widetilde{q}_i (1 + \widetilde{p}^{(1)}_{ii} + \widetilde{p}^{(2)}_{ii}) - \sum_{i,j=1}^{N} \pi_i t_{ij} (\widetilde{p}^{(0)}_{ij} + \widetilde{p}^{(1)}_{ij} + \widetilde{p}^{(2)}_{ij}).
\end{aligned} \tag{S46}$$

Since $\mathcal{S}$ is connected, random walks on $\mathcal{S}$ are ergodic [5]. Therefore, the equation $\lim_{n\to\infty} p^{(n)}_{ij} = \pi_i$ ($\lim_{n\to\infty} \widetilde{p}^{(n)}_{ij} = \pi_i$) holds for all $j \in \mathcal{S}$ and $\lim_{n\to\infty} \eta^{\xi}_{(n)} = \eta^{\xi}_{\infty}$ ($\lim_{n\to\infty} \eta^{\xi}_{(n,0)} = \eta^{\xi}_{(\infty,0)}$) hold. Letting $n \to \infty$ in Equations S43 and S45, we obtain the relation

$$\begin{aligned}
\sum_{i=1}^{N} \pi_i^2 q_i &= \sum_{i,j\in\mathcal{S}} \pi_i \pi_j t_{ij}, \\
\sum_{i=1}^{N} \pi_i^2 \widetilde{q}_i &= \sum_{i,j\in\mathcal{S}} \pi_i \pi_j t_{ij}.
\end{aligned} \tag{S47}$$

The right-hand side of Equation S47 is denoted by B_0, which is independent to η^{ξ}_{ij}.

Let $\mu_1 = \sum_{i=1}^{N} w_i/N$ ($\widetilde{\mu}_1 = \sum_{i=1}^{N} \widetilde{w}_i/N$) and $\mu_2 = \sum_{i=1}^{N} w_i^2/N$ ($\widetilde{\mu}_2 = \sum_{i=1}^{N} \widetilde{w}_i^2/N$) denote the first and second moments of the network weighted degree distribution. Equation S47 is transformed to

$$\begin{aligned}
\sum_{i=1}^{N} w_i^2 q_i &= B_0 (N\mu_1)^2, \\
\sum_{i=1}^{N} \widetilde{w}_i^2 \widetilde{q}_i &= B_0 (N\widetilde{\mu}_1)^2.
\end{aligned} \tag{S48}$$

Applying the mean-field approximation, we obtain

$$\begin{aligned}
q_i &= \frac{B_0 N \mu_1^2}{\mu_2}, \\
\widetilde{q}_i &= \frac{B_0 N \widetilde{\mu}_1^2}{\widetilde{\mu}_2}.
\end{aligned} \tag{S49}$$

In the case of uniform initialization, we obtain

$$\xi_i \xi_j = 0, \ \forall i \neq j,$$

and

$$\widehat{\xi} = \frac{1}{N},$$

which gives $t_{ij} = \frac{1}{2}$ for all $i, j \in \mathcal{S}$ and $B_0 = \frac{1}{2}$. Substituting these into Equations S43 and S45, we obtain the recurrence relation for uniform initialization, which is the same as Eq. (27) in Ref. [6].

As $p_{ii}^{(1)} = 0$ for all i, we approximate $\eta_{(n)}^{\boldsymbol{\xi}}$ and $\eta_{(n)}^{\mu}$ as follows,

$$\begin{aligned}
\eta_{(1)}^{\boldsymbol{\xi}} &\approx \frac{B_0 N \mu_1^2}{\mu_2} - \frac{1}{N\mu_1}\sum_{i=1}^{N} w_i t_{ii}, \\
\eta_{(2)}^{\boldsymbol{\xi}} &\approx \frac{B_0 N \mu_1^2}{\mu_2} - \frac{1}{N\mu_1}\left(\sum_{i,j=1}^{N} w_i (p_{ij}^{(0)} + p_{ij}^{(1)}) t_{ij}\right), \\
\eta_{(3)}^{\boldsymbol{\xi}} &\approx \frac{B_0 N \mu_1^2}{\mu_2} + \frac{B_0 \mu_1 \sum_{i=1}^{N} w_i p_{ii}^{(2)}}{\mu_2} - \frac{1}{N\mu_1}\left(\sum_{i,j=1}^{N} w_i (p_{ij}^{(0)} + p_{ij}^{(1)} + p_{ij}^{(2)}) t_{ij}\right), \\
\eta_{(1)}^{\mu} &\approx \frac{N\mu_1^2}{2\mu_2} - \frac{1}{2}, \\
\eta_{(2)}^{\mu} &\approx \frac{N\mu_1^2}{2\mu_2} - 1, \\
\eta_{(3)}^{\mu} &\approx \frac{N\mu_1^2}{2\mu_2} + \frac{\sum_{i=1}^{N} w_i p_{ii}^{(2)} \mu_1}{2\mu_2} - \frac{3}{2}.
\end{aligned} \tag{S50}$$

When $w_i \gg 1$ for all i, we have $p_{ij} \approx \widetilde{p}_{ij}$ for all $i \neq j$. Considering $\widetilde{p}_{ii}^{(1)} \neq 0$ for all i, we see that

$$\begin{aligned}
\eta_{(1,0)}^{\boldsymbol{\xi}} &\approx \eta_{(0,1)}^{\boldsymbol{\xi}} \approx \frac{B_0 N \widetilde{\mu}_1^2}{\widetilde{\mu}_2} - \frac{1}{N\widetilde{\mu}_1}\sum_{i=1}^{N} \widetilde{w}_i t_{ii}, \\
\eta_{(2,0)}^{\boldsymbol{\xi}} &\approx \sum_{i=1}^{N} \pi_i (1 + \widetilde{p}_{ii}^{(1)}) \frac{B_0 N \widetilde{\mu}_1^2}{\widetilde{\mu}_2} - \frac{1}{N\widetilde{\mu}_1}\left(\sum_{i,j=1}^{N} \widetilde{w}_i (\widetilde{p}_{ij}^{(0)} + \widetilde{p}_{ij}^{(1)}) t_{ij}\right), \\
\eta_{(3,0)}^{\boldsymbol{\xi}} &\approx \eta_{(2,1)}^{\boldsymbol{\xi}} \approx \sum_{i=1}^{N} \pi_i (1 + \widetilde{p}_{ii}^{(1)} + \widetilde{p}_{ii}^{(2)}) \frac{B_0 N \widetilde{\mu}_1^2}{\widetilde{\mu}_2} - \frac{1}{N\widetilde{\mu}_1}\left(\sum_{i,j=1}^{N} \widetilde{w}_i (\widetilde{p}_{ij}^{(0)} + \widetilde{p}_{ij}^{(1)} + \widetilde{p}_{ij}^{(2)}) t_{ij}\right), \\
\eta_{(1,0)}^{\mu} &\approx \eta_{(0,1)}^{\mu} \approx \frac{N\widetilde{\mu}_1^2}{2\widetilde{\mu}_2} - \frac{1}{2}, \\
\eta_{(2,0)}^{\mu} &\approx \sum_{i=1}^{N} \pi_i (1 + \widetilde{p}_{ii}^{(1)}) \frac{N\widetilde{\mu}_1^2}{2\widetilde{\mu}_2} - 1, \\
\eta_{(3,0)}^{\mu} &\approx \eta_{(2,1)}^{\mu} \approx \sum_{i=1}^{N} \pi_i (1 + \widetilde{p}_{ii}^{(1)} + \widetilde{p}_{ii}^{(2)}) \frac{N\widetilde{\mu}_1^2}{2\widetilde{\mu}_2} - \frac{3}{2}.
\end{aligned} \tag{S51}$$

Therefore, the fixation probabilities $\left(\rho_{\mathcal{S}}^{\boldsymbol{\xi}}\right)^*$ and $\left(\rho_{\mathcal{S}}^{\mu}\right)^*$ can be approximated as

$$\left(\rho_{\mathcal{S}}^{\boldsymbol{\xi}}\right)^* \approx \begin{cases} \widehat{\xi} + \dfrac{\delta}{N}\begin{pmatrix} -c\left(\frac{B_0 N\mu_1^2}{\mu_2} - \frac{1}{N\mu_1}(C_0+C_1)\right) \\ +b\left(\frac{B_0\Lambda\mu_1}{\mu_2} - \frac{1}{N\mu_1}(C_1+C_2)\right)\end{pmatrix} & \text{(DB updating)}, \\ \widehat{\xi} + \dfrac{\delta}{N}\begin{pmatrix} -c\left(\frac{B_0 N\mu_1^2}{\mu_2} - \frac{1}{N\mu_1}C_0\right) \\ +b\left(-\frac{1}{N\mu_1}C_1\right)\end{pmatrix} & \text{(PC updating)}, \\ \widehat{\xi} + \dfrac{\delta}{N}\begin{pmatrix} -c\left(\sum_{i=1}^N \pi_i(1+\widetilde{p}_{ii}^{(1)})\frac{B_0 N\widetilde{\mu}_1^2}{\widetilde{\mu}_2} - \frac{1}{N\widetilde{\mu}_1}(\widetilde{C}_0+\widetilde{C}_1)\right) \\ +b\left(\frac{B_0\widetilde{\Lambda}\widetilde{\mu}_1}{\widetilde{\mu}_2} - \frac{1}{N\widetilde{\mu}_1}(\widetilde{C}_1+\widetilde{C}_2)\right)\end{pmatrix} & \text{(IM updating)}, \end{cases} \quad \text{(S52)}$$

and

$$\left(\rho_{\mathcal{S}}^{\mu}\right)^* \approx \begin{cases} \dfrac{1}{N} + \dfrac{\delta}{N}\left(-c\left(\dfrac{N\mu_1^2}{2\mu_2} - 1\right) + b\left(\dfrac{\Lambda\mu_1}{2\mu_2} - 1\right)\right) & \text{(DB updating)}, \\ \dfrac{1}{N} + \dfrac{\delta}{N}\left(-c\left(\dfrac{N\mu_1^2}{2\mu_2} - \dfrac{1}{2}\right) - \dfrac{1}{2}b\right) & \text{(PC updating)}, \\ \dfrac{1}{N} + \dfrac{\delta}{N}\left(-c\left(\sum_{i=1}^N \pi_i(1+\widetilde{p}_{ii}^{(1)})\dfrac{N\widetilde{\mu}_1^2}{2\widetilde{\mu}_2} - 1\right) + b\left(\dfrac{\widetilde{\Lambda}\widetilde{\mu}_1}{2\widetilde{\mu}_2} - 1\right)\right) & \text{(IM updating)}, \end{cases} \quad \text{(S53)}$$

where $C_k = \sum_{i,j=1}^N w_i p_{ij}^{(k)} t_{ij}$ ($\widetilde{C}_k = \sum_{i,j=1}^N \widetilde{w}_i \widetilde{p}_{ij}^{(k)} t_{ij}$) for $k = 0,1,2$, and $\Lambda = \sum_{i=1}^N w_i p_{ii}^{(2)}$ ($\widetilde{\Lambda} = \sum_{i=1}^N \widetilde{w}_i(\widetilde{p}_{ii}^{(1)} + \widetilde{p}_{ii}^{(2)})$). The approximate critical benefit-to-cost ratio of $\mathcal{S}$ in the case of a specific configuration $\boldsymbol{\xi}$ and uniform initialization is given as

$$\left(\frac{b}{c}\right)^*_{\mathcal{S};\boldsymbol{\xi}} \approx \begin{cases} \dfrac{B_0 N^2\mu_1^3 - \mu_2(C_0+C_1)}{B_0 N\Lambda\mu_1^2 - \mu_2(C_1+C_2)} & \text{(DB updating)}, \\ \dfrac{\mu_2 C_0 - B_0 N^2\mu_1^3}{\mu_2 C_1} & \text{(PC updating)}, \\ \dfrac{\sum_{i=1}^N \pi_i(1+\widetilde{p}_{ii}^{(1)})B_0 N^2\widetilde{\mu}_1^3 - \widetilde{\mu}_2(\widetilde{C}_0+\widetilde{C}_1)}{B_0 N\widetilde{\Lambda}\widetilde{\mu}_1^2 - \widetilde{\mu}_2(\widetilde{C}_1+\widetilde{C}_2)} & \text{(IM updating)}, \end{cases} \quad \text{(S54)}$$

and

$$\left(\frac{b}{c}\right)^*_{\mathcal{S};\mu} \approx \begin{cases} \dfrac{N\mu_1^2 - 2\mu_2}{\Lambda\mu_1 - 2\mu_2} & \text{(DB updating)}, \\ \dfrac{\mu_2 - N\mu_1^2}{\mu_2} & \text{(PC updating)}, \\ \dfrac{\sum_{i=1}^N \pi_i(1+\widetilde{p}_{ii}^{(1)})N\widetilde{\mu}_1^2 - 2\widetilde{\mu}_2}{\widetilde{\Lambda}\widetilde{\mu}_1 - 2\widetilde{\mu}_2} & \text{(IM updating)}. \end{cases} \quad \text{(S55)}$$

All the notations in Equations S52-S55 are unrelated to $\eta_{ij}^{\boldsymbol{\xi}}$ and η_{ij}^{μ}, so that we can avoid solving the linear systems.

6 The monotonicity about g

Let $\rho_{\mathcal{T}}^{\mu}(g)$ denote the fixation probability for cooperation in the second evolutionary process that the evolution lasts for g generation in each snapshot (except the last one). Figures 4 and 5 in the main text shows that $\left(\rho_{\mathcal{T}}^{\mu}(g)\right)^*$ is monotonically increasing with respect to g. Here we give corresponding evidence under neutral drift. We first consider $\mathcal{T} = \{\mathcal{G}^{(1)}, \mathcal{G}^{(2)}\}$. We assume that $\mathcal{G}^{(1)}$ ($\mathcal{G}^{(2)}$) has m ($m + \Delta m$) nodes, the average connectivity is k_1 (k_2), and there are initially j cooperators in $\mathcal{G}^{(1)}$. After a one-step DB updating ($g = 1$), in the average sense, the probability of being $j-1$ cooperators in $\mathcal{G}^{(1)}$ is

$$\alpha_j = \frac{j}{m} \sum_{i=0}^{k_1} \frac{\binom{m-j}{i}\binom{j-1}{k_1-i}}{\binom{m-1}{k_1}} \frac{i}{k_1} = \frac{j(m-j)}{m(m-1)},$$

and the probability of being $j+1$ cooperators is

$$\beta_j = \frac{m-j}{m} \sum_{i=0}^{k_1} \frac{\binom{j}{i}\binom{m-j-1}{k_1-i}}{\binom{m-1}{k_1}} \frac{i}{k_1} = \frac{j(m-j)}{m(m-1)},$$

and the probability of remaining j cooperators is

$$\gamma_i = 1 - \alpha_i - \beta_i.$$

When entering $\mathcal{G}^{(2)}$ with the above distribution of cooperators, and assuming that each cooperator adds s edges on average, we obtain

$$\left(\rho_{\mathcal{T}}^{\mu}(1)\right)^\circ = \alpha_j \frac{(j-1)k_1 + (j-1)s}{(m+\Delta m)k_2} + \gamma_j \frac{jk_1 + js}{(m+\Delta m)k_2} + \beta_j \frac{(j+1)k_1 + (j+1)s}{(m+\Delta m)k_2} = \frac{jk_1}{(m+\Delta m)k_2}.$$

Then the condition for $\left(\rho_{\mathcal{T}}^{\mu}(1)\right)^\circ > \left(\rho_{\mathcal{T}}^{\mu}(0)\right)^\circ = \frac{j}{m+\Delta m}$ is

$$k_1 > k_2, \tag{S56}$$

or the equivalent expression

$$\Delta K < \frac{k_1 \Delta m}{2}, \tag{S57}$$

which is similar to Equation 6b in the main text. Note that Equations S56 and S57 are independent to the number of cooperators j.

Based on the above analysis, after a one-step update, a single case of j cooperators will be split into three cases ($j-1, j, j+1$) with a certain distribution. When Equation S57 holds, a single-step update can increase the fixation probability. When the number of rounds g increases, the number of the update also increases, so it is more likely to promote the evolution of cooperation when the parameter g is larger. The ability of the promote reaches the

maximum when the parameter $g = \infty$. This explains why the fixation probability increases monotonically with g.

7 Algorithms for constructing sequential temporal networks

7.1 Synthetic networks

We provide a detailed construction of the sequential temporal networks based on square lattices, random regular graphs, Barabási-Albert scale-free networks, and scale-free networks with initial attractiveness mentioned in the main text. Supplementary Figures 1 and 2 show the construction for a square lattice and a Barabási-Albert scale-free network, respectively. The network construction process of scale-free networks with the initial attractiveness is quite similar to Barabási-Albert scale-free networks.

For random regular graphs, we provide a general construction algorithm, which can also be used on square lattices, scale-free networks, and other network structures.

Algorithm 1 Construction of sequential temporal networks

Input: A static network topology $\mathcal{S}$.
Output: A sequential temporal network $\mathcal{T}$.

```
1: Randomly select a node and m_0 its neighbours to form an initial snapshot;
2: while #(active nodes) < N do
3:     Randomly select a node, which is a neighbour of an active node, to be activated;
```

This algorithm ensures that each snapshot is connected.

7.2 Empirical networks

For empirical data analysis, we use four datasets collected by the SocioPatterns collaboration (http://www.sociopatterns.org). The dataset of the infectious dynamic contact is divided according to the date, and we select the first three days (2009.04.28-2009.04.30) for analysis. For the other three datasets, we use the full data.

In the datasets, a contact event is formatted as a triplet (t, i, j), which represents an interaction between two individuals i and j occurring at time t. Based on the triplets, we construct the corresponding static and temporal networks. In the first step, we aggregate all contacts into a single undirected graph denoted by $\mathcal{S}_{agg}$. If the graph $\mathcal{S}_{agg}$ is not connected, we select the giant component of $\mathcal{S}_{agg}$ as a new static network. Subsequently, we determine the activity of individuals according to the contact events. For each individual, we find the first communication time t_1 and set $a_i^{(j)} = 1$ for all $j \geq t_1$. Then, we have a vector set $\mathcal{A} = \{\mathbf{a}^{(1)}, ..., \mathbf{a}^{(L)}\}$, and

the sequential temporal network $\mathcal{T} = \{\mathcal{G}^{(1)}, ..., \mathcal{G}^{(L)}\}$ is generated by $\mathcal{S}_{agg}$ and $\mathcal{A}$. Since each snapshot $\mathcal{G}^{(i)}$ may be disconnected, we select all connected snapshots to form the sequential temporal network $\mathcal{T} = \{\mathcal{G}^{(s_1)}, ..., \mathcal{G}_{agg}\}$.

References

[1] McAvoy, A. & Allen, B. Fixation probabilities in evolutionary dynamics under weak selection. *Journal of Mathematical Biology* **82**, 1–41 (2021).

[2] Ohtsuki, H., Lieberman, E., Hauert, C. & Nowak, M. A. A simple rule for the evolution of cooperation on graphs and social networks. *Nature* **441**, 502–505 (2006).

[3] McAovy, A., Allen, B. & Nowak, M. A. Social goods dilemmas in heterogeneous societies. *Nature Human Behaviour* **4**, 819–831 (2020).

[4] Fotouhi, B., Momeni, N., Allen, B. & Nowak, M. A. Evolution of cooperation on large networks with community structure. *Journal of the Royal Society Interface* **16**, 20180677 (2019).

[5] Ross, S. M., Kelly, J. J., Sullivan, R. J. et al. *Stochastic processes* (Wiley New York, 1996).

[6] Allen, B., Lippner, G., Chen, Y.-T., Fotouhi, B., Momeni, N., Yau, S.-T. & Nowak, M. A. Evolutionary dynamics on any population structure. *Nature* **544**, 227–230 (2017).
