# Supplementary figures and images for: Evolutionary dynamics on sequential temporal networks
Source: PLoS Comput Biol. 2023 Aug 7;19(8):e1011333. doi: 10.1371/journal.pcbi.1011333 (PMC10434888; doi:10.1371/journal.pcbi.1011333)

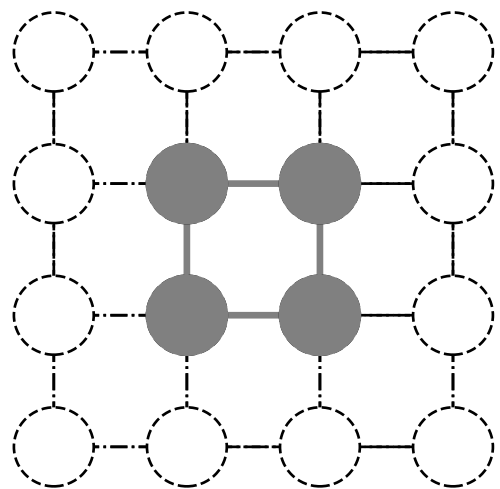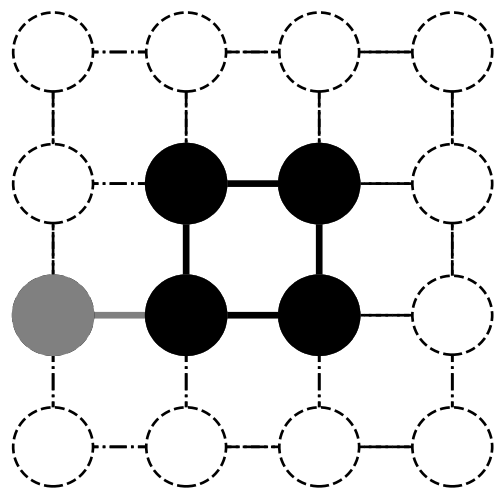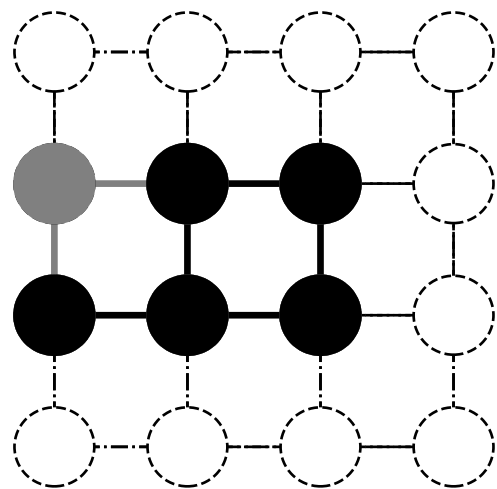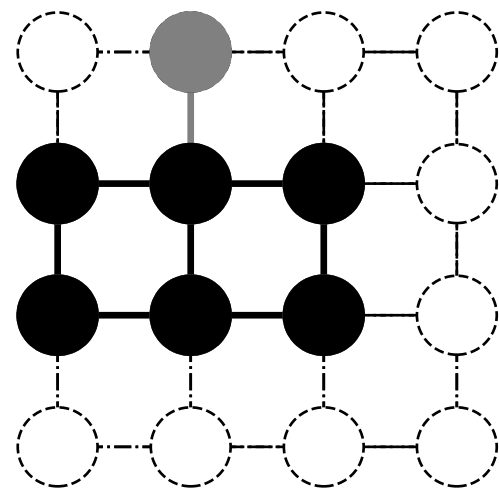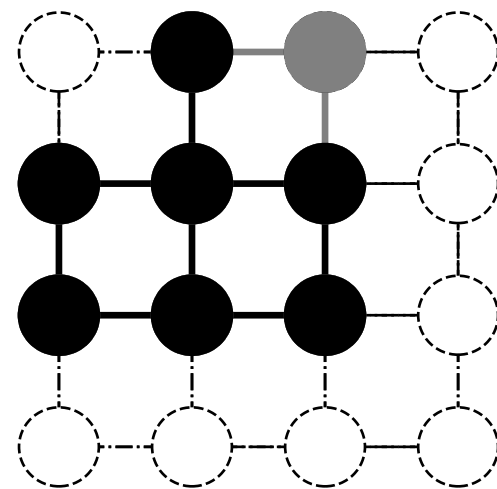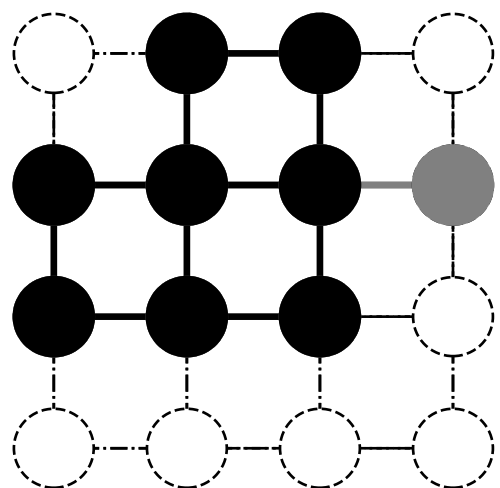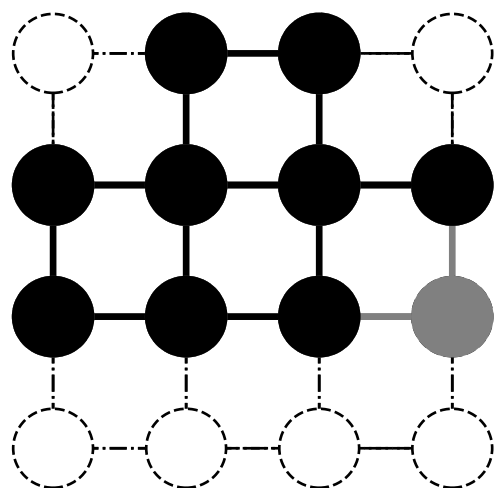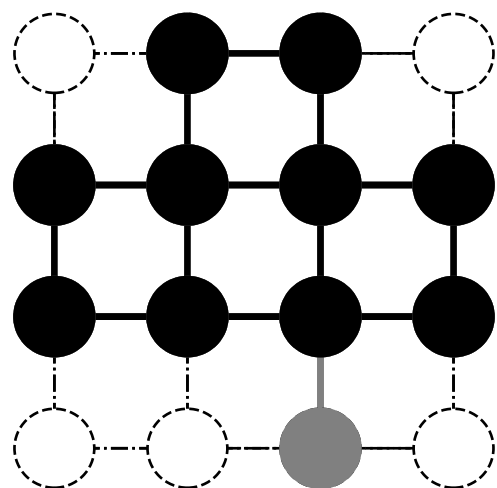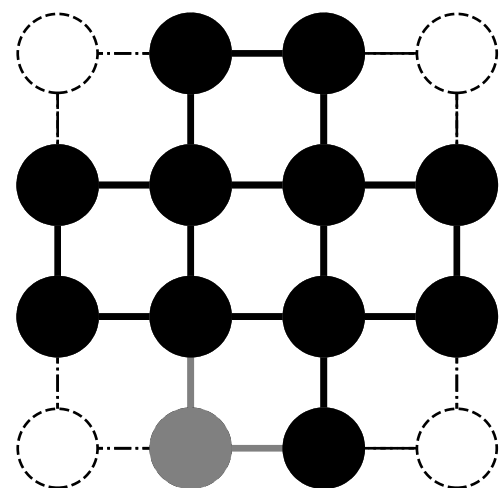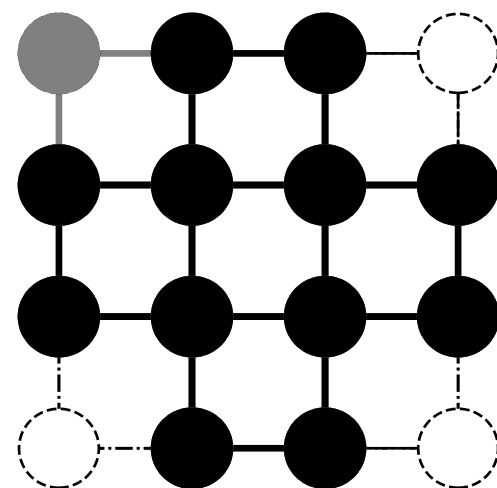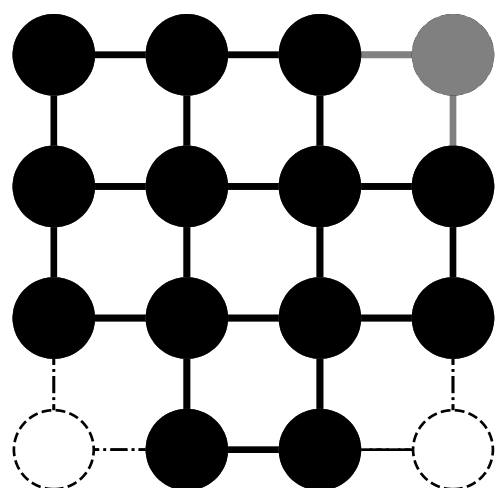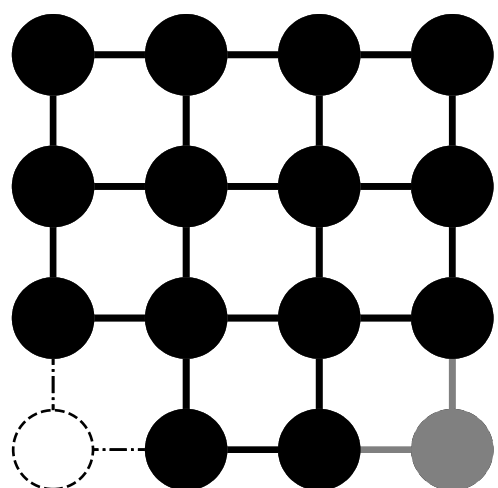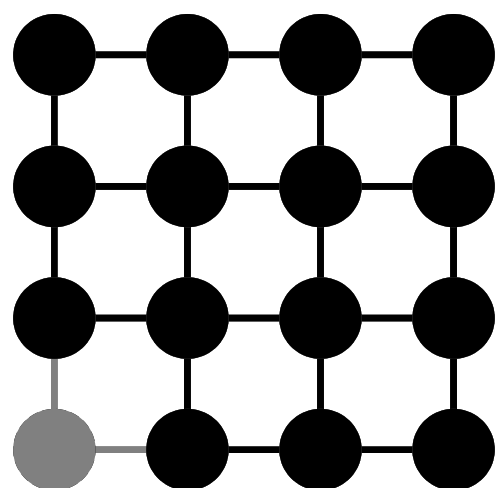

● / ○ / ● New active / Inactive / Previously active node  
 — / - - - / — New active / Inactive / Previously active edge

Supplement: S1 Fig — A schematic illustration of constructing a sequential temporal network on a square lattice of size N = 16. First, four nodes in the middle of a square lattice are set to be active. Then, at each time step, we activate one node in turn clockwise along the circle. The construction finishes when all nodes are active. (PDF) [file pcbi.1011333.s002.pdf]

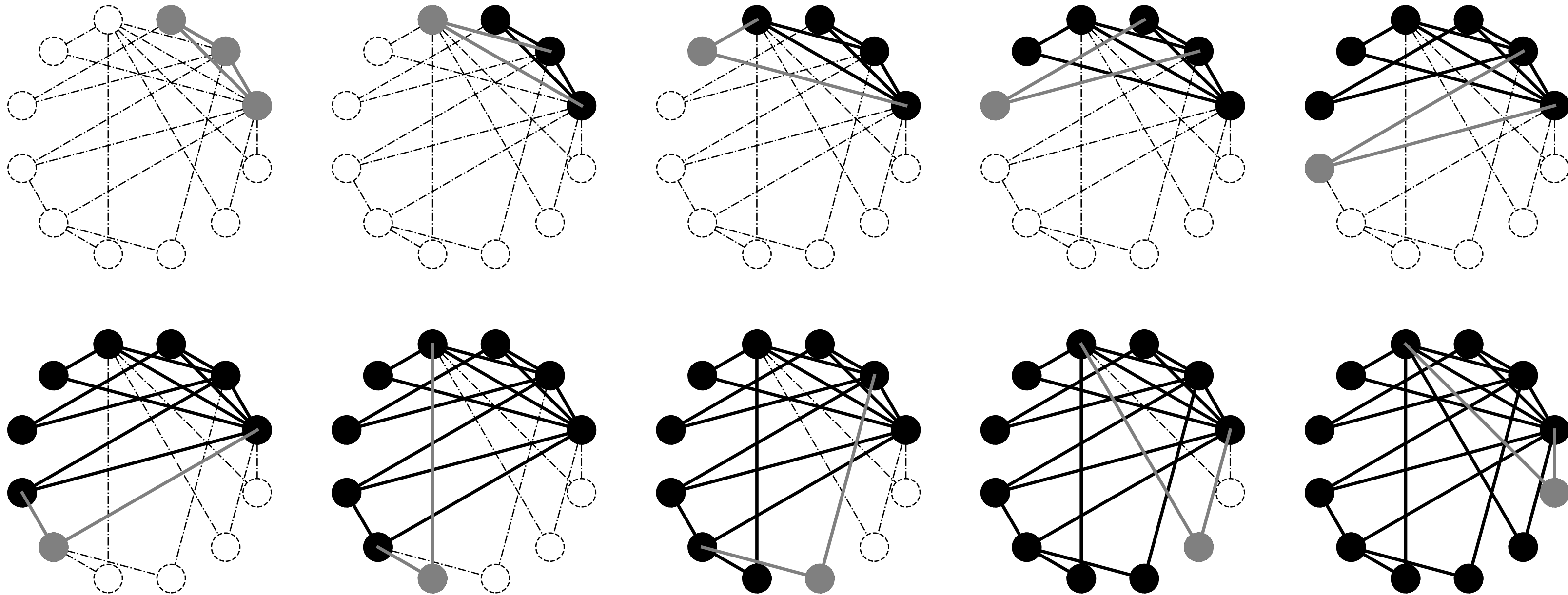

Supplement: S2 Fig — A schematic illustration of constructing a sequential temporal network on a Barabási-Albert scale-free network of size N = 12 and linking number m = 2. First, we activate m0 nodes to form an initial snapshot. Due to the growth and preferential attachment, at each time step, a new node enters the network and connects to m old nodes. At this point, the new node is active. The length of sequential temporal networks is equal to the number of new nodes plus one. (PDF) [file pcbi.1011333.s003.pdf]

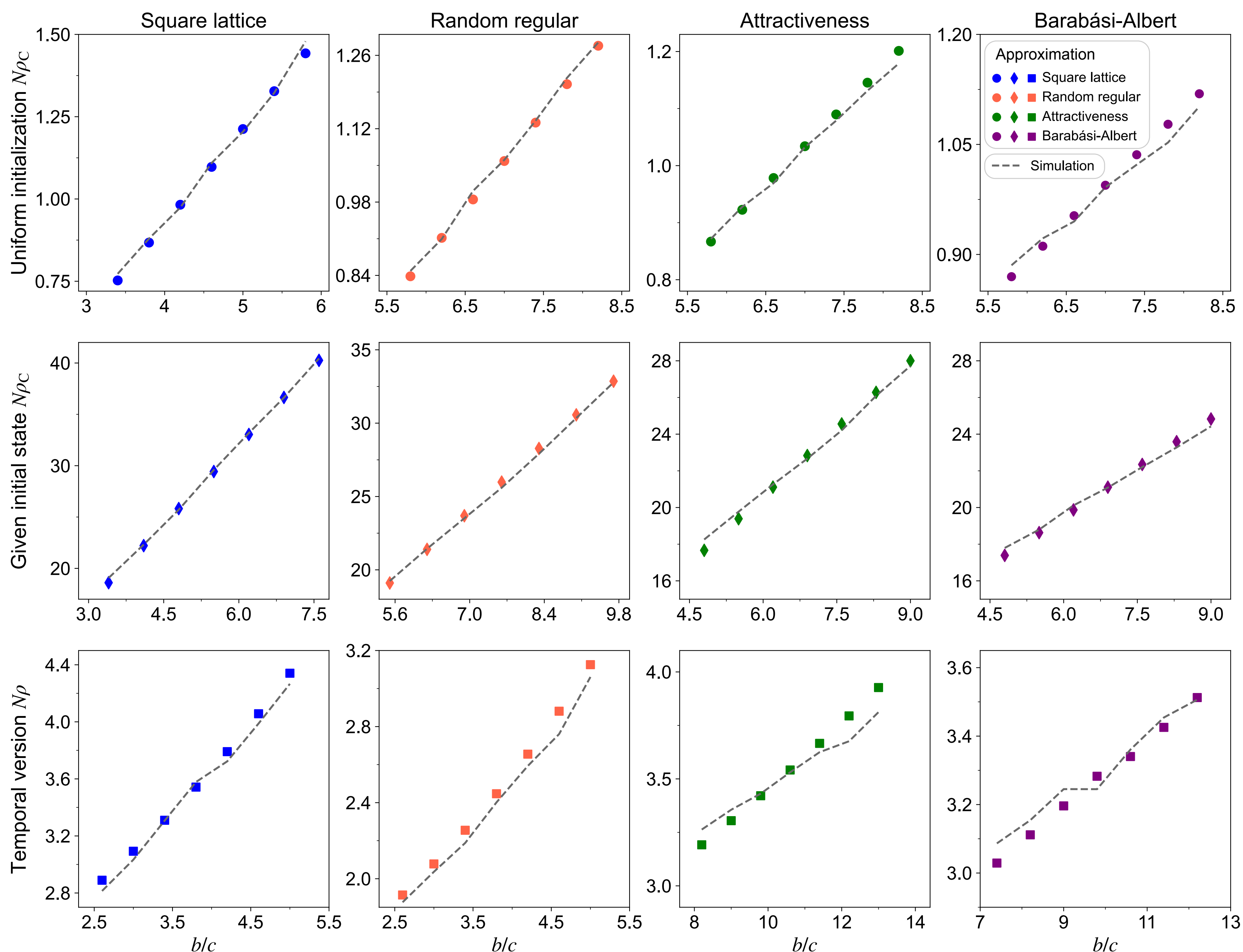

Supplement: S3 Fig — Comparisons between the fixation probabilities for cooperation obtained by numerical simulations (dashed lines) or the mean-field approximation (dots). The fixation probabilities are calculated under three setups: static networks with uniform initialization and a given initial configuration, and sequential temporal networks. Network structures and parameter values are the same as in Fig 4 in the main text. (PDF) [file pcbi.1011333.s004.pdf]

Uniform initialization

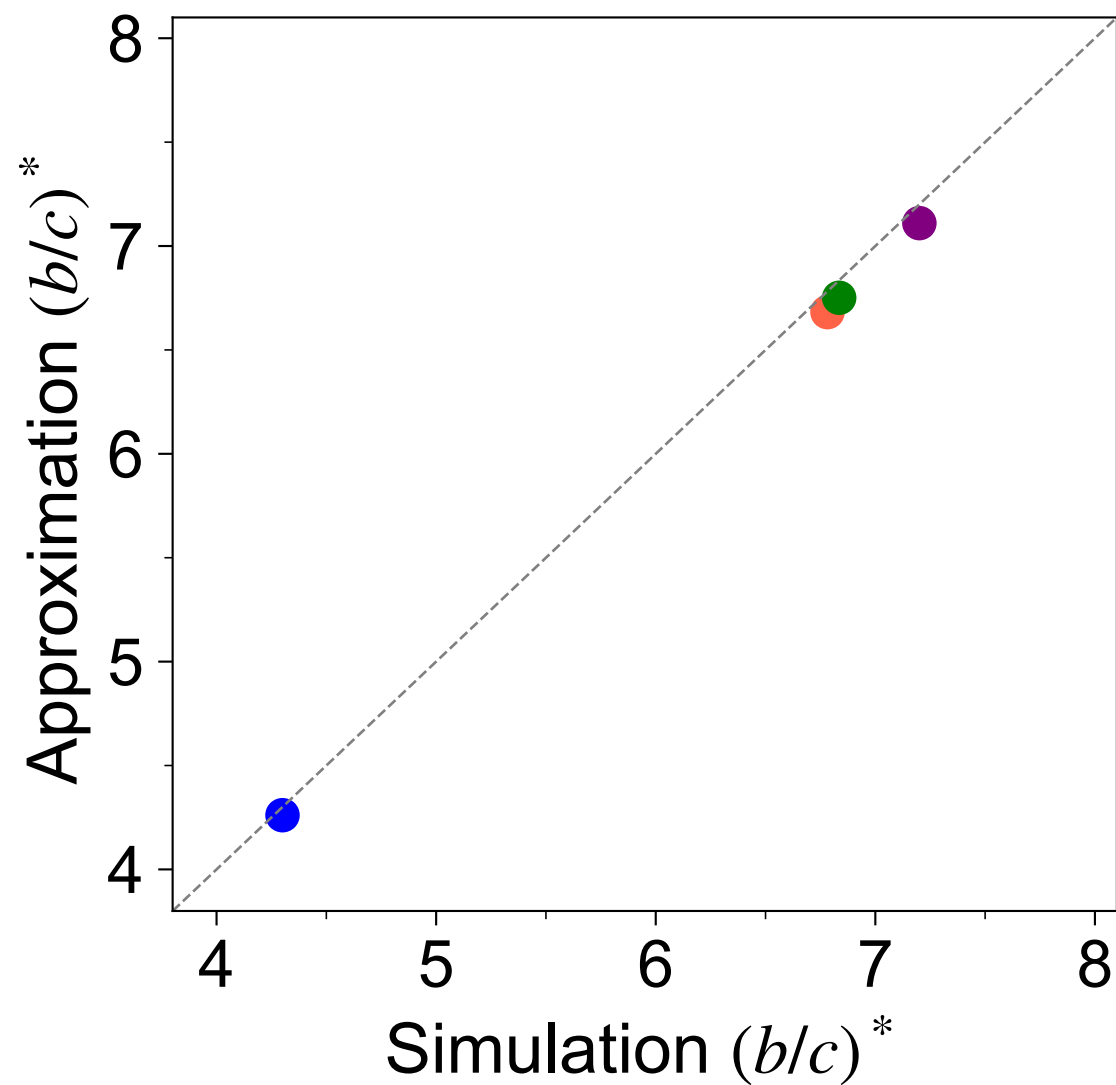

Given initial state

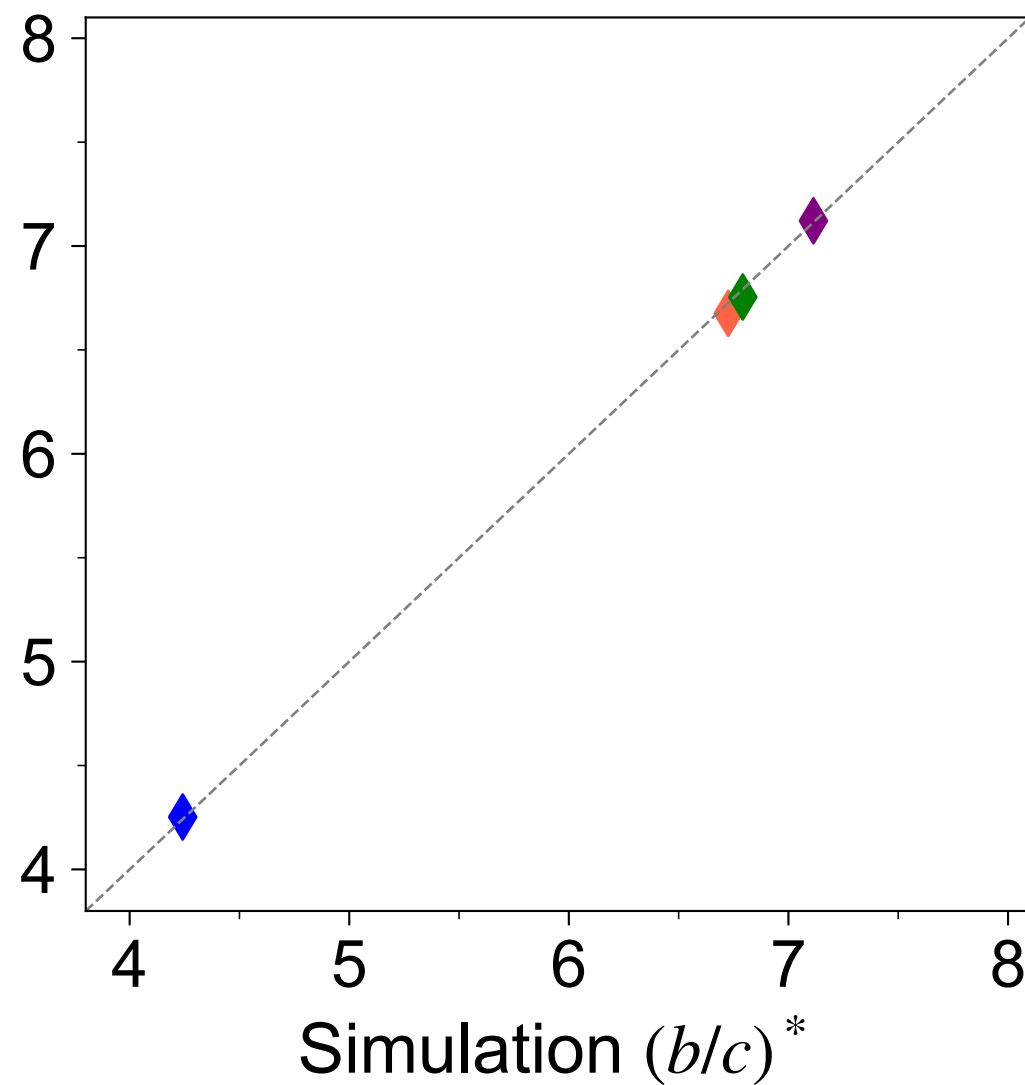

Temporal version

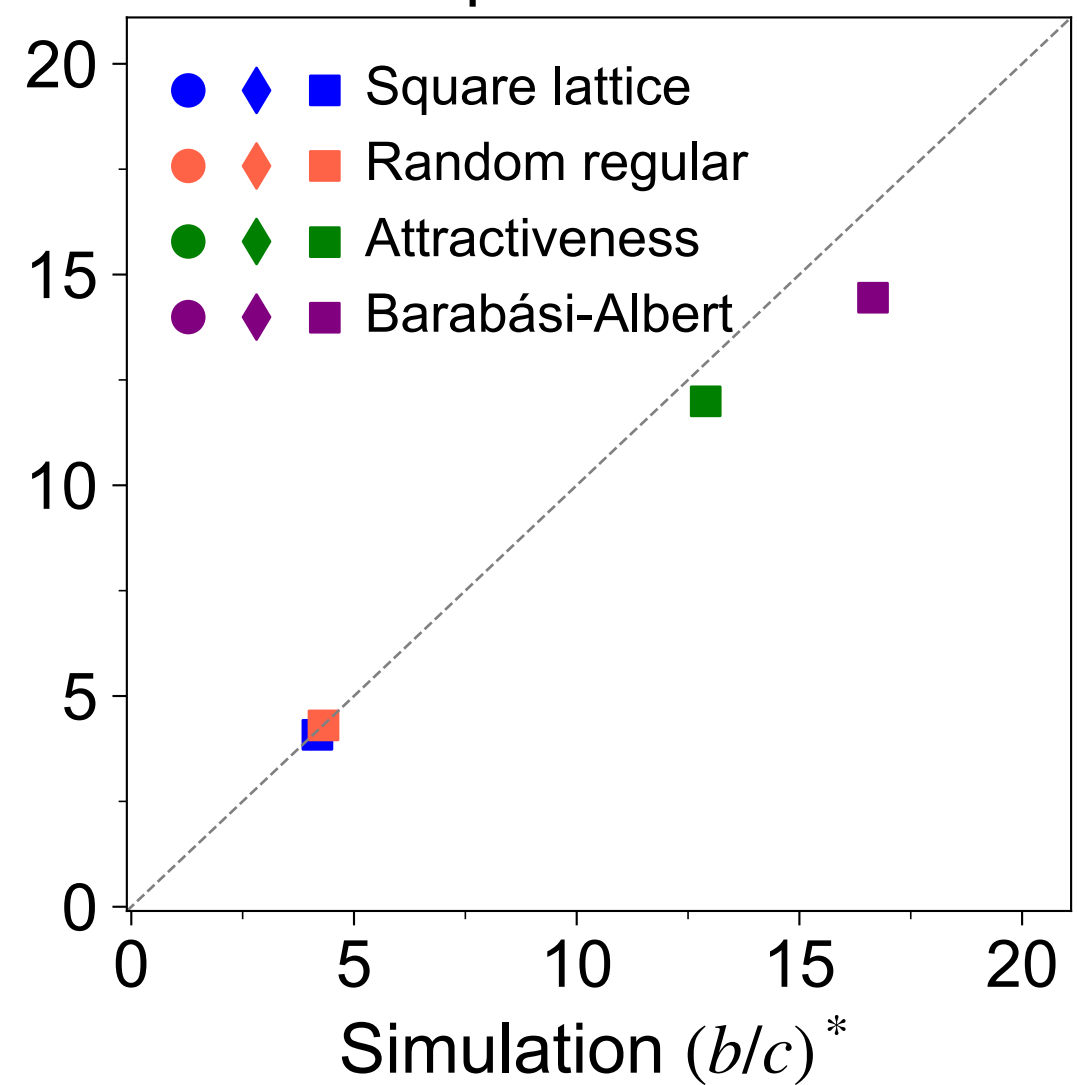

Supplement: S4 Fig — We consider the same network structure and the same initialization as S3 Fig. The results show that the mean-field approximation is also accurate for estimating the critical ratio of static networks and sequential temporal networks. (PDF) [file pcbi.1011333.s005.pdf]

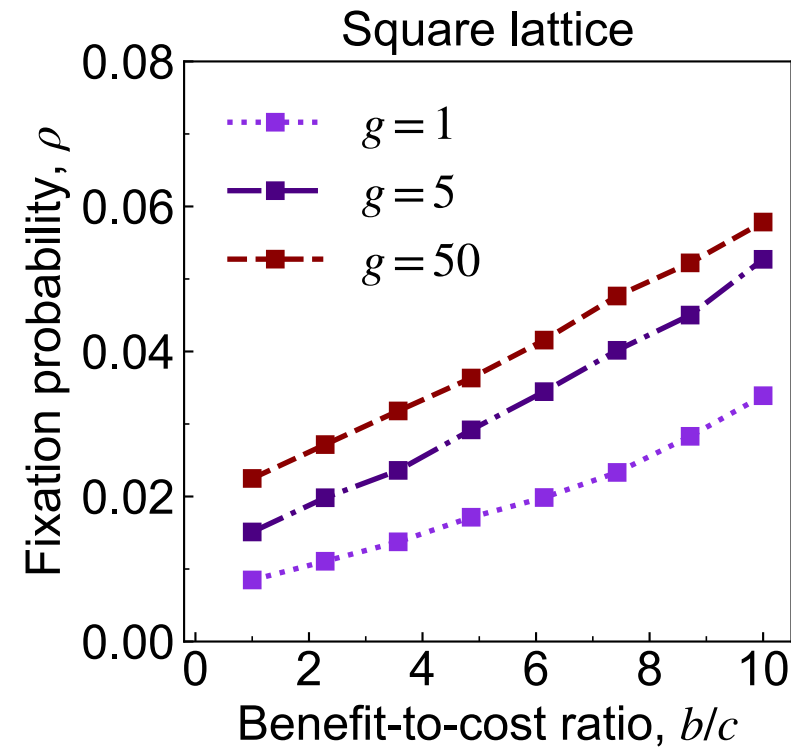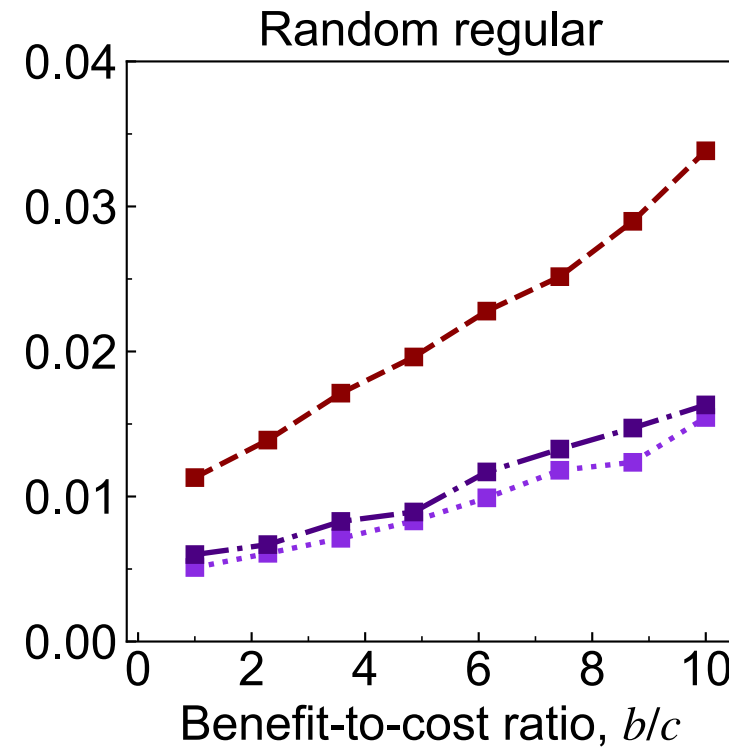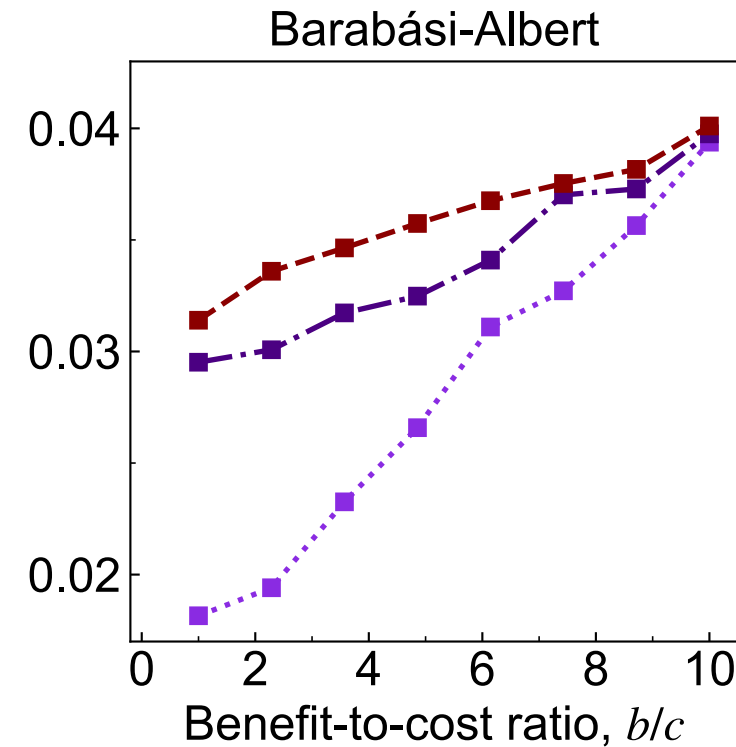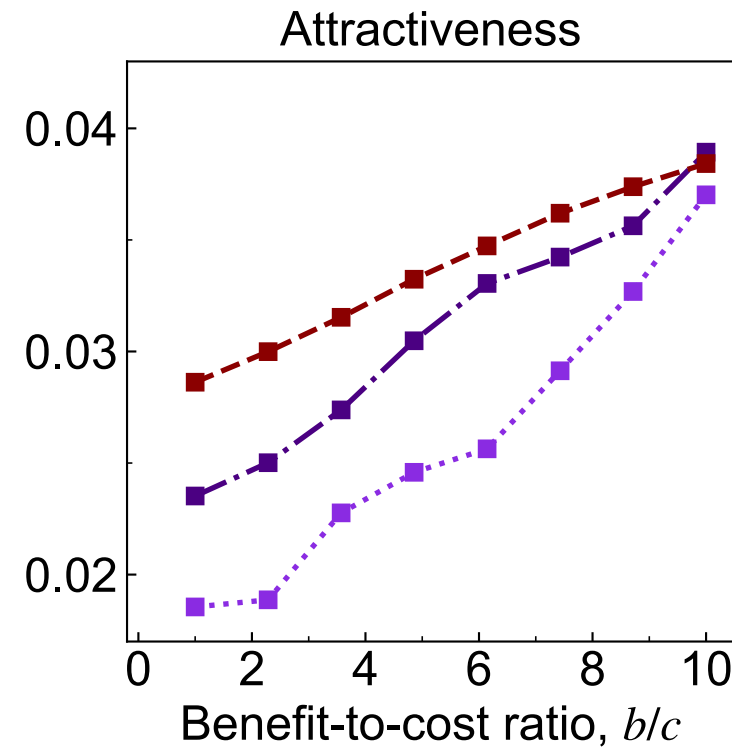

Supplement: S5 Fig — We consider the same network topologies as those in Fig 4 in the main text. The results show that the monotonicity of the fixation probability with respect to generation time g holds under weak selection for a wide range of benefit-to-cost ratio b/c. Parameter values are c = 1, δ = 0.015. (PDF) [file pcbi.1011333.s006.pdf]

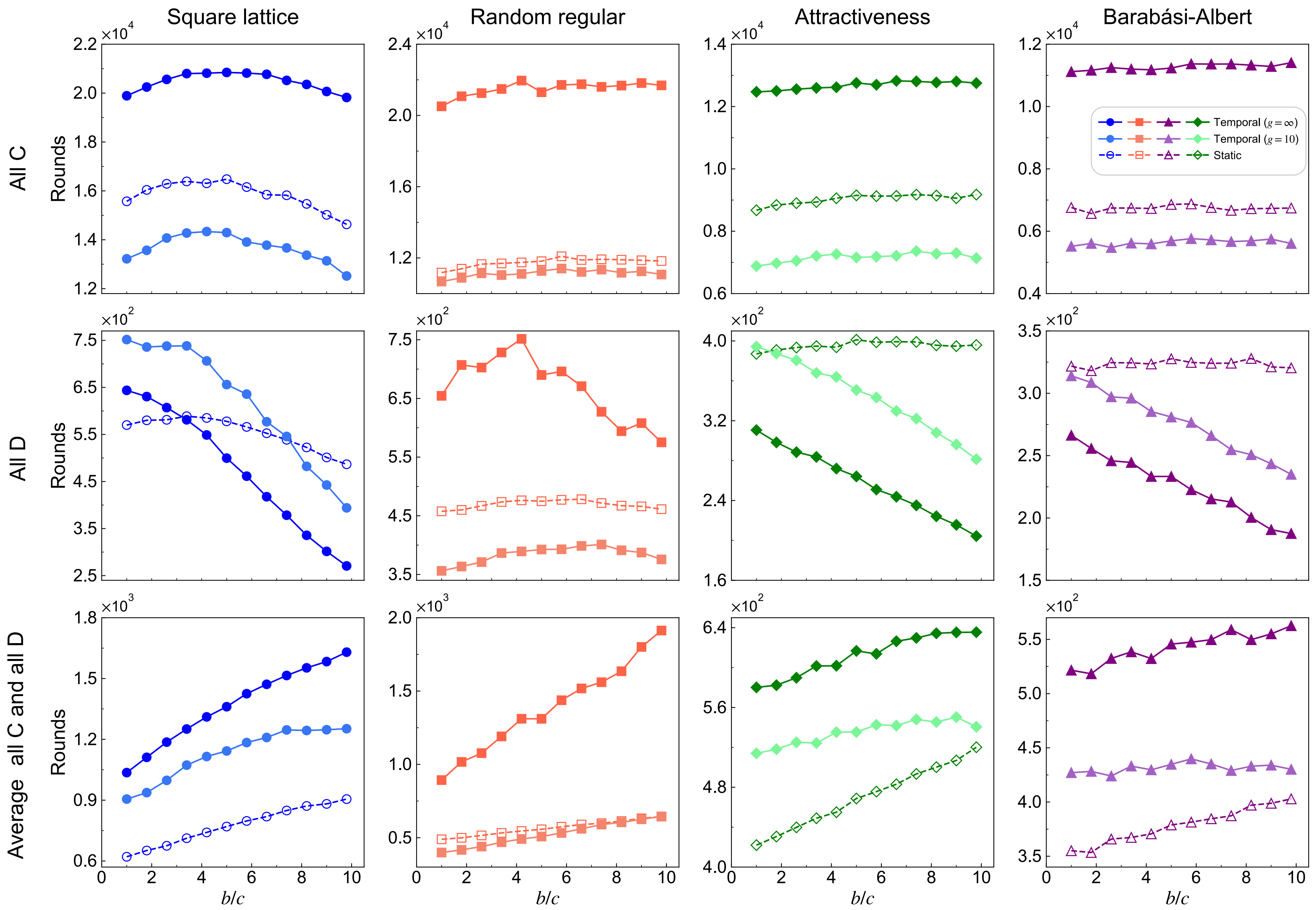

Supplement: S6 Fig — The conditional absorbing time and unconditional absorbing time of static networks and sequential temporal networks. We first focus on the conditional absorbing time of reaching the all-cooperator state C (first row). We find that when the number of rounds g = 10, the sequential temporal networks can both promote the evolution of cooperation and have lower absorbing time than static networks (light solid line). We also present the result of the conditional absorbing time of reaching the all-defector state D (second row) and the unconditional absorbing time (third row). Network structures and parameter values are the same as in Fig 4 in the main text. (PDF) [file pcbi.1011333.s007.pdf]

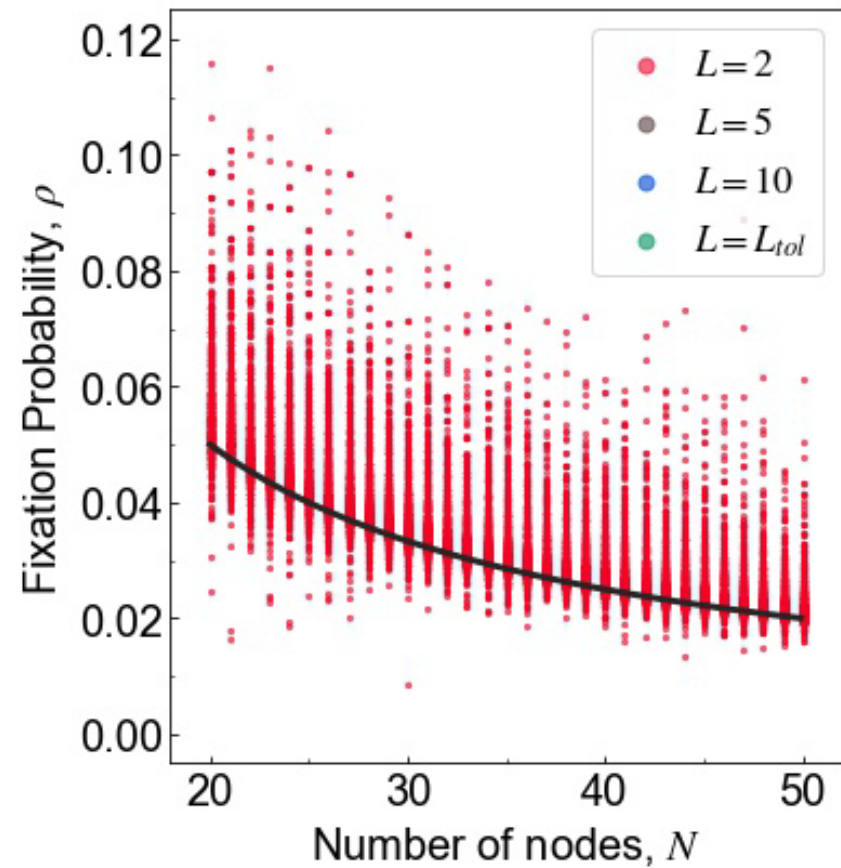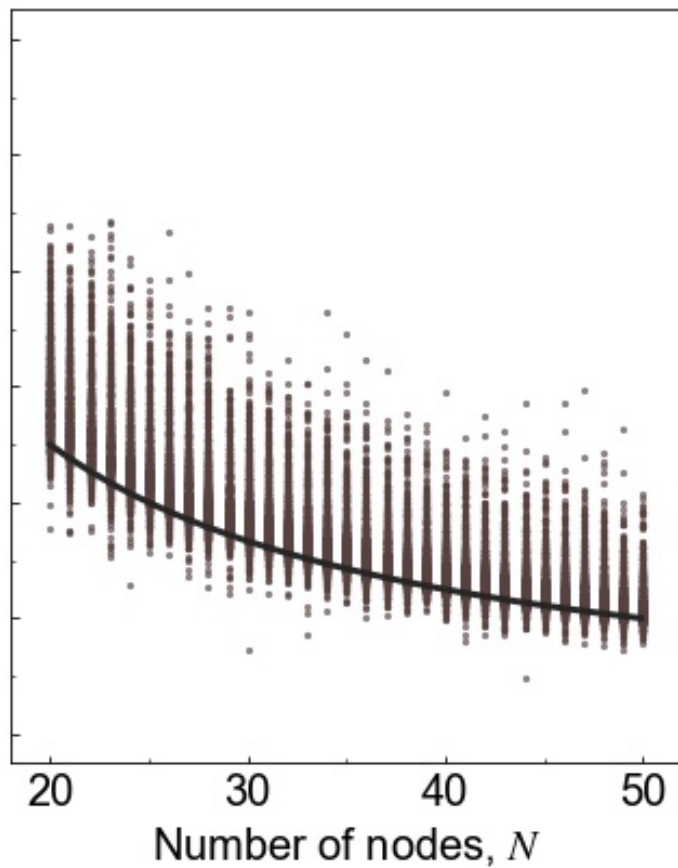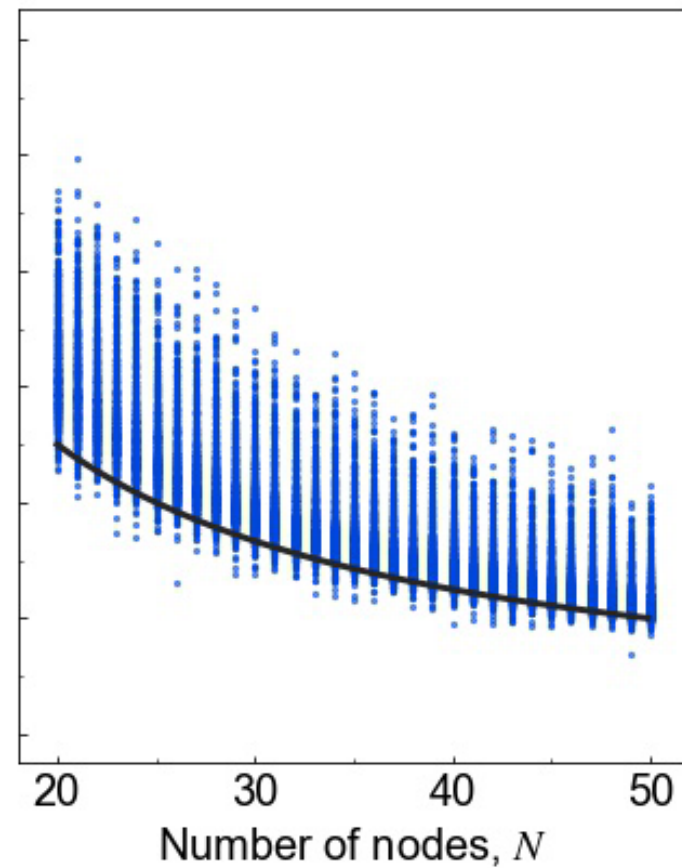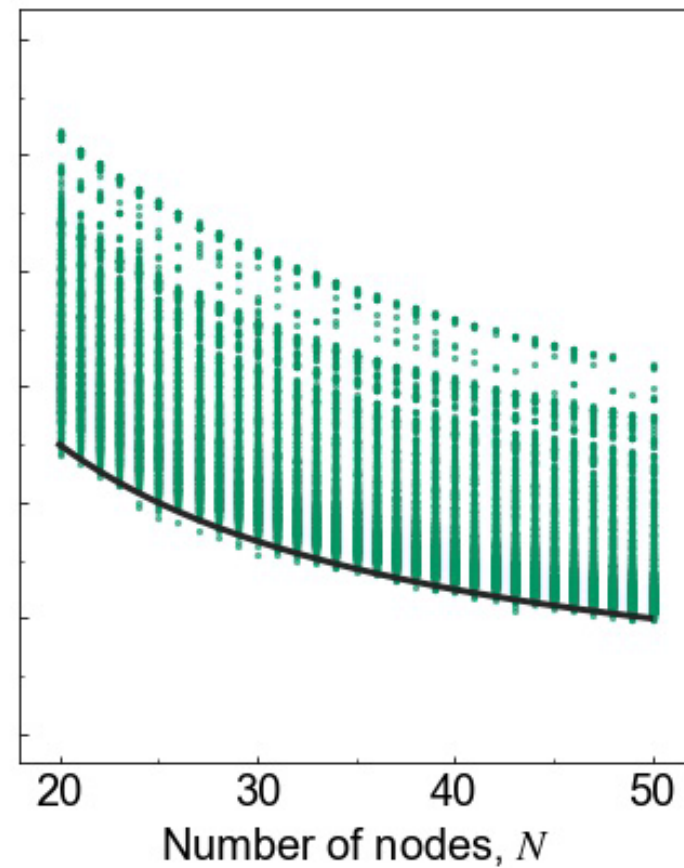

Supplement: S8 Fig — We analyze three classes of random graphs as static networks: Erdös-Rényi networks, Watts-Strogatz small-world networks with rewiring probability 0.3, and Barabási-Albert scale-free networks. For each class, we sample 104 graphs of size N and average degree k, where N is randomly selected from [20, 50] and k is randomly selected form [4, N/2]. For each static network, we apply Algorithm 1 in S1 Text to construct a sequential temporal network with length Ltol. Then, we use the sequential temporal network to generate new sequential temporal networks with length L ≤ Ltol, where the first and the final snapshots of the new networks are the same as the original one. The black lines show the fixation probabilities of static networks under neutral drift ((ρSμ)∘=1/N). The proportion of sequential temporal networks with a higher fixation probability ((ρTμ)∘>(ρSμ)∘) is monotonically increasing with L, rising from 83% when L = 2, to 87% when L = 5, and to 96% when L = 10, and to nearly 100% when L = Ltol. The number of sequential temporal networks with the same fixation probability (ρT∘=1/N) is monotonically decreasing with L, from 662 when L = 2, to 1 when L = 5, and to 0 when L = 10 and L = Ltol. (PDF) [file pcbi.1011333.s009.pdf]

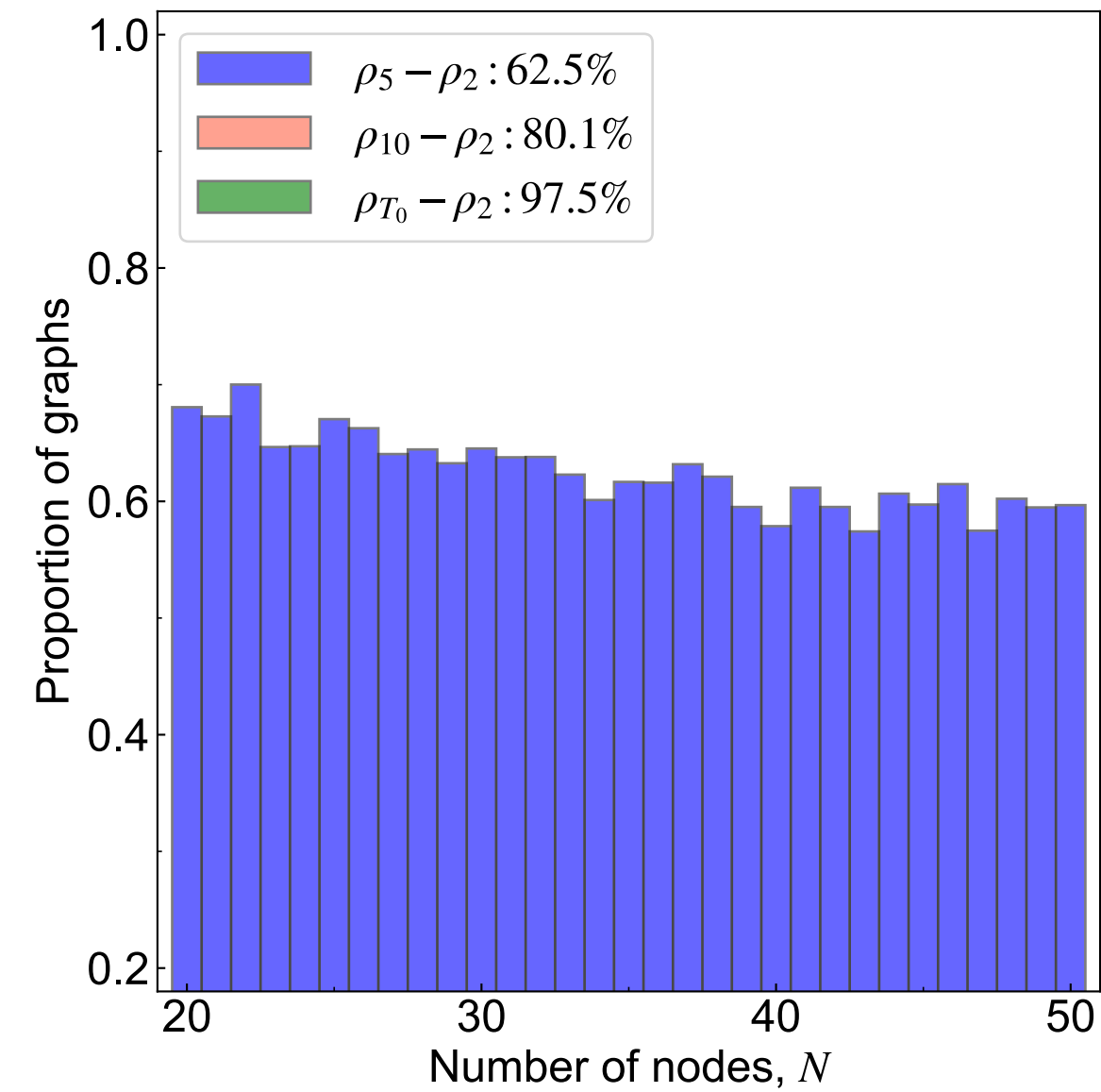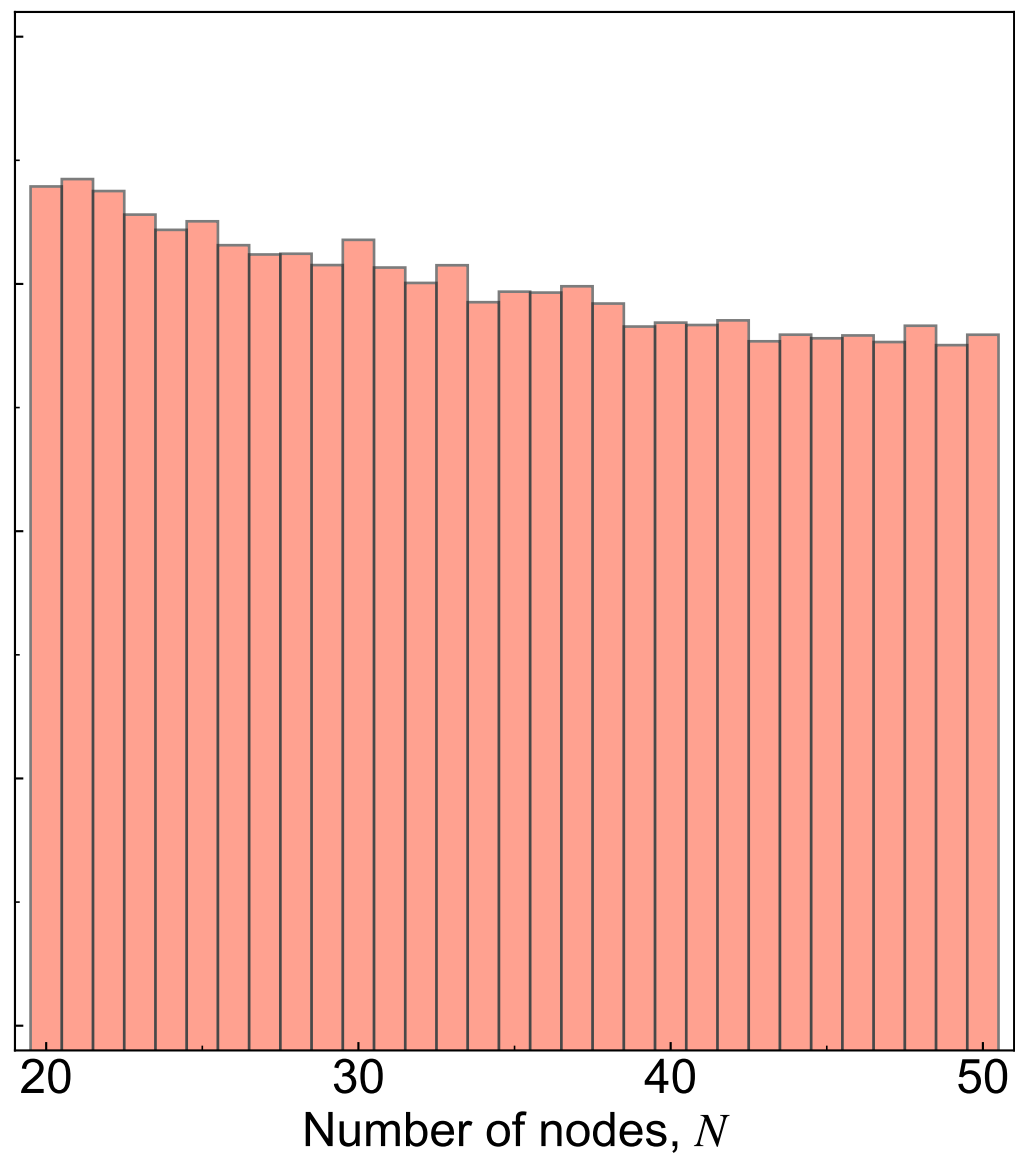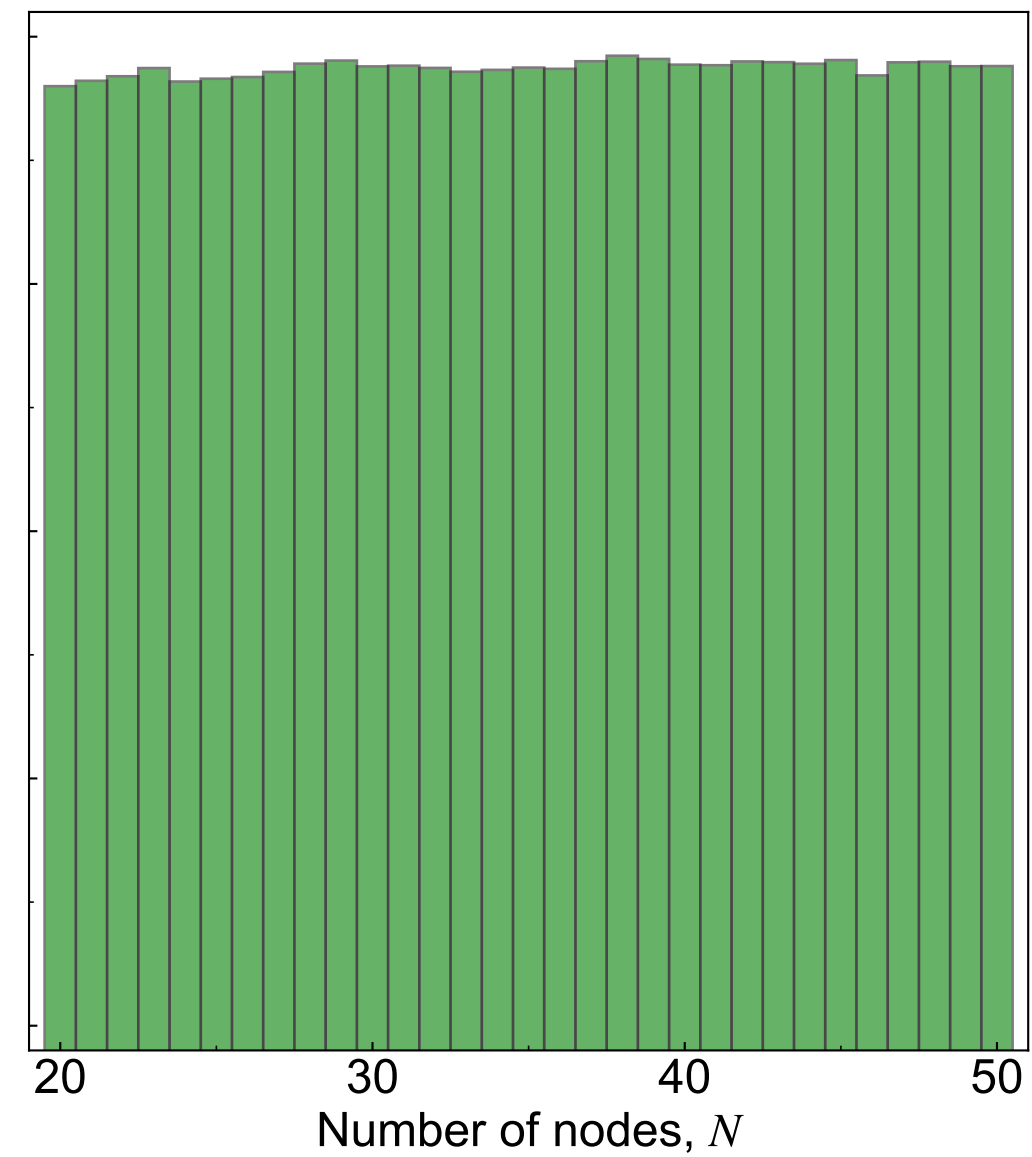

Supplement: S9 Fig — We analyze the same networks as in S8 Fig. We calculate the fixation probabilities for sequential temporal networks with different lengths. In general, the sequential temporal networks with longer lengths have higher fixation probabilities. (PDF) [file pcbi.1011333.s010.pdf]
