# Supplementary material for: Evolutionary dynamics on sequential temporal networks
Source: PLoS Comput Biol. 2023 Aug 7;19(8):e1011333. doi: 10.1371/journal.pcbi.1011333 (PMC10434888; doi:10.1371/journal.pcbi.1011333)
Supplement: S7 Fig — We consider two sequential temporal networks with the same initial and final snapshots. a, The increment of nodes for each pair of successive snapshots is one. In this case, the fixation probability of the sequential temporal network under neutral drift is higher than that of the static counterpart. b, The length of the sequential temporal network is 2, which means that there is no intermediate snapshot between the initial and final snapshots. In this case, the fixation probability of the sequential temporal network under neutral drift is lower than that of the static counterpart. (PDF) [file pcbi.1011333.s008.pdf]

**a**

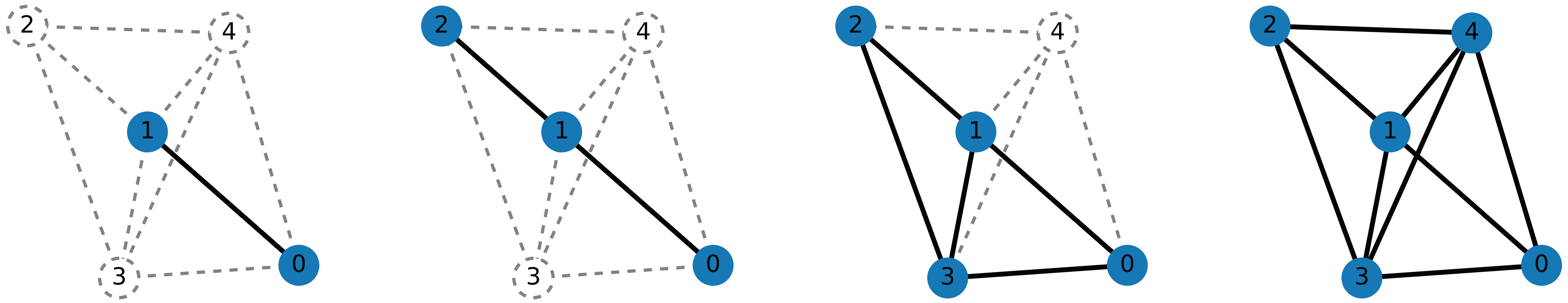


$$(\rho_{\mathcal{T}}^{\mu})^{\circ} = 0.204 > (\rho_{\mathcal{S}}^{\mu})^{\circ} = 0.2$$

**b**

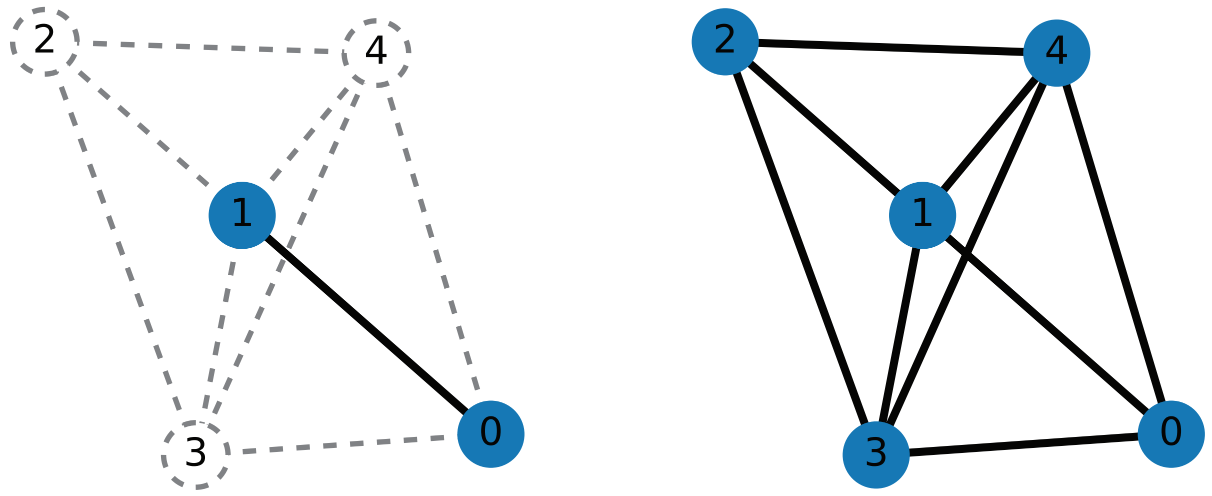


$$(\rho_{\mathcal{T}}^{\mu})^{\circ} = 0.194 < (\rho_{\mathcal{S}}^{\mu})^{\circ} = 0.2$$

/ Active / Inactive node — / - - - Active / Inactive edge
