# Supplementary material for: Evolutionary dynamics on sequential temporal networks
Source: PLoS Comput Biol. 2023 Aug 7;19(8):e1011333. doi: 10.1371/journal.pcbi.1011333 (PMC10434888; doi:10.1371/journal.pcbi.1011333)
Supplement: S1 Table — We analyze the same networks as in Fig 4. N is the number of nodes in static networks, L is the length of sequential temporal networks, and k is the average degree of static networks. (b/c)T* and (b/c)S* are the simulation-based critical benefit-to-cost ratios of sequential temporal networks and static networks, respectively. Approx. (b/c)T* and Approx. (b/c)S* are the corresponding approximate values with the mean-field approximation. (PDF) [file pcbi.1011333.s011.pdf]

Dataset	N	L	k	$(b/c)^*_{\mathcal{T}}$	Approx. $(b/c)^*_{\mathcal{T}}$	$(b/c)^*_{\mathcal{S}}$	Approx. $(b/c)^*_{\mathcal{S}}$
Square lattice	100	97	4	4.19	4.08	4.30	4.26
Random regular	100	97	6	4.31	4.30	6.78	6.68
Attractiveness	100	97	6	12.89	11.99	6.83	6.75
Barabsi-Albert	100	97	6	16.65	14.45	7.20	7.11
