# Supplementary material for: Evolutionary dynamics on sequential temporal networks
Source: PLoS Comput Biol. 2023 Aug 7;19(8):e1011333. doi: 10.1371/journal.pcbi.1011333 (PMC10434888; doi:10.1371/journal.pcbi.1011333)
Supplement: S2 Table — We analyze the same networks as in Fig 5. The implication of parameters is the same as S1 Table. (PDF) [file pcbi.1011333.s012.pdf]

Dataset	N	L	k	$(b/c)^*_{\mathcal{T}}$	Approx. $(b/c)^*_{\mathcal{T}}$	$(b/c)^*_{\mathcal{S}}$	Approx. $(b/c)^*_{\mathcal{S}}$
Scientific conference	403	246	47.46	53.31	53.81	70.68	70.42
Gallery	139	86	8.50	15.64	17.63	10.72	10.05
Workspace 13	95	18	82.42	-78.91	-76.62	-100.22	-102.86
Workspace 15	219	51	152.74	-184.24	-200.96	-326.06	-317.03
